# Keyless Semi-Quantum Point-to-point Communication Protocol with Low Resource Requirements

**DOI:** 10.1038/s41598-018-37045-0

**Published:** 2019-01-11

**Authors:** Haoye Lu, Michel Barbeau, Amiya Nayak

**Affiliations:** 1 University of Ottawa, School of Electrical Engineering and Computer Science (EECS), Ottawa, K1N 6N5 Canada; 2 Carleton University, School of Computer Science, Ottawa, K1S 5B6 Canada

## Abstract

Full quantum capability devices can provide secure communications, but they are challenging to make portable given the current technology. Besides, classical portable devices are unable to construct communication channels resistant to quantum computers. Hence, communication security on portable devices cannot be guaranteed. Semi-Quantum Communication (SQC) attempts to break the quandary by lowering the receiver’s required quantum capability so that secure communications can be implemented on a portable device. However, all SQC protocols have low qubit efficiency and complex hardware implementations. The protocols involving quantum entanglement require linear Entanglement Preservation Time (EPT) and linear quregister size. In this paper, we propose two new keyless SQC protocols that address the aforementioned weaknesses. They are named Economic Keyless Semi-Quantum Point-to-point Communication (EKSQPC) and Rate Estimation EKSQPC (REKSQPC). They achieve theoretically constant minimal EPT and quregister size, regardless of message length. We show that the new protocols, with low overhead, can detect Measure and Replay Attacks (MRA). REKSQDC is tolerant to transmission impairments and environmental perturbations. The protocols are based on a new quantum message transmission operation termed Tele-Fetch. Like QKD, their strength depends on physical principles rather than mathematical complexity.

## Introduction

Two full quantum capability devices can communicate securely with Quantum Key Distribution (QKD)^1–4^. In this protocol, two communicants have to be armed with advanced quantum components including quantum registers, programmable quantum circuits and quantum generators. Most of them can only function under stable and well-configured environments and occupy large space. So, it is challenging to implement secure communications on portable devices. On the other hand, quantum computers can efficiently break RSA cryptosystem^5^, the security foundation of almost all classical communication protocols. Thence, the communication security of portable devices is in imminent danger of collapse.

Semi-Quantum Communication (SQC) intends to break the predicament by limiting the quantum capability of the receiver without dampening the transmission security. The quantum components for realizing limited quantum capability can be designed compact, simple and robust so that they could be integrated into a portable device. The discussions start from two Semi-Quantum Key Distribution (SQKD) protocols reported by Boyer *et al*.^6,7^. Compared with QKD, the receiver Bob needs only to perform four quantum operations: (1) generate quantum bits (qubits) in the Z-basis, (2) measure qubits in the Z-basis, (3) permute qubits and (4) access quantum channel. These two new protocols secure the communications by randomizing measurement basis and Bob’s treatment on the qubits he receives. For concealing Bob’s behavior, reordering of the qubits is also required. In 2011, Jian *et al*.^8^ proposed a new SQKD protocol that improves qubits efficiency (the message length with respect to the number of qubits sent by Alice) from the original 12.5% to roughly 50% by using entangled qubits. But the Entanglement Preservation Time (EPT) for implementing the protocol is at least linear to the length of the message. So is the quantum bit register (quregister) size. Li *et al*.^9^ showed that Bob’s quantum computation task can be delegated to a third party quantum server in semi-quantum communications at the cost of a low qubit efficiency (6.25%). In 2015, Luo and Hwang^10^ proposed a new protocol showing that the Public Bidirectional Authentic Classical Channel (PBACC) is unnecessary if the two communicants have a pre-shared key. However, besides a even longer EPT and a low qubit efficiency (12.5%), a larger quregister size is required for each data bit. A similar pre-shared key based protocol proposed by Almousa and Barbeau^11^ shows that Bob does not need to store any qubits, but the linear EPT persists. Recently, more work concerning SQC is reported^12–15^.

All the aforementioned protocols^6–12^ suffer from low qubit efficiency. Most of them have significant large linear quregister size overhead and require permutation of qubits^6–8,10,11^. Regarding the protocol involving entangled qubits^8,10,11^, the quantum EPT is at least linear. Although a six-hour record has been achieved by Zhong *et al*. utilizing europium ion implanted in a crystal^16^, entanglement time declines considerably should the entangled photons be propagated in an optical fiber (the most common implementation of quantum communication protocols)^17^. Besides, involving permutations on qubits (not practical shortly) dooms to a low transmission efficiency and reliability. Considering that the unusual materials (for instance, coupled electron^18^ and ultracold atoms^19^) are necessary for the implementation of quregisters, a commercial quantum network based on them is not feasible in a near future.

This paper reports a new Semi-Quantum Direct Communication (SQDC) protocol and a rate estimation version, named Economic Keyless Semi-Quantum Point-to-Point Communication (EKSQPC) and Rate Estimation EKSQPC (REKSQPC), that address all the aforementioned issues. An innovative operation, called Tele-Fetch (TF), utilizes entangled qubit pairs to transmit messages. It is at the core of the One-Bit Protocol (OBP). The results of measurements on the pairs fall in a predesigned set of values because of the entanglement, but do not carry any useful information. The design makes the OBP functioning without a pre-shared key and fully resistant to information leakage even if the qubits are intercepted. Besides, the protocol uses the same quantum circuit as the one to detect the Measure and Replay Attack (MRA) (called MRA Detection (MRAD)) and thus, not only saves the quantum resources but also becomes the cornerstone of the EKSQPC and REKSQPC protocols. Because Alice performs the same quantum procedures in both protocols (OBP or MRAD), Bob does not need to communicate with Alice until all quantum procedures (Alice’s and Bob’s) are completed. Alice and Bob execute MRADs using a small portion of the measurement results before using the PBACC to translate the rest into valid messages. The protocol is proved fully secure under the assumption that MRA are always detectable. As the pivot to secure the messages is a successful detection of MRA, we show that, with only 15 probing bits, the attack detection success rate can achieve 0.995 (under the assumption that the adversary Eve has 0.6 possibility to attack a qubit). The security of the protocol is enhanced considerably if a few more probing bits are added. The qubit efficiency asymptotically reaches 100% with the message length. The implementation of the EKSQPC protocol has low requirements on quantum resources. In particular, the quregister size required by Alice is as low as one, and the required EPT is *C* + 2*T* (where *C* is the time that Alice takes to generate, send and receive the qubits; and *T* is the one-way time for the qubits to travel between Alice and Bob). We prove that both the quregister size and EPT reach the theoretical minimums.

Considering that the entanglement of qubits may not always persist during the transmission of qubits, we assume that there is a probability $\omega$ that the entanglement involving a qubit is destroyed as it can be disturbed by hardware imperfection and environmental disturbance. Under this assumption, we design a statistical test to compare $\omega$ with the probability that a qubit is attacked or disturbed. When a significant difference is observed, Alice concludes that Eve perpetrated attacks and aborts the execution of the protocol. Therefore, the communication is not eavesdropped successfully. Compared with the original EKSQPC, more probing bits are required to achieve the same detection success rate; however, the overhead is still low. In particular, our simulation results reveal that 60 probing bits are enough to detect almost all attacks when $\omega$ is unknown. If the rate is given, then 40 probing bits are enough to achieve the same detection success rate.

This paper is a revised and extended version of a preliminary workshop paper^20^ in which we introduced the original EKSQPC protocol. Compared to the workshop paper, this paper articulates the original protocol as well as its analysis with more details. Based on this, we report an upgraded and practical version, REKSQPC, with its security, resource requirements and transmission efficiency analysis. Moreover, we also provide a detailed comparison with other typical SQKD and SQDC protocols.

The rest of the paper is organized as follows. In Section 2, we review Bell measurement and MRAD, which are integrated in the new protocol. In Sections 3 and 4, we introduce our new protocols including a rate estimation version taking into account the probability that a qubit is disturbed. We also do a security analysis and discuss simulation results. In Section 5, we talk about their quantum resource requirements and transmission overhead. Finally, we draw the conclusions in Section 6.

## Background

The section starts from a brief review of EPR pairs states and Bell measurement on which our new protocol heavily relies. Then we introduce the MRA as well as its detection algorithm (MRAD) that secures the data transmission of the new protocol.

In this paper, classical bits (cbits) are denoted by lowercase English letters, and a cbit sequence is represented by an uppercase English letter over a tilde. For instance, $\mathop{M}\limits_{ \tilde {}}={m}_{1}{m}_{2}\cdots {m}_{t}$ is a cbit string of length *t*. Qubits are denoted by Greek letters and Bell states by Bold English capital letters.

### EPR pairs and Bell measurement

**Figure 1:**
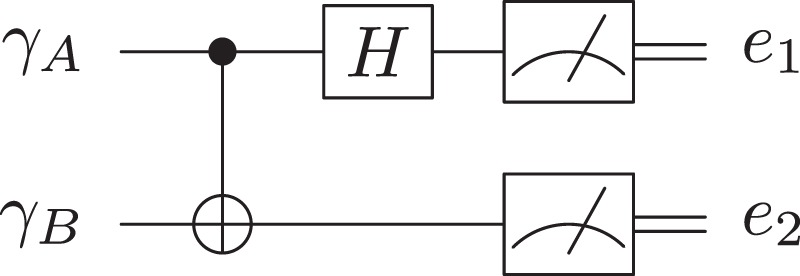
The Bell measurement Circuit.

A pair of qubits that are together in Bell state is called an EPR pair. Bell states have four types: $|{{\rm{\Phi }}}^{+}\rangle =\frac{1}{\sqrt{2}}\cdot (|00\rangle +|11\rangle )$, $|{{\rm{\Phi }}}^{-}\rangle =\frac{1}{\sqrt{2}}\cdot (|00\rangle -|11\rangle )$, $|{{\rm{\Psi }}}^{+}\rangle =\frac{1}{\sqrt{2}}\cdot (|01\rangle +|10\rangle )$, and $|{{\rm{\Psi }}}^{-}\rangle =\frac{1}{\sqrt{2}}\cdot (|01\rangle -|10\rangle )$; we can use the Bell measurement (B.M.) (Fig. 1) to identify them. The inputs of the circuit are two qubits *γ*_*A*_ and *γ*_*B*_, and the outputs are two cbits *e*_1_ and *e*_2_.

If *γ*_*A*_*γ*_*B*_ is an EPR Pair (Bell state), then the outputs are deterministic and listed in Table 1; otherwise, *γ*_*A*_*γ*_*B*_ is mapped into a Bell state stochastically. For *γ*_*A*_*γ*_*B*_ equal to $|00\rangle$, $|01\rangle$, $|10\rangle$ or $|11\rangle$, the distributions of the outputs are listed in Table 2.

**Table 1:** Bell measurements on Bell states.

*γ*_*A*_ *γ*_*B*_	Output (*e*_1_*e*_2_)	*γ*_*A*_ *γ*_*B*_	Output (*e*_1_*e*_2_)
$|{{\rm{\Phi }}}^{+}\rangle$	$00$	$|{{\rm{\Phi }}}^{-}\rangle$	10
$|{{\rm{\Psi }}}^{+}\rangle$	01	$|{{\rm{\Psi }}}^{-}\rangle$	11

**Table 2:** Bell measurement results of $|00\rangle$, $|01\rangle$, $|10\rangle$ and $|11\rangle$; there is 0.5 possibility for each output.

*γ*_*A*_ *γ*_*B*_	Output (*e*_1_*e*_2_)	*γ*_*A*_ *γ*_*B*_	Output (*e*_1_*e*_2_)
$|00\rangle$	$00$	$|01\rangle$	01
	10		11
$|10\rangle$	01	$|11\rangle$	00
	11		10

### Measure and Replay Attack and the method of detection

A simplified version of Man-In-The-Middle Attacks (MITMs) is called replay attack. The attacker Eve deceives the truthful listener(s) by replaying messages outside the expected context so that the listener believes that the protocol has been executed successfully^21^.

We can perpetrate a similar attack in the context of quantum communications. Assume that qubits sent or received by Alice and Bob can be intercepted by Eve. As a qubit is sent to Bob by Alice through the quantum channel, Eve uses the Z-basis to measure it. If the measurement result is zero, then Eve sends $|0\rangle$ to Bob; otherwise, she sends $|1\rangle$. As the replay of the message follows the measurement, we call the attack the Measure and Replay Attack (MRA)^11^.

The following method demonstrates how to utilize the EPR pairs and Bell measurement to detect MRA. The Luo and Hwang’s protocol^10^ and Almousa and Barbeau’s protocol^11^ also apply similar ideas for the attack detection.

**MRA Detection (MRAD)**:

D1 Alice randomly picks a cbit $i=0$ or 1, based on which she generates an EPR pair ${\mathbb{E}}$ (if $i=0$, ${\mathbb{E}}=|{{\rm{\Phi }}}^{+}\rangle$; else, ${\mathbb{E}}=|{{\rm{\Psi }}}^{-}\rangle$).

D2 ${\mathbb{E}}$ consists of two qubits, *γ*_*A*_ and *γ*_*B*_. Alice keeps *γ*_*A*_ and sends *γ*_*B*_ (named the probing bit) to Bob.

D3 *γ*_*B*_ is reflected by Bob to Alice.

D4 After receiving ${\gamma ^{\prime} }_{B}$, Alice applies Bell measurement (Fig. 1) on ${\gamma }_{A}{\gamma ^{\prime} }_{B}$ to obtain *e*_1_ and *e*_2_.

D5 The combination of *e*_1_ and *e*_2_ indicates the EPR pair that the circuit measured. We consider the protocol secure (denoted by zero) if the measured EPR pair agrees with the one Alice produced in Step D1. If not, an MRA is detected (denoted by one).

#### **Example 1**.

*Suppose Alice and Bob implement* MRAD *for* MRA *detection*. *Without loss of generality*, *assume Alice picks* $i=1$ *and thus produces a corresponding EPR pair* ${\mathbb{E}}=|{{\rm{\Psi }}}^{-}\rangle =\frac{1}{\sqrt{2}}\cdot (|01\rangle -|10\rangle )={\gamma }_{A}{\gamma }_{B}$. *Alice intends to send γ*_*B*_*to Bob*; *however*, *Eve intercepts and measures it and gets the measurement result* $r=0$. *Simultaneously*, *γ*_*A*_*retained by Alice collapses to* $|1\rangle$ *due to the entanglement*. *Eve produces a new qubit* ($|0\rangle$) *correspondingly and send it to Bob*. *Bob does nothing but reflects it back to Alice*. *γ*_*A*_*and the received* $|0\rangle$ *are paired together and measured by Alice using the Bell measurement circuit*. *Notice that* ${\gamma }_{A}$ *has collapsed to* $|1\rangle$. *So the qubit pair measured by the circuit is* ${\gamma }_{A}{\gamma }_{B}=|10\rangle$. *By* Table 2, *we have* 50 *percent possibility to get* ${e}_{1}{e}_{2}=01$ (*and so deduce that the input is* $|{{\rm{\Psi }}}^{+}\rangle$ *by* Table 1, *a true positive*) *and to get* ${e}_{1}{e}_{2}=11$ (*and thus deduce that the input is* $|{{\rm{\Psi }}}^{-}\rangle$, *a false negative*).

The example shows that if Alice picks $i=1$ and the measurement result *r* of Eve is zero, there is 50 percent possibility for Alice to deduce that the protocol is secure although the attack is perpetrated. By Table 2, we can draw the same conclusion for any choice of *i* and *r*. Hence, Lemma 1 and Theorem 1 follow.

#### **Lemma 1**.

*Provided that Eve attacks the probing bit*, *there is* 0.5 *possibility for MRAD to detect an MRA*.

#### **Theorem 1**.

*If MRAD are repeated n times*, *we have* 1 − 0.5^*n*^*probability to detect MRA given that Eve attacks n probing bits*.

#### *Proof*.

$$Pr[{\rm{detect}}\,{\rm{MRAs}}]\mathrm{=1}-Pr{[{\rm{MRAD}}{\rm{fails}}]}^{n}\mathrm{=1}-{0.5}^{n}$$ $$\square $$

#### **Remark 1**.

*MRAD essentially checks whether the probing bits sent by Alice had been measured by anybody else*, *but it cannot tell who measured them*. *It can detect MRA only because Alice knows that Bob does not measure probing bits*. *Therefore*, *if any measurement is detected*, *it must be due to an attack*.

Because, the operations defined in Steps D1 and D5 are applied again in the sequel, we define them formally as follows,

#### **Definition 1**

(Generating corresponding EPR pairs (*F*), Step D1). *Function F maps a* cbit *to an EPR pair such that* $0\mapsto |{{\rm{\Phi }}}^{+}\rangle$, and $1\mapsto |{{\rm{\Psi }}}^{-}\rangle$

#### **Definition 2**

(Alice Examines (AE), Step D5). *The function* $AE({e}_{1},{e}_{2},i):{\{0,1\}}^{3}\to \{0,1\}$ *equals zero if* ${e}_{1}={e}_{2}=i$; *otherwise*, *it equals one*. *Recall that zero and one indicate negative and positive detection results*, *respectively*.

## New Protocol

In this section, we propose a new SQDC protocol called EKSQPC. We start the discussion with an introduction to a data transmission protocol called OBP, which is a building block of EKSQPC (not self-contained). Assuming that there are no MRA, we show that OBP is secure (Theorem 2). In the design of OBP, Bell measurement seems redundant. It is intended for sharing the quantum circuit with MRAD (Remark 2). The considerable benefits of this design are discussed in Section 5. To meet the assumption of Theorem 2, we integrate MRAD and OBP to get EKSQPC. If we assume that EKSQPC detects all MRA, then it is provably secure (Theorem 4).

### One-Bit Protocol (OBP)

**Protocol 1 (OBP)**: *A one*-*bit message m* (*zero or one*) *is sent to Bob by Alice*. *We need a PBACC as well as a Public Bidirectional Quantum Channel* (*PBQC*). *The protocol functions as follows*:

1. *Alice randomly picks a cbit i and generates a corresponding EPR pair* ${\mathbb{E}}=F(i)$ (*Definition 1*) *consisting of two qubits* (*denoted by* γ_*A*_*and* γ_*B*_).
2. *Alice keeps γ*_*A*_*and sends γ*_*B*_*to Bob*.
3. *Upon reception, the qubit γ*_*B*_*is measured by Bob in the Z*-*basis with the measurement result u*_*B*_ (*simultaneously*, *γ*_*A*_*collapses because of the entanglement with γ*_*B*_). *At the same moment*, *Bob sends a pre*-*prepared qubit* ${\gamma }_{B}^{\ast }=0$ *to Alice and informs her that he has measured γ*_*B*_*via the PBACC*.
4. *Alice pairs γ*_*A*_ (*retained in Step P1*) *with* ${\gamma ^{\prime} }_{B}$ *and performs a Bell measurement on* ${\gamma }_{A}{\gamma }_{B}^{\ast }={\gamma }_{A}|0\rangle$ *to get e*_1_*and e*_2_.
5. *According to* Table 2, ${e}_{1}{e}_{2}=00\,\,or\,10$ *implies that* ${\gamma }_{A}=|0\rangle$, *and* ${e}_{1}{e}_{2}=01\,\,or\,11$ *indicates that* ${\gamma }_{A}=|1\rangle$. *Combining* ${\gamma }_{A}$ *with the EPR pair* ${\mathbb{E}}$ *Alice selected* (*recorded by i*) *in Step P1*, *Alice learns the measurement result u*_*B*_*of Bob in Step P3*. *In particular*, *if* $i=0$, *then the EPR pair she generated was* $|{{\rm{\Phi }}}^{+}\rangle$. *Then* ${\gamma }_{A}=|0\rangle$ *implies* ${u}_{B}=0$, *and* ${\gamma }_{A}=|1\rangle$ *implies* ${u}_{B}=1$. *Similarly*, *if* $i=1$, *the EPR pair that Alice generated was* $|{{\rm{\Psi }}}^{-}\rangle$. *Then if* ${\gamma }_{A}=|0\rangle$, ${u}_{B}=1$; *else*, ${u}_{B}=0$.
6. *Provided that* ${u}_{B}=m$, *Alice informs Bob*, *via the PBACC*, *that* ${u}_{B}$ *is the correct value*. *Otherwise*, *she informs Bob to take* $1-{u}_{B}$.

#### **Remark 2**.

*The pre*-*generated* qubit ${\gamma }_{B}^{\ast }=0$ *in Step P3 is unnecessary to implement* OBP. *So is the Bell measurement in Step P4*. *In fact*, *in Step P3*, *Bob only needs to notify Alice that he has measured* ${\gamma }_{B}$, *and*, *in Step P4*, *Alice simply uses the Z*-*basis to get the value of* ${\gamma }_{A}$. *Here*, *we intendedly implement OBP with redundant operations so that the new protocol* (*EKSQPC*, *introduced in Section 3*.*2*) *can use a single quantum circuit to implement both the attack detection* (*MRAD*) *and data transmission* (*OBP*) *protocols*. *We discuss the design and its benefits in details in Section 3*.*2*, *and more performance analysis is conducted in Section 5*.

The actions specified in Steps P5 and P6 are used subsequently. We define them formally as follows. In Step P5, Alice learns ${r}_{B}$ held by Bob with no contact. So the function is called Tele-Fetch.

#### **Definition 3**

(Tele-Fetch). *With the parameters* ${e}_{1}$, ${e}_{2}$ *and* $i$ (*Step P1*), *function Tele*-*Fetch TF* $({e}_{1},{e}_{2},i)$ *returns the value of* ${r}_{B}$ (*zero or one*) *based on the rule contained in Step P5*.

Besides, in Step P6, Alice rectifies the measurement result ${r}_{B}$ of Bob. As a result, we call the procedure Rectify.

#### **Procedure 1**

(Rectify). *Based on the single bit message* $m$ *and value of* ${u}_{B}$ (*acquired in Step P5*), *Alice informs Bob to apply the proper operation on* ${u}_{B}$ *by sending either the signal KEEP or FLIP via the PBACC*. *If KEEP is received*, *Bob considers* ${u}_{B}$ *as the message Alice sends*; *if not*, *he takes* $1-{u}_{B}$.

The next theorem discusses the security of OBP.

#### **Theorem 2**.

*As long as Bob gets the qubit* ${\gamma }_{B}$ *sent by Alice without MRA*, *OBP is secure*.

#### *Proof*.

By assuming the absence of MRA, we essentially assume that Steps P1 to P3 are secure (only a confirmation is sent by Bob in Step P3). No communication happens in Step P4 and P5. The last step involves a message sent by Alice which is irrelevant to the one-bit message *m*. So Step P6 is sheltered, too. Thence, to sum up, OBP is secure.$\square$

#### **Remark 3**.

*An authentic classical channel is the prerequisite for the security of OBP*. *In the communication of Alice and Bob*, *it is significant to verify their identities and to ensure that their unencrypted messages are not altered*. *Namely*, *they should be resistant to MITMs*.

Theorem 2 shows that only when there is no MRA, OBP is secure. However, OBP has no capability to detect MRAs. Notice that MRAD can detect MRAs and thus can secure the data transmission of OBP by Theorem 2. If we combine OBP and MRAD together, we get the protocol discussed in the following subsection.

### Economic Keyless Semi-Quantum Point-to-Point Communication (EKSQPC)

We implement the protocol EKSQPC over the hardware of OBP. Specifically, there are a PBACC and a PBQC linking Alice and Bob. The following four procedures contain all the activities demanding quantum resources in EKSQPC.

#### **Procedure 2**

(Alice sends). *In the* ${k}^{th}$ *transmission of Alice*, *she picks a random* cbit *i*_*k*_*and stores it in a classical register*. *Then she produces an EPR pair* $F({i}_{k})$, *keeps the first qubit* ${\gamma }_{kA}$ *and transmits the second qubit* ${\gamma }_{kB}$ *to Bob*.

#### **Procedure 3**

(Bob measures). *After receiving the* ${k}^{th}$ *qubit from Alice*, *Bob measures it in the* $Z$-*basis and gets the result* ${u}_{k}$. *At the same moment*, *a pre*-*prepared qubit* $|0\rangle$ *is sent back to Alice*. *Furthermore*, *Bob takes the record that he measured the* ${k}^{th}$ *qubit*.

#### **Procedure 4**

(Bob reflects). *The* ${k}^{th}$ *qubit from Alice is reflected back without measurement by Bob*. *Also*, *he takes the record that he reflected the* ${k}^{th}$ *qubit he received*.

#### **Procedure 5**

(Alice measures). *Alice receives the* ${k}^{th}$ *qubit* ${\gamma }_{kB}^{\ast }$ *and performs Bell measurement on* ${\gamma }_{kA}{\gamma }_{kB}^{\ast }$ (${\gamma }_{kA}$ *was retained by Alice in Step C1 while implementing Procedure 2*) *and records the measurement result as* ${e}_{1k}{e}_{2k}$.

#### **Protocol 2 (EKSQPC):**

*Assume that a message* $\mathop{M}\limits_{ \tilde {}}={m}_{1}{m}_{2}\cdots {m}_{s}$ *of length s is sent to Bob by Alice*, *and extra r bits are added to detect* MRA. *Then the protocol functions as follows*:

1. *Alice runs Procedure 2 for* $s+r$ *times and records the values of* ${i}_{k}$ *in string* $\mathop{I}\limits_{ \tilde {}}={i}_{1}{i}_{2}\cdots {i}_{s+r}$.
2. *Bob randomly selects* $s$ *qubits (data bits) from the* $s+r$ *qubits that Alice sends to implement Procedure 3*. *Regarding the residual* $r$ *qubits (probing bits)*, *he executes Procedure 4*. *All the measurement results* ${u}_{k}$ *from Procedure 3 are recorded in a new string* $\mathop{U}\limits_{ \tilde {}}={u}_{1}{u}_{2}\cdots {u}_{s}$ *(after reindexing but preserving the order)*.
3. *Alice performs Procedure 5 on the* $s+r$ *qubits that Bob sends back*, *and records the measurement results* ${e}_{1k}{e}_{2k}$ *in two strings* ${\mathop{E}\limits_{ \tilde {}}}_{1}={e}_{11}{e}_{12}\cdots {e}_{1(s+r)}$ *and* ${\mathop{E}\limits_{ \tilde {}}}_{2}={e}_{21}{e}_{22}\cdots {e}_{2(s+r)}$, *respectively*.
4. * Bob sends a binary string* $\mathop{P}\limits_{ \tilde {}}={p}_{1}{p}_{2}\cdots {p}_{s+r}$ *to Alice through the PBACC to inform her about which qubits were reflected or measured in Step C2*. *For* $k=1,2,\cdots ,s+r$, ${p}_{k}=0$ *indicates that Bob reflected the* ${k}^{th}$ *qubits*, *and* ${p}_{k}=0$ *represents he measured it*.
5. *Alice iterates through* $\mathop{P}\limits_{ \tilde {}}$ *sent by Bob*. *For* $k=1,2,\ldots ,s+r$, *when* ${p}_{k}=0$, *Alice applies function* $AE({e}_{1k},{e}_{2k},{i}_{k})$ *in Definition 2*. *If* $AE({e}_{1k},{e}_{2k},{i}_{k})=1$, *then the* ${k}^{th}$ *qubits sent by Alice is attacked by Eve* (*MRA*). *Then the protocol is insecure and terminated*. *While if* ${p}_{k}=1$, *Alice evaluates function TF* $({e}_{1k},{e}_{2k},{i}_{k})$ *in Definition 3 and records the value* ${c}_{k}$. *Remark that*, *before reindexing*, ${c}_{k}$ *coincides with* ${u}_{k}$ *in Step C2*.
6. * Since s qubits are measured by Bob*, *Alice applies function TF s times in Step C5*. *She records the values of* ${c}_{k}$ *in* $\mathop{C}\limits_{ \tilde {}}={c}_{1}{c}_{2}\cdots {c}_{s}$ (*after reindexing without altering the order*). *Note that* $\mathop{C}\limits_{ \tilde {}}$ *coincides with* $\mathop{U}\limits_{ \tilde {}}$ *that is owned by Bob*.
7. * Alice and Bob execute Procedure 1 with patemeters* ${m}_{k}$, ${c}_{k}$ *and* ${u}_{k}$ ($k=1,2,\ldots ,s$). *Then Bob receives the message sent by Alice*.

Remarkably, Alice and Bob implement Steps C1 to C3 in parallel rather than sequentially. As a result, Alice is only required to be equipped with a small and fixed number of quregisters. Also, the time for Alice to keep the entanglement is a small constant irrelevant to the message length (we elaborate on this highlight in Section 5.1). The protocol essentially distributes a random string of length *m* between Alice and Bob, which implies that our protocol is also a SQKD protocol. After sharing a binary string, Bob can receive messages from Alice by implementing Procedure 1. To mitigate the cost of sharing keys, Step C7 can be repeated for several message transmissions before adopting a new shared string $\mathop{U}\limits_{ \tilde {}}$ $(\,=\mathop{C}\limits_{ \sim })$ (by repeating Steps C1 to C6).

**Figure 2:**
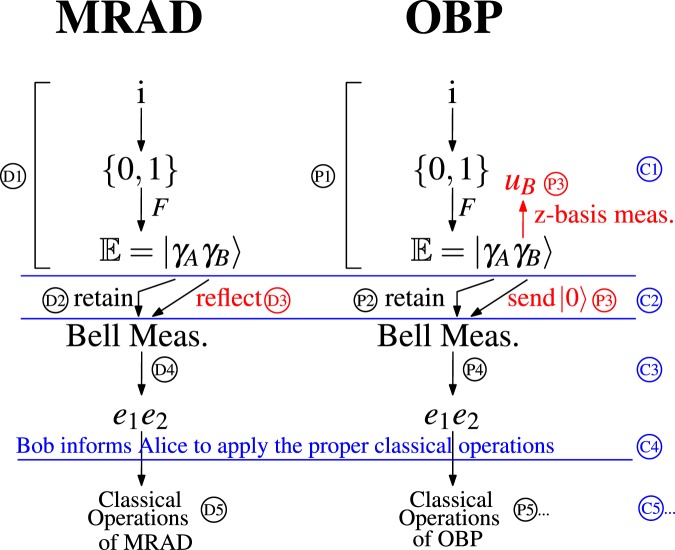
The relationships between EKSQPC, OBP and MRAD. The actions in red are made by Bob and those in Black are performed by Alice. The step numbers in blue are the corresponding actions in EKSQPC. In OBP and MRAD, quantum operations (first four steps) are quite similar except Bob’s treatment on the qubits sent by Alice.

The EKSQPC protocol is an integration of OBP and MRAD. Fig. 2 demonstrates that, in the first four steps, the operations belonging to Alice coincide. Notice that these four steps include all the operations of OBP and MRAD that require quantum resources. As a result, without knowledge of the protocol she is in fact executing, Alice can use one quantum circuit to accomplish all the quantum operations required by either of the protocols.

In Fig. 2, we juxtapose the first four steps of OBP and MRAD marked with the step numbers used to present them. On the right, the step numbers used in the EKSQPC protocol are also provided.

From Fig. 2, only the operation made by Bob differentiates OBP from MRAD. Namely, Bob decides which protocol is being implemented. Specifically, to decide the protocol being applied, Bob either measures *γ*_*kB*_ (and send a pre-prepared $|0\rangle$ simultaneously) or reflects it. Note that the pre-preparation of the $|0\rangle$, instead of generating it on demand, secures EKSQPC against the delay and reflection attacks^11^. A reflected *γ*_*kB*_ functions as a probing bit to detect MRA (then Alice and Bob implement MRAD), and a measured *γ*_*kB*_ works as  a data bit for data exchange (then Alice and Bob implement OBP). After completing the first three steps of EKSQPC, Bob informs Alice of the qubits reflected or measured by sending a notification through the PBACC. Based on the message, Alice applies the corresponding classical operations to complete MRADs or OBPs.

#### **Remark 4**.

*The protocol being implemented is determined by Bob*. *If Bob chooses Measure*, *then it is OBP*. *If he chooses Reflect*, *then it is MRAD*. *In EKSQPC*, *Bob selects Measure s times and Reflect r times*. *So Alice and Bob execute OBP* *s times and MRAD r times*.

### Security analysis of EKSQPC

The EKSQPC inherits the security of OBP and functions under the same assumption – Alice and Bob must be connected by an authentic classical channel (Remark 3). By Remark 4, EKSQPC with *s* data bits and *r* probing bits is equivalent to *s* OBPS and *r* MRADs. Recall that MRAD is for detecting MRA and thus secures OBP (Theorem 2). When MRADs are performed *n* times, the possibility of detecting MRA is 1 − 0.5^*n*^ (Theorem 1). In particular, since there are *r* times executions of MRADs in the EKSQPC protocol, we have 1 − 0.5^*r*^ success rate of detection given that all qubits sent by Alice are measured by Eve. If we generalize the problem by assuming that Eve perpetrates MRA on the qubits with a fixed probability, we have the theorem as follows.

#### **Theorem 3**.

*Suppose that Alice and Bob implement the EKSQPC protocol with s data bits and r probing bits*. *For each qubits sent by Alice*, *if Eve has probability p to perpetrate MRA*, *then Alice has the probability 1* − (*1* − *p*/*2*)^*r*^*to detect it*.

#### *Proof*.

Let *A* be the number of probing bits attacked by Eve. As Eve has possibility *p* to perpetrate an MRA on each probing bit, *A* follows a binomial distribution having success rate *p* with *r* trials. Let *D* be a boolean Random Variable (r.v.) such that $D=1$ if Alice detects an attack and $D=0$ if not. Then the expectation *E*(*D*) is the probability of detecting an attack, and it satisfies

$$\begin{array}{llll}E[D] & = & 0\cdot Pr[D=\mathrm{0]}+1\cdot Pr[D=\mathrm{1]} & \\  & = & Pr[D=\mathrm{1]} & \\  & = & \sum _{h=0}^{r}Pr[D=\mathrm{1|}A=h]Pr[A=h] & ({\rm{Law}}\,{\rm{of}}\,{\rm{total}}\,{\rm{probability}})\\  & = & \sum _{h\mathrm{=0}}^{r}(1-{0.5}^{h})(\begin{array}{c}r\\ h\end{array}){p}^{h}{\mathrm{(1}-p)}^{r-h} & ({\rm{by}}\,{\rm{Theorem}}\,\mathrm{1)}\\  & = & \sum _{h\mathrm{=0}}^{r}(\begin{array}{c}r\\ h\end{array}){p}^{h}{\mathrm{(1}-p)}^{r-h} & \\  &  & -\,\sum _{h\mathrm{=0}}^{r}{0.5}^{h}(\begin{array}{c}r\\ h\end{array}){p}^{h}{\mathrm{(1}-p)}^{r-h} & \\  & = & 1-{(0.5p+\mathrm{(1}-p))}^{r} & ({\rm{Binomial}}\,{\rm{expansion}})\\  & = & 1-{(1-0.5p)}^{r} & \end{array}$$

$\square$Regarding Theorem 3, if $p=1$, Eve attacks all the qubits that Alice sends. Then the probability of detecting an MRA is $E(D)=1-{(1-0.5)}^{r}=1-{0.5}^{r}$, which is consistent with the discussion at the beginning of Section 3.3.

**Figure 3:**
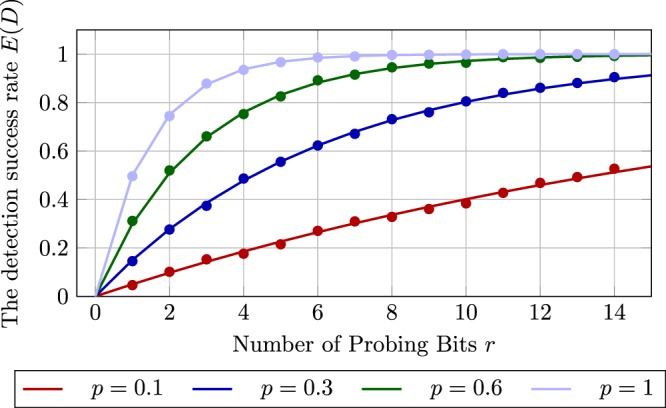
The detection success rate *E*(*D*) with respect to the probing bit number *r* by selecting various attack probability *p*.

 Fig. 3 plots the trend of the detection success rate, calculated according to the formula stated in Theorem 3. We also scatter the experimental results (the points) from simulation. According to what the legend shows, the points and curves colored the same share the same attack probability *p*. The results of the simulation agree with the theoretical analysis in Theorem 3. The figure shows that the detection success rate approaches to one more rapidly as *p* increases. This trend is due to the fact that a higher attack rate leads to a higher average number of affected probing bits and thus boosts the detection success rate. A similar trend can be observed if the probing bit number *r* increases.

The next theorem shows the EKSQPC protocol is secure under the assumption that we can always detect MRA.

#### **Theorem 4**.

*The EKSQPC protocol is secure if MRA can always be detected*.

#### *Proof*.

Alice and Bob terminate the protocol if an MRA is detected. So the security of the message is guaranteed. Otherwise, there is no attack because of the assumption. According to Remark 4, the EKSQPC protocol with *s* data bits performs OBP for *s* times. Combining with Theorem 2, we conclude that the EKSQPC protocol is resistant to any network attack.

#### **Remark 5**.

*It is the prerequisite for Theorem 4 that only one qubit is involved when Alice and Bob send*, *measure or reflect* qubits. *This implies that the implementation of the protocol requires a generator of an individual photon stream which is*, *however*, *currently not available*. *In practice*, *if we use weak laser pulses out of expediency*, *more than one photon may be included*. *This enables Photon Number Splitting (PNS) attacks which cannot be handled by our protocol*. *To avoid the attacks related to PNS*, *readers may refer to Refs*^22–25^. *The same comment also applies to Theorem 7*.

## Rate Estimation EKSQPC (REKSQPC)

EKSQPC detects MRA and is secure assuming no hardware fault nor environmental disturbance that destroy entanglement. So far, we ignored them for the sake of simplicity. They do exist in practice. Ignoring them produces false positives and incorrect protocol terminations. In this section, we enhance the detection part of the protocol to fix this issue. Destructions of entanglement involving probing bits may result in Positive MRADs (PMs) whose probability is denoted by $\rho$ and estimated by its rate

$$\hat{\rho }\,:\,=\frac{{\rm{Number}}\,{\rm{of}}\,{\rm{PMs}}\,}{{\rm{Number}}\,{\rm{of}}\,{\rm{probing}}\,{\rm{bits}}}\mathrm{.}$$

The destructions have two types. In particular, we say that a qubit is disturbed if the entanglement involving it is destroyed due to a hardware imperfection or an environmental disturbance. If the destruction of the entanglement is caused by an eavesdropper Eve, we say the qubit is attacked. We show that two times $\hat{\rho }$ is an estimator $\hat{\kappa }$ of the probability $\kappa$ that a probing bit is disturbed or attacked. Let $\omega$ denote the probability that a qubit is disturbed. If $\omega$ is unknown, we can estimate it ahead of the protocol execution assuming that Eve does not perpetrate attacks. As no qubits are attacked during the estimation, $\kappa$ is reduced to $\omega$. Correspondingly, $\hat{\kappa }$ is reduced to $\hat{\omega }$, an estimator of $\omega$. During the execution of the protocol, the attacks perpetrated by Eve increase $\kappa$ and cause its deviation from $\omega$. By monitoring the difference between $\kappa$ and $\omega$, we gauge the existence of attacks and thus the security of the protocol. We use the following symbols and facts for the statistical analysis in the sequel. Let *B*(*n*, *p*) be a binomial distribution with $n\in {\mathbb{N}}$ trials and success rate $p\in [0,1]$, $N(\mu ,{\sigma }^{2})$ be a normal distribution with mean $\mu \in {\mathbb{R}}$ and variance *σ*^2^ and $\bar{X}$ be the arithmetic mean of *X*.

### **Remark 6**.

*We call B*(1, *p*) *a Bernoulli distribution with the success rate p*.

### **Remark 7**.

*r*.*v*. *of binomial distributions can be added if they have the same success rate*. *In particular*, *if* $X\sim B(n,p)$ *and* $Y\sim B(m,p)$, *then* $X+Y\sim B(n+m,p)$^26^.

### **Fact 1**.

*Suppose* $X\sim N({\mu }_{X},{\sigma }_{X}^{2})$. *Then* $\frac{X-{\mu }_{X}}{{\sigma }_{X}}$ *follows a standard normal distribution*. *Namely*, $\frac{X-{\mu }_{X}}{{\sigma }_{X}}\sim N\mathrm{(0},\mathrm{1)}$.

### **Fact 2**.

*Suppose* $X\sim N({\mu }_{X},{\sigma }_{X}^{2})$ *and* $n\in {{\mathbb{R}}}^{+}$. *Then*, $\frac{X}{n}\sim N(\frac{{\mu }_{X}}{n},\frac{{\sigma }_{X}^{2}}{{n}^{2}})$.

### **Fact 3**.

*Suppose* $X\sim N({\mu }_{X},{\sigma }_{X}^{2})$ *and* $Y\sim N({\mu }_{Y},{\sigma }_{Y}^{2})$ *are independent*. *Then*, $X-Y\sim N({\mu }_{X}-{\mu }_{Y},{\sigma }_{X}^{2}+{\sigma }_{Y}^{2})$.

Theorem 5 discusses the random processes in the detection of disturbed and attacked qubits.

### **Theorem 5**.

*Suppose that in the* EKSQPC *protocol*, *Bob reflects* $r$ *qubits*. *Let* ${D}_{i}\in \{0,1\}$ *denote a r*.*v*. *of the detection result* ${d}_{i}$ *of the* ${i}^{th}$ *MRAD such that*:

$${d}_{i}=\{\begin{array}{cc}1 & if\,the\,{i}^{th}\,MRAD\,has\,a\,positive\,detection\,result\\ 0 & otherwise\end{array}$$

*Then D*_*i*_’*s are independent and identically distributed* (*iid*) $B(1,\,\rho )$. *Or in short*, ${D}_{i}\mathop{\sim }\limits^{iid}B\mathrm{(1,}\,\rho )$. *The number of PMs* (*denoted by* ${C}_{\rho }$) *is* ${\sum }_{i=1}^{r}\,\,{D}_{i}$, *which is a binomial distribution* $B(r,\rho )$. *Moreover*, $\rho =\kappa /2$.

### *Proof*.

Since $\rho$ is the probability of positive detection and all MRADs are mutually independent, ${D}_{i}\mathop{\sim }\limits^{iid}B(1,\rho )$ for $i=1\cdots r$. Then the number of PMs ${C}_{\rho }={\sum }_{i=1}^{n}{D}_{i}$. By Remark 7, we have ${C}_{\rho }\sim B(r,\rho )$. Let ${A}_{i}$ be a r.v. such that, if the probing bit of ${i}^{th}$ MRAD is disturbed or attacked, then ${A}_{i}=1$; otherwise, ${A}_{i}=0$. So we have, $Pr[{A}_{i}=\mathrm{0]}=1-\kappa$ and $Pr[{A}_{i}=1]=\kappa$. According to Lemma 1, if the probing bit is disturbed or attacked, the probability of a positive detection is $Pr[{D}_{i}=\mathrm{1|}{A}_{i}=\mathrm{1]}=\frac{1}{2}$. Otherwise, the probing bit is intact which implies that the result must be negative. Namely, $Pr[{D}_{i}=\mathrm{1|}{A}_{i}=\mathrm{0]}=0$. By Law of total probability,

$$\begin{array}{rcl}\rho  & = & Pr[{D}_{i}=\mathrm{1]}=Pr[{D}_{i}=\mathrm{1|}{A}_{i}=\mathrm{1]}\\  &  & \cdot \,\,Pr[{A}_{i}=\mathrm{1]}+Pr[{D}_{i}=\mathrm{1|}{A}_{i}=\mathrm{0]}\\  &  & \cdot \,\,Pr[{A}_{i}=\mathrm{0]}=\frac{1}{2}\cdot \kappa +0\cdot \mathrm{(1}-\kappa )=\frac{\kappa }{2}.\end{array}$$

$$\square $$

### **Remark 8**.

*A binomial distribution* $B(n,p)$ *has mean np and variance* $np(1-p)$. *So the binomial distribution* ${C}_{\rho }\sim B(r,\rho )$ *in Theorem 5 has mean* $r\rho =\frac{\kappa }{2}r$ *and variance* $r\rho (1-\rho )=\frac{1}{2}r\kappa (1-\frac{\kappa }{2})$^26^.

### **Remark 9**.

*A binomial distribution* $B(n,p)$ *can be approximated by a normal distribution with the same mean and variance if* $n\ge max\{\frac{45{(1-2p)}^{2}}{p(1-p)},\frac{14|1-6p(1-p)|}{p(1-p)}\}$^27^. *Therefore*, *the binomial distribution* ${C}_{\rho }\sim B(r,\frac{\kappa }{2})$ *in Theorem 5 has a normal approximation* $N(\frac{\kappa }{2}r,\frac{1}{2}\kappa r(1-\frac{\kappa }{2}))$ *if* $r\ge max\{\frac{180{(1-\kappa )}^{2}}{p(2-\kappa )},\frac{56|1-3\kappa (1-0.5\kappa )|}{\kappa (2-\kappa )}\}$.

Theorem 6 provides a method to estimate the parameter *p* of a Bernoulli distribution^26^.

### **Theorem 6**.

*Suppose that* ${X}_{i}\mathop{\sim }\limits^{iid}B(1,p)$ *for* $i\in \{1,2,\ldots ,n\}$. *Then* $\hat{p}=\bar{X}=\frac{{{\rm{\Sigma }}}_{i=1}^{n}{x}_{i}}{n}$, is *an unbiased estimator of p*.

By Theorem 6, $\rho =\frac{\kappa }{2}$ has an unbiased estimator $\hat{\rho }=\widehat{\kappa /2}=\frac{{\sum }_{i=1}^{r}{D}_{i}}{r}=\frac{{C}_{\rho }}{r}$. Therefore, $\kappa$ can be estimated by

1 $$\hat{\kappa }=\frac{2{C}_{\rho }}{r}.$$

Remark 9 states that ${C}_{\rho }={\sum }_{i\mathrm{=1}}^{r}\,{D}_{i}\sim B(r,\frac{\kappa }{2})$ approximately follows the normal distribution $N(\frac{\kappa r}{2},\frac{1}{2}\kappa r(1-\frac{\kappa }{2}))$. Combining with Fact 2, we conclude that $\widehat{\kappa /2}=\frac{{C}_{\rho }}{r}\sim N(\frac{\kappa }{2},\frac{\kappa (1-\frac{\kappa }{2})}{2r})$. Applying Fact 2 again, we have $\widehat{\kappa }\sim N(\kappa ,\frac{2\kappa (1-\frac{\kappa }{2})}{r})$.

### Rate difference monitoring

When $\omega$ is unknown, we need to estimate it before starting the execution of the protocol. We have to assume that during this estimation, there is no attack. Under this assumption, $\kappa$ is reduced to $\omega$, the probability that a probing bit is disturbed. Correspondingly, $\hat{\kappa }$ is reduced to an estimator of $\omega$. Namely, $\kappa =\omega$ and $\hat{\kappa }=\hat{\omega }$. As we have shown $\widehat{\kappa }\sim N(\kappa ,\frac{2\kappa (1-\frac{\kappa }{2})}{r})$, we also have $\hat{\omega }\sim N(\omega ,\frac{2\omega (1-\frac{\omega }{2})}{s})$, where *s* is the number of probing bits for estimating $\omega$. Let ${C}_{\rho }^{{\rm{^{\prime} }}}$ denote the number of PMs under the assumption that the probing bits are not attacked. By replacing ${C}_{\rho }$ by ${C}_{\rho }^{{\rm{^{\prime} }}}$ and *s* by *r* in Equation (1), we get,

2 $$\hat{\omega }=\frac{2{C}_{\rho }^{{\rm{^{\prime} }}}}{s}.$$

In REKSQPC, the attack detection method is implemented by checking that $\kappa =\omega$. After getting the estimations of $\kappa$ and $\omega$, let *e* denote their difference, which is an outcome of r.v. $E=\hat{\kappa }-\hat{\omega }$. Fact 3 states that *E* still follows a normal distribution. In particular, $E\sim N(\kappa -\omega ,\frac{2\kappa (1-\frac{\kappa }{2})}{r}+\frac{2\omega (1-\frac{\omega }{2})}{s})$. Under the null hypothesis *H*_0_ that there is no attack, $\kappa =\omega$. Then $E\sim N(0,2\nu (1-\frac{1}{2}\nu )(\frac{1}{r}+\frac{1}{s}))$, where $\nu =\kappa =\omega$ and can be estimated by $\hat{\nu }=\frac{2({C}_{\rho }^{{\rm{^{\prime} }}}+{C}_{\rho })}{r+s}$. So, if *H*_0_ is true, the distribution of r.v. *E* is condensed near zero. Although the set of the possible outcomes of *E* is ${\mathbb{R}}$, the test can rule out outcomes that are much greater than zero without introducing much error (note that we do not consider a negative difference because $\kappa$ is, theoretically, not less than $\omega$. In other words, the alternative hypothesis *H*_1_ is $\kappa  > \omega$). Let *α* denote the probability that an outcome of *E* is much greater than zero and ruled out by the test. We can test *H*_0_ against *H*_1_ by rejecting *H*_0_ if we observe an outcome of *E* greater than *e*_*α*_, where ${e}_{\alpha }\in {\mathbb{R}}$ such that $Pr[E > {e}_{\alpha }]=\alpha$. In other words, the protocol is considered insecure if $e$, the difference between the estimations of $\kappa$ and $\omega$, is greater than *e*_*α*_.

The arduous calculation of *e*_*α*_ can be avoided if we scale *E* to

3 $$Z=\frac{E-0}{\sqrt{2\nu (1-\frac{1}{2}\nu )(\frac{1}{r}+\frac{1}{s})}}=\frac{\hat{\kappa }-\hat{\omega }}{\sqrt{2\hat{\nu }(1-\frac{1}{2}\hat{\nu })(\frac{1}{r}+\frac{1}{s})}},$$

a standard normal distribution according to Fact 1. So correspondingly, the difference *e* after scaling (denoted by *z*) is an outcome of *Z*. Then an equivalent test can be made by rejecting *H*_0_ if $z > {z}_{\alpha }$ where ${z}_{\alpha }\in {\mathbb{R}}$ such that $Pr[Z > {z}_{\alpha }]=\alpha$. The table listing the value of *z*_*α*_ as a function of *α* can be found in Reference^26^. Therefore, we amend the original EKSQPC protocol as follows:

#### **Protocol 3 (REKSQPC)**

:

*RC1 (Estimation of* $\omega$*) Alice and Bob execute MRAD s times*. *Alice sends s qubits to Bob*. *He reflects all of them*. *In other words*, *there are s probing bits and zero data bits*. *In Step C5*, *Alice counts the number of PMs (denoted by* ${C}_{\rho }^{{\rm{^{\prime} }}}$*)*. *Finally*, *she uses* Equation (2) *to estimate* $\omega$. *Note that during the estimation process*, *we need to guarantee that Eve does not perpetrate attacks*.

*RC2 Alice and Bob start the execution of the protocol*. *They do Steps C1*–*C4*.

*RC3 In C5*, *instead of terminating the protocol when* ${p}_{k}=0$ *and function* $AE({e}_{1k},{e}_{2k},{i}_{k})=1$, *Alice increments a counter* ${C}_{\rho }$ (*initial value is zero*) *and continues to check the remaining bits of* $\mathop{P}\limits_{ \tilde {}}$. *After finishing checking*, *she uses* Equation (1) *to estimate* $\kappa$. * She tests the null hypothesis* ${H}_{0}\,:\kappa =\omega$ *against the alternative hypothesis* ${H}_{1}\,:\kappa  > \omega$, Equation (3). *If H*_0_*is rejected*, *Alice considers the protocol is insecure and terminates it*; *otherwise*, *Alice and Bob execute Steps C6 and C7 to complete the data transmission*.

When $\omega$ is given,  Alice can simply compare it with the estimation of $\kappa$. Similarly, we need to test ${H}_{0}\,:\kappa =\omega$ against ${H}_{1}\,:\kappa  > \omega$. Since $\omega$ is not estimated but a given constant, we can say $\hat{\omega }\sim N(\omega ,0)$. We estimate the real attack rate $\kappa$ by Equation (1). Applying Fact 3, we have that $E=\hat{\kappa }-\omega =\hat{\kappa }-\hat{\omega }\sim N(\kappa -\omega ,\frac{2\kappa (1-\frac{\kappa }{2})}{r})$. Under the assumption that *H*_0_ is true, $E\sim N(0,\frac{2\kappa (1-\frac{\kappa }{2})}{r})$. Applying Fact 1, we scale *E* to $Z^{\prime} =\tfrac{(\hat{\kappa }-\omega )-0}{\sqrt{\tfrac{2\kappa (1-\tfrac{\kappa }{2})}{r}}}=\tfrac{\hat{\kappa }-\omega }{\sqrt{\tfrac{2\kappa (1-\tfrac{\kappa }{2})}{r}}}\sim N(0,1)$. Let *z*′ denote the scaled difference of the estimated $\kappa$ and the pre-known $\omega$, which is an outcome of *Z*′. We reject *H*_0_ if *z*′ > *z*_*α*_, where the definition of *z*_*α*_ is unchanged.

Since $\omega$ is given, its estimation is unnecessary. To complete the data transmission, Alice and Bob only need to implement Steps RC2 and RC3, where *Z* is replaced by *Z*′.

### Security analysis of REKSQPC

As we have mentioned at the beginning of this section, the original MRAD fails if the qubits transmitted are disturbed and the entanglement is destroyed. The false positives mislead the protocol about the transmission security and cause wrong termination. To fix the problem, in Sections 4.1, we propose a new detection method for MRA based on a statistical test. The method detects the discrepancy between $\omega$ and $\kappa$, which does not exist if there is no attack. If any significant discrepancy is identified, the protocol is considered insecure and terminated.

The test rules out the possible outcomes of *E* that are largely greater than zero, and thus, introduces detection errors. In more details, suppose that Eve does not perpetrate attacks, which implies $\kappa =\omega$ and the null hypothesis *H*_0_ is true. Due to the fluctuation of the estimator *D*, the difference between $\hat{\kappa }$ and $\hat{\omega }$, there is a proabability *α* that the sampling of *D* is greater than the threshold *d*_*α*_ and gets the *H*_0_ rejected, which is a false positive. Correspondingly, if Eve perpetrates attacks and causes $\kappa  > \omega$, it is also possible that *H*_0_ is not rejected since their difference is still less than *d*_*α*_, which is a false negative. We formally define these two types of errors as follows,

#### **Definition 4**

(Type A Error - False Negative). *Eve perpetrates an attack*, *but the protocol is wrongly considered secure*.

#### **Definition 5**

(Type B Error - False Positive). *Eve does not perpetrate an attack*, *but the protocol is wrongly considered insecure*.

The Type A Error has more adverse consequences than the Type B Error because Eve can eavesdrop the message without the awareness of Alice and Bob. We show that the probability of undetected eavesdropping is very low, even when a small number of probing bits is used. The Type B error does not undermine the security of the protocol. Instead, it lowers the transmission efficiency. While Eve does not perpetrate an attack, the Type B error causes a wrong belief of its presence and a termination of the protocol. The protocol needs to restart and resend all qubits. The transmission efficiency is affected.

The choice of a specific value for *α*, the occurrence probability of the Type B Error, affects the one of the Type A Error. In particular, an increase of *α* pushes the value of *d*_*α*_ to zero. Although Eve only attacks a few portion of the probing bits, the difference between $\kappa$ and $\omega$ she introduces may still exceed the lowered *d*_*α*_ and get *H*_0_ rejected; therefore, the test becomes stricter and the occurrence probability of Type A Error decreases. Similarly, we can show that a decrease of *α* leads to an increase of Type A Error occurrence probability. Since the Type A and B Error occurrence probabilities have a negative relationship, if we increase *α* to enhance the security level, we get more Type B Errors and lower transmission efficiency. Conversely, to decrease the overhead, security is undermined.

**Figure 4:**
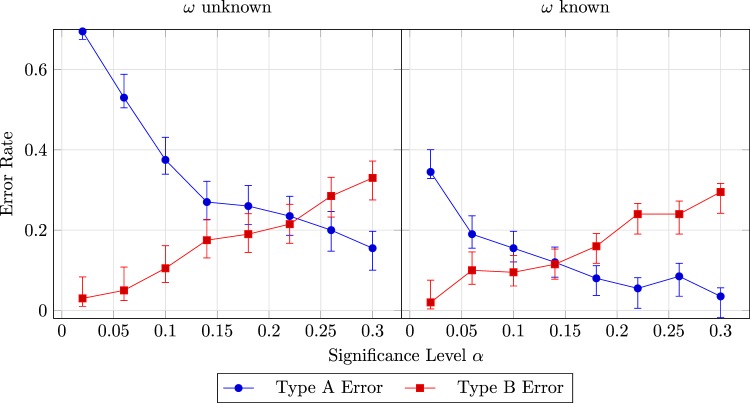
Error rates and their 95% confidence intervals as a function of significance level when the probability $\omega$ that a qubits is disturbed is unknown (left) and known (right). (Simulation configuration: $r=600$, $s=600$ (if $\omega$ is unknown), $\omega =0.3$, $p=0.1$).

With the results of simulations, Fig. 4 plots the rates of the two types of errors as a function of *α*. For estimating the Type A Error occurrence probability, we set the probability *p* for Eve to attack a qubit to 10% for both cases, and the number of probing bits to estimate $\kappa$ and $\omega$ (if unknown) to 600. Note that the configuration here is intended to make the Type A Error occurrence probability more sensitive to the choice of alpha, which is not typical in practical problems. We will talk about how the error occurrence probabilities behave with more common configurations in the sequel. Whether the value $\omega$ is known or not, the trends for both types are consistent with our analysis. When $\omega$ is given, the probability of the Type A Error is lower because the estimation of $\omega$ introduces more variance, which further amplifies the fluctuation of the estimation of the difference $D=\kappa -\omega$. Regarding the Type B Error, we can observe that the rate roughly equals *α* which makes sense since it is an estimation of it.

**Figure 5:**
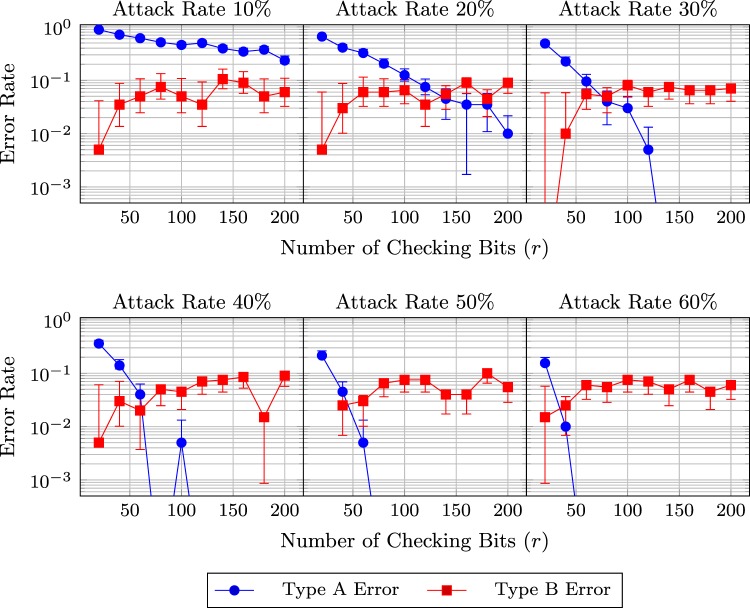
Error rates and their 95% confidence intervals with respect to the number of probing bits when Eve has probability $p=0.1$, 0.2, …, 0.6 to attack a qubit. (The probability $\omega$ that a qubit is disturbed is unknown. Simulation configuration: $\omega =0.05$, $\alpha =0.05$).

**Figure 6:**
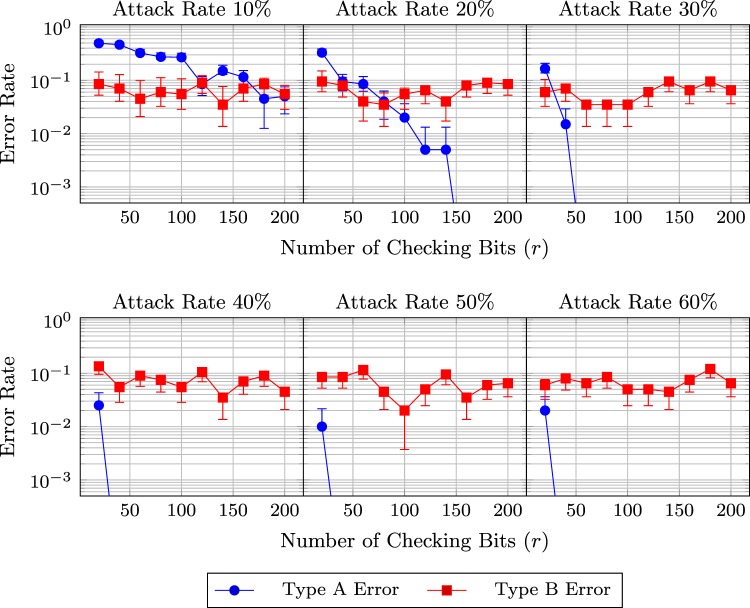
Error rates and their 95% confidence intervals with respect to the number of probing bits when Eve has probability $p=0.1$, 0.2, …, 0.6 to attack a qubit. (The probability $\omega$ that a qubit is disturbed is known. Simulation configuration: $\omega =0.05$, $\alpha =0.05$).

Besides the occurrence probability *α* of the Type B Error, the numbers of probing bits required to estimate $\omega$ and $\kappa$ are also related to the transmission efficiency. A larger number of probing bits contributes to a better estimation, but also has higher overhead. With the results of simulations, Figs 5 and 6 plot the rates of the Type A and B Errors as a function of the number of probing bits and the attack rate. The probability ($\omega =0.05$) that a qubits is disturbed is unknown in Fig. 5 but pre-known in Fig. 6. *α* is set to 0.05. In Fig. 5, the numbers of probing bits coincide for the estimation of $\omega$ and $\kappa$.

The two figures show that when Eve is more likely to attack a  qubit, the detection success rate increases. If Eve only attacks a small proportion of qubits, her attacks do not significantly increase $\kappa$ and thus are concealed by $\omega$. However, in order to successfully eavesdrop messages, Eve should perpetrates attacks at a rate higher than 50%. When the probability of attacks is 60%, 60 probing bits are sufficient to avoid the Type A Error (when $\omega$ is unknown). If $\omega$ is given, then 40 probing bits can achieve the same security level.

Note that the Type B Error rate should be constant. In particular, its mean is theoretically equal to 5% as it is an estimation of *α*. However, while the estimated rate roughly stays around 5% in Fig. 6, a relatively considerable increase is observed in Fig. 5. The increase is due to a low number of probing bits. According to Remark 9, a good normal approximation requires a large sample size and to estimate both $\omega$ and $\kappa$, a even larger one is needed. Although, the approximation is not quite accurate when the probing bit number is small, a low level of Type A Error rate shows that it is good enough to secure the protocol.

Since the REKSQPC and EKSQPC protocols are the same except for the part that detects MRA, Theorem 4 is also applicable to REKSQPC. In particular, we have Theorem 7.

#### **Theorem 7**.

*With a sufficient number of probing bits*, *the Type A Error can be avoided*. *So the REKSQPC protocol is secure*.

## Quantum Resource Requirements and Transmission Efficiency

In this section, we analyze the requirements of quantum resources, qubit efficiency and quantum circuit complexity of the EKSQPC protocol. Among all SQKD and SQDC protocols, we show that the EKSQPC protocol has the highest qubits efficiency (almost 100%) with the simplest quantum circuits (without qubits permutation and measurement basis switch). Comparing to the protocols utilizing the quantum entanglements, we show that the EKSQPC protocol reaches the theoretical minimum of the quregister size and the EPT among the SQKD and SQDC protocols.

### Quantum resources requirements

We briefly discuss the quantum resources requirements at the end of Section 3.2. In this section, we elaborate them. Since the revised version introduced in Section 4 does not change the hardware requirements, we discuss them together.

**Figure 7:**
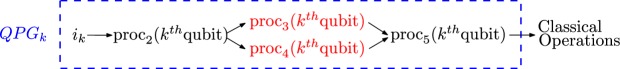
The EKSQPC protocol flow diagram of the procedures requiring quantum resources. *The procedures marked in red belong to Bob*. *For each QPG*_*k*_, *Bob chooses either* $pro{c}_{3}({k}^{th}\,{qubit})$ to measure or $pro{c}_{4}({k}^{th}\,{qubit})$ to reflect.

Alice and Bob loop over *i*_*k*_ in *I* to accomplish all the operations requiring quantum resources. Hence, the lowest quantum resources requirements of the protocol implementation agrees with the one to execute a single quantum procedure group *QPG*_*k*_ (plotted in Fig. 7).

In order to generate EPR pairs in Procedure 2, Alice is required to have an EPR pair generation circuit. Besides, a one-bit quregister is needed to retain the first qubit of the pair. Alice needs a Bell measurement circuit to perform Procedure 5. The entanglement of the EPR pair must be preserved until Alice applies a Bell measurement on it (this case happens when the second qubits *γ*_*B*_ is reflected by Bob. Otherwise, Bob measures it, and the preservation time is shorter). Let *C* denote the time that Alice generates, sends and receives the qubits, and *T* the one-way time for the qubits to move between Alice and Bob. Then the EPT is *C* + 2*T* if we do not count the qubits reflection time of Bob. Compared to Alice, the quantum capability of Bob is fundamental. In particular, he should be able to either measure a qubit in the Z-basis, followed by sending a pre-generated $|0\rangle$, or reflect it. Overall, Alice and Bob need the following minimum quantum capabilities for the protocol implementation.

**Alice:** a one-bit quregister, the circuits for Bell measurement and EPR pair generation.

**Bob:** a device that either reflects a qubit or uses the Z-basis to measure it followed by sending a $|0\rangle$.

Among SQDC and SQKD protocols that utilize entanglements, Alice must create at least a pair of entangled qubits and send at least one of the qubits to Bob. Therefore, for containing a qubit, a one-bit quregister is necessary. For checking the potential attacks, Alice must do some quantum operations on the qubit pair consisting of the qubit she retained and the one reflected by Bob. So, the EPT is at least *C* + 2*T*. As the EKSQPC reaches the theoretical lower bound, we conclude that,

#### **Theorem 8**.

*Among SQDC and SQKD protocols that utilize entanglements*, *the EKSQPC protocol only requires theoretically minimal quregister size and EPT*.

### Transmission efficiency

Suppose the string $\mathop{C}\limits_{ \tilde {}}$ $(\,=\,\mathop{U}\limits_{ \tilde {}})$ shared by Alice and Bob is updated for each dialogue. In other words, prior to applying Step C6 and Step C7 to transmit data, Alice and Bob always execute Steps C1 to C5 to share a random binary string.

Consider the original EKSQPC protocol. Suppose that an *s*-bit message is sent to Bob from Alice, and for each qubit Eve has probability *p* to perpetrate an MRA. Then, for a high eavesdropping efficiency, Eve has to choose a *p* close to one. Assume $p=0.6$ and the number of probing bits is 15. Theorem 3 shows that the success rate of detection is higher than 0.995. Moreover, adding a few more probing bits can enhance the security level significantly. In practice, the message length *s* should considerably exceed 15. Then the probing bits can only introduce a negligible overhead. So for sending a message of length *s*, Alice sends roughly *s* qubits to Bob. Thus, the qubit efficiency approaches 100%.

If we consider the possibility that a qubit is disturbed during the qubit transmission, we need to apply the rate estimation version of the protocol. If $\omega$ is unknown, we need around 60 probing bits to reach 99% detection success rate (assuming that $\alpha =0.01$, $\omega =0.05$), which is acceptable considering a much larger total number of qubits transmitted. If the rate is given, then the probing bit number can decrease to 30 (extra 20 probing bits can improve the success rate to almost 100%). Then the overhead from the probing bits is negligible. The major part of the overhead is from *α*, the probability to get Type B Error, which causes a full restart of the protocol. In average, $\alpha \cdot 100 \%$ qubits transmitted are discarded due to the wrong conclusion that the protocol is insecure. If we choose *α* = 0.01 (which is big enough to secure the protocol), the overhead is only 1%.

Notice that Alice is only required to perform Bell measurements. So she only needs a fixed circuit without measurement basis switch capability. Additionally, the operations related to one qubit is irrelevant to those concerned with the others (since the message security does not depend on the bit permutation by Alice and Bob). Therefore, if the transmission or measurement of a single qubit fails, Alice and Bob only need to re-implement the operations associated with that qubit. This enhances the success rate and efficiency of the data transmission potentially.

In Table 3, we make a detailed comparison with other typical SQKD and SQDC protocols. Note that the qubit efficiency ($\eta$) is calculated by

$$\eta =\frac{{\rm{Length}}\,{\rm{of}}\,{\rm{the}}\,{\rm{message}}}{{\rm{Number}}\,{\rm{of}}\,{\rm{qubits}}\,{\rm{sent}}\,{\rm{by}}\,{\rm{Alice}}}.$$

**Table 3:** Comparisons among typical SQKD and SQDC protocols.

Protocols (The protocols using entanglements are marked by *)	Qubit permutation	Basis Switch	Qubit Efficiency *η*	Minimum^#^ quregisters	EPT
Boyer (2009) Randomization-Based SQKD^7^	Yes	Yes	<12.5%	4*n*	0
Boyer (2009) Measure-Resend SQKD^7^	No	Yes	<12.5%	0	0
Zou (2009) Protocol 5^28^	No	Yes	<12.5%	0	0
Wang (2011)^8^ *	Yes	Yes	<50%	6*n*	Worse than linear
Li (2016)^9^	No	Yes	<6.25%	0	0
Luo (2016)^10^ *	Yes	Yes	12.5%	20*n*	Worse than linear
EKSQPC*	No	No	≈100%	1	$C+2T$
REKSQPC*	No	No	≈99%	1	$C+2T$

For the protocols in References^7–9,28^, $\eta$ depends on some parameters other than the length of the message. For these protocols, we give an upper bound for $\eta$. Regarding REKSQPC, $\eta$ is calculated by choosing $\alpha =0.01$. Besides, in the protocol proposed by Li *et al*.^9^, the measurement basis switch is not required of Alice or Bob but delegated to a third full quantum capability computer Charlie.

## Conclusion

In this paper, we proposed a new SQDC protocol (named Economic Keyless Semi-Quantum Point-to-Point Communication). Compared to other SQDC and SQKD protocols, our new protocol has much higher qubit efficiency (almost 100%) and simpler quantum circuits (not requiring switching measurement basis or permuting qubits). While other SQKD and SQDC protocols encrypting messages through entanglements require at least linear EPT and linear size quregister, in our protocol, only Alice is required to have a fixed size (as low as one) quregister and preserve an EPR pair entanglement for time $C+2T$, where *C* is the time that Alice prepares, receives and measures the qubits, and *T* is the one for the qubits to move between Alice and Bob.

Among the protocols using quantum entanglements to encrypt messages, we show that both quregister size and EPT achieve the theoretical minimums. A pre-shared key is not required by our new protocol. Instead, Alice and Bob use the qubits entanglement to share a random string and further use it as a key to secure the data transmission. We used the probing bits to implement MRAD so that the protocol is resistant to MRA.

In our original protocol, Theorem 3 shows that 15 probing bits can lead to a 0.995 success rate of attack detection (given that the adversary Eve has the probability 0.6 of perpetrating an MRA on a single qubit). A few more bits can boost the security level of the protocol significantly (for example, 0.9992 detection success rate can be achieved by using 20 probing bits). If the message size is sufficiently long, then the qubit efficiency can reach almost 100%.

The rate estimation version, the protocol REKSQPC, can function properly and correctly detect attacks perpetrated by Eve while the qubits may be disturbed during the transmission. We designed a test to monitor the difference of $\kappa$, the probability that a qubit is disturbed or attacked, and $\omega$ (estimated or pre-known), the probability that a qubit is disturbed. If the difference is significantly large, the protocol terminates. The simulation results show that 60 probing bits can push detection success rate to almost 100% (assuming that $\alpha =0.05$ and $\omega =0.05$) if $\omega$ is unknown. The number of probing bits can decrease to 40 and achieve the same success rate if $\omega$ is pre-known. Assuming that we can always detect MRA, our protocol is secure against network attacks (Theorems 4 and 7).

## Supplementary information

Similarity reports of the original and revised versions
