# Supplementary material for: Keyless Semi-Quantum Point-to-point Communication Protocol with Low Resource Requirements
Source: Sci Rep. 2019 Jan 11;9:64. doi: 10.1038/s41598-018-37045-0 (PMC6329802; doi:10.1038/s41598-018-37045-0)
Supplement: Supplementary file 1 — Similarity reports of the original and revised versions [file 41598_2018_37045_MOESM1_ESM.pdf]

Revised Version

by Haoye Lu

Submission date: 28-Nov-2018 09:59AM (UTC-0800)
Submission ID: 1045495623
File name: 215270_2_art_file_7011595_pjwzy8.pdf (397.54K)
Word count: 10506
Character count: 49395

Keyless Semi-Quantum Point-to-point Communication Protocol with Low Resource Requirements

Haoye Lu[1,*], Michel Barbeau[2], and Amiya Nayak[1]

[1]University of Ottawa, School of Electrical Engineering and Computer Science (EECS), Ottawa, K1N 6N5, Canada
[2]Carleton University, School of Computer Science, Ottawa, K1S 5B6, Canada
[*]hlu044@uottawa.ca

ABSTRACT

Full quantum capability devices can provide secure communications, but they are challenging to make portable given the current technology. Besides, classical portable devices are unable to construct communication channels resistant to quantum computers. Hence, communication security on portable devices cannot be guaranteed. Semi-Quantum Communication (SQC) attempts to break the quandary by lowering the receiver's required quantum capability so that secure communications can be implemented on a portable device. However, all SQC protocols have low qubit efficiency and complex hardware implementations. The protocols involving quantum entanglement require linear Entanglement Preservation Time (EPT) and linear quregister size. In this paper, we propose two new keyless SQC protocols that address the aforementioned weaknesses. They are named Economic Keyless Semi-Quantum Point-to-point Communication (EKSQPC) and Rate Estimation EKSQPC (REKSQPC). They achieve theoretically constant minimal EPT and quregister size, regardless of message length. We show that the new protocols, with low overhead, can detect Measure and Replay Attacks (MRAs). REKSQDC is tolerant to transmission impairments and environmental perturbations. The protocols are based on a new quantum message transmission operation termed Tele-Fetch. Like QKD, their strength depends on physical principles rather than mathematical complexity.

1 Introduction

Two full quantum capability devices can communicate securely with Quantum Key Distribution (QKD) [1–4]. In this protocol, two communicants have to be armed with advanced quantum components including quantum registers, programmable quantum circuits and quantum generators. Most of them can only function under stable and well-configured environments and occupy large space. So, it is challenging to implement secure communications on portable devices. On the other hand, quantum computers can efficiently break RSA cryptosystem [5], the security foundation of almost all classical communication protocols. Thence, the communication security of portable devices is in imminent danger of collapse.

Semi-Quantum Communication (SQC) intends to break the predicament by limiting the quantum capability of the receiver without dampening the transmission security. The quantum components for realizing limited quantum capability can be designed compact, simple and robust so that they could be integrated into a portable device. The discussions start from two Semi-Quantum Key Distribution (SQKD) protocols reported by Boyer et al. [6, 7]. Compared with QKD, the receiver Bob needs only to perform four quantum operations: (1) generate quantum bits (qubits) in the Z-basis, (2) measure qubits in the Z-basis, (3) permute qubits and (4) access quantum channel. These two new protocols secure the communications by randomizing measurement basis and Bob's treatment on the qubits he receives. For concealing Bob's behaviour, reordering of the qubits is also required. In 2011, Jian et al. [8] proposed a new SQKD protocol that improves qubit efficiency (the message length with respect to the number of qubits sent by Alice) from the original 12.5% to roughly 50% by using entangled qubits. But the Entanglement Preservation Time (EPT) for implementing the protocol is at least linear to the length of the message. So is the quantum bit register (quregister) size. Li et al. [9] showed that Bob's quantum computation task can be delegated to a third party quantum server in semi-quantum communications at the cost of a low qubit efficiency (6.25%). In 2015, Luo and Hwang [10] proposed a new protocol showing that the Public Bidirectional Authentic Classical Channel (PBACC) is unnecessary if the two communicants have a pre-shared key. However, besides a even longer EPT and a low qubit efficiency (12.5%), a larger quregister size is required for each data bit. A similar pre-shared key based protocol proposed by Almousa and Barbeau [11] shows that Bob does not need to store any qubits, but the linear EPT persists. Recently, more work concerning SQC is reported [12–15].

All the aforementioned protocols [6–12] suffer from low qubit efficiency. Most of them have significant large linear quregister size overhead and require permutation of qubits [6–8, 10, 11]. Regarding the protocol involving entangled qubits

[8, 10, 11], the quantum EPT is at least linear. Although a six-hour record has been achieved by Zhong et al. utilizing europium ion implanted in a crystal [16], entanglement time declines considerably should the entangled photons be propagated in an optical fiber (the most common implementation of quantum communication protocols) [17]. Besides, involving permutations on qubits (not practical shortly) dooms to a low transmission efficiency and reliability. Considering that the unusual materials (for instance, coupled electron [18] and ultracold atoms [19]) are necessary for the implementation of quregisters, a commercial quantum network based on them is not feasible in a near future.

This paper reports a new Semi-Quantum Direct Communication (SQDC) protocol and a rate estimation version, named Economic Keyless Semi-Quantum Point-to-Point Communication (EKSQPC) and Rate Estimation EKSQPC (REKSQPC), that address all the aforementioned issues. An innovative operation, called Tele-Fetch (TF), utilizes entangled qubit pairs to transmit messages. It is at the core of the One-Bit Protocol (OBP). The results of measurements on the pairs fall in a predesigned set of values because of the entanglement, but do not carry any useful information. The design makes the OBP functioning without a pre-shared key and fully resistant to information leakage even if the qubits are intercepted. Besides, the protocol uses the same quantum circuit as the one to detect the Measure and Replay Attack (MRA) (called MRA Detection (MRAD)) and thus, not only saves the quantum resources but also becomes the cornerstone of the EKSQPC and REKSQPC protocols. Because Alice performs the same quantum procedures in both protocols (OBP or MRAD), Bob does not need to communicate with Alice until all quantum procedures (Alice's and Bob's) are completed. Alice and Bob execute MRADs using a small portion of the measurement results before using the PBACC to translate the rest into valid messages. The protocol is proved fully secure under the assumption that MRAs are always detectable. As the pivot to secure the messages is a successful detection of MRAs, we show that, with only 15 probing bits, the attack detection success rate can achieve 0.995 (under the assumption that the adversary Eve has 0.6 possibility to attack a qubit). The security of the protocol is enhanced considerably if a few more probing bits are added. The qubit efficiency asymptotically reaches 100% with the message length. The implementation of the EKSQPC protocol has low requirements on quantum resources. In particular, the quregister size required by Alice is as low as one, and the required EPT is $C+2T$ (where C is the time that Alice takes to generate, send and receive the qubits; and T is the one-way time for the qubits to travel between Alice and Bob). We prove that both the quregister size and EPT reach the theoretical minimums.

Considering that the entanglement of qubits may not always persist during the transmission of qubits, we assume that there is a probability ω that the entanglement involving a qubit is destroyed as it can be disturbed by hardware imperfection and environmental disturbance. Under this assumption, we design a statistical test to compare ω with the probability that a qubit is attacked or disturbed. When a significant difference is observed, Alice concludes that Eve perpetrated attacks and aborts the execution of the protocol. Therefore, the communication is not eavesdropped successfully. Compared with the original EKSQPC, more probing bits are required to achieve the same detection success rate; however, the overhead is still low. In particular, our simulation results reveal that 60 probing bits are enough to detect almost all attacks when ω is unknown. If the rate is given, then 40 probing bits are enough to achieve the same detection success rate.

This paper is a revised and extended version of a preliminary workshop paper [20] in which we introduced the original EKSQPC protocol. Compared to the workshop paper, this paper articulates the original protocol as well as its analysis with more details. Based on this, we report an upgraded and practical version, REKSQPC, with its security, resource requirements and transmission efficiency analysis. Moreover, we also provide a detailed comparison with other typical SQKD and SQDC protocols.

The rest of the paper is organized as follows. In Section 2, we review Bell measurement and MRAD, which are integrated in the new protocol. In Sections 3 and 4, we introduce our new protocols including a rate estimation version taking into account the probability that a qubit is disturbed. We also do a security analysis and discuss simulation results. In Section 5, we talk about their quantum resource requirements and transmission overhead. Finally, we draw the conclusions in Section 6.

2 Background

The section starts from a brief review of EPR pairs states and Bell measurement on which our new protocol heavily relies. Then we introduce the MRAs as well as its detection algorithm (MRAD) that secures the data transmission of the new protocol.

In this paper, classical bits (cbits) are denoted by lowercase English letters, and a cbit sequence is represented by an uppercase English letter over a tilde. For instance, $\underset{\sim}{M} = m_1 m_2 \cdots m_t$ is a cbit string of length t. Qubits are denoted by Greek letters and Bell states by Bold English capital letters.

2.1 EPR pairs and Bell measurement

A pair of qubits that are together in Bell state is called an EPR pair. Bell states have four types: $|\Phi^+\rangle = \frac{1}{\sqrt{2}} \cdot (|00\rangle + |11\rangle)$, $|\Phi^-\rangle = \frac{1}{\sqrt{2}} \cdot (|00\rangle - |11\rangle)$, $|\Psi^+\rangle = \frac{1}{\sqrt{2}} \cdot (|01\rangle + |10\rangle)$, and $|\Psi^-\rangle = \frac{1}{\sqrt{2}} \cdot (|01\rangle - |10\rangle)$; we can use the Bell measurement (B.M.) (Figure 1) to identify them. The inputs of the circuit are two qubits γ_A and γ_B, and the outputs are two cbits e_1 and e_2.

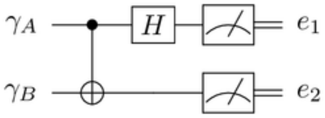

Figure 1. The Bell measurement Circuit.

If $\gamma_A\gamma_B$ is an EPR Pair (Bell state), then the outputs are deterministic and listed in Table 1; otherwise, $\gamma_A\gamma_B$ is mapped into a Bell state stochastically. For $\gamma_A\gamma_B$ equal to $|00\rangle$, $|01\rangle$, $|10\rangle$ or $|11\rangle$, the distributions of the outputs are listed in Table 2.

2.2 Measure and Replay Attack and the method of detection

A simplified version of Man-In-The-Middle Attacks (MITMs) is called replay attack. The attacker Eve deceives the truthful listener(s) by replaying messages outside the expected context so that the listener believes that the protocol has been executed successfully [21].

We can perpetrate a similar attack in the context of quantum communications. Assume that qubits sent or received by Alice and Bob can be intercepted by Eve. As a qubit is sent to Bob by Alice through the quantum channel, Eve uses the Z-basis to measure it. If the measurement result is zero, then Eve sends $|0\rangle$ to Bob; otherwise, she sends $|1\rangle$. As the replay of the message follows the measurement, we call the attack the Measure and Replay Attack (MRA) [11].

The following method demonstrates how to utilize the EPR pairs and Bell measurement to detect MRAs. The Luo and Hwang's protocol [10] and Almousa and Barbeau's protocol [11] also apply similar ideas for the attack detection.

MRA Detection (MRAD):

D1 Alice randomly picks a cbit $i = 0$ or 1, based on which she generates an EPR pair $\mathbb{E}$ (if $i = 0$, $\mathbb{E} = |\Phi^+\rangle$; else, $\mathbb{E} = |\Psi^-\rangle$).

D2 $\mathbb{E}$ consists of two qubits, γ_A and γ_B. Alice keeps γ_A and sends γ_B (named the probing bit) to Bob.

D3 γ_B is reflected by Bob to Alice.

D4 After receiving γ_B', Alice applies Bell measurement (Figure 1) on $\gamma_A\gamma_B'$ to obtain e_1 and e_2.

D5 The combination of e_1 and e_2 indicates the EPR pair that the circuit measured. We consider the protocol secure (denoted by zero) if the measured EPR pair agrees with the one Alice produced in Step D1. If not, an MRA is detected (denoted by one).

Example 1. *Suppose Alice and Bob implement MRAD for MRA detection. Without loss of generality, assume Alice picks $i = 1$ and thus produces a corresponding EPR pair $\mathbb{E} = |\Psi^-\rangle = \frac{1}{\sqrt{2}} \cdot (|01\rangle - |10\rangle) = \gamma_A\gamma_B$. Alice intends to send γ_B to Bob; however, Eve intercepts and measures it and gets the measurement result $r = 0$. Simultaneously, γ_A retained by Alice collapses to $|1\rangle$ due to the entanglement. Eve produces a new qubit ($|0\rangle$) correspondingly and send it to Bob. Bob does nothing but reflects it back to Alice. γ_A and the received $|0\rangle$ are paired together and measured by Alice using the Bell measurement circuit. Notice that γ_A has collapsed to $|1\rangle$. So the qubit pair measured by the circuit is $\gamma_A\gamma_B = |10\rangle$. By Table 2, we have 50 percent possibility to get $e_1e_2 = 01$ (and so deduce that the input is $|\Psi^+\rangle$ by Table 1, a true positive) and to get $e_1e_2 = 11$ (and thus deduce that the input is $|\Psi^-\rangle$, a false negative).*

The example shows that if Alice picks $i = 1$ and the measurement result r of Eve is zero, there is 50 percent possibility for Alice to deduce that the protocol is secure although the attack is perpetrated. By Table 2, we can draw the same conclusion for any choice of i and r. Hence, Lemma 1 and Theorem 1 follow.

Lemma 1. *Provided that Eve attacks the probing bit, there is 0.5 possibility for MRAD to detect an MRA.*

Theorem 1. *If MRAD are repeated n times, we have $1 - 0.5^n$ probability to detect MRAs given that Eve attacks n probing bits.*

Proof. $Pr[\text{detect MRAs}] = 1 - Pr[\text{MRAD fails}]^n = 1 - 0.5^n$ □

Remark 1. *MRAD essentially checks whether the probing bits sent by Alice had been measured by anybody else, but it cannot tell who measured them. It can detect MRAs only because Alice knows that Bob does not measure probing bits. Therefore, if any measurement is detected, it must be due to an attack.*

Because, the operations defined in Steps D1 and D5 are applied again in the sequel, we define them formally as follows,

Definition 1 (Generating corresponding EPR pairs (F), Step D1). *Function F maps a cbit to an EPR pair such that $0 \mapsto |\Phi^+\rangle$, and $1 \mapsto |\Psi^-\rangle$*

Definition 2 (Alice Examines (AE), Step D5). *The function $AE(e_1, e_2, i) : \{0,1\}^3 \to \{0,1\}$ equals zero if $e_1 = e_2 = i$; otherwise, it equals one. Recall that zero and one indicate negative and positive detection results, respectively.*

3 New Protocol

In this section, we propose a new SQDC protocol called Economic Keyless Semi-Quantum Point-to-Point Communication (EKSQPC). We start the discussion with an introduction to a data transmission protocol called OBP, which is a building block of EKSQPC (not self-contained). Assuming that there are no MRAs, we show that OBP is secure (Theorem 2). In the design of OBP, Bell measurement seems redundant. It is intended for sharing the quantum circuit with MRAD (Remark 2). The considerable benefits of this design are discussed in Section 5. To meet the assumption of Theorem 2, we integrate MRAD and OBP to get EKSQPC. If we assume that EKSQPC detects all MRAs, then it is provably secure (Theorem 4).

3.1 One-Bit Protocol (OBP)

Protocol 1 (OBP). *A one-bit message m (zero or one) is sent to Bob by Alice. We need a PBACC as well as a Public Bidirectional Quantum Channel (PBQC). The protocol functions as follows:*

P1 Alice randomly picks a cbit i and generates a corresponding EPR pair $\mathbb{E} = F(i)$ (Definition 1) consisting of two qubits (denoted by γ_A and γ_B).

P2 Alice keeps γ_A and sends γ_B to Bob.

P3 Upon reception, the qubit γ_B is measured by Bob in the Z-basis with the measurement result u_B (simultaneously, γ_A collapses because of the entanglement with γ_B). At the same moment, Bob sends a pre-prepared qubit $\gamma_B^* = |0\rangle$ to Alice and informs her that he has measured γ_B via the PBACC.

P4 Alice pairs γ_A (retained in Step P1) with γ_B^* and performs a Bell measurement on $\gamma_A \gamma_B^* = \gamma_A |0\rangle$ to get e_1 and e_2.

P5 According to Table 2, $e_1 e_2 = 00$ or 10 implies that $\gamma_A = |0\rangle$, and $e_1 e_2 = 01$ or 11 indicates that $\gamma_A = |1\rangle$. Combining γ_A with the EPR pair $\mathbb{E}$ Alice selected (recorded by i) in Step P1, Alice learns the measurement result u_B of Bob in Step P3. In particular, if $i = 0$, then the EPR pair she generated was $|\Phi^+\rangle$. Then $\gamma_A = |0\rangle$ implies $u_B = 0$, and $\gamma_A = |1\rangle$ implies $u_B = 1$. Similarly, if $i = 1$, the EPR pair that Alice generated was $|\Psi^-\rangle$. Then if $\gamma_A = |0\rangle$, $u_B = 1$; else, $u_B = 0$.

P6 Provided that $u_B = m$, Alice informs Bob, via the PBACC, that u_B is the correct value. Otherwise, she informs Bob to take $1 - u_B$.

Remark 2. *The pre-generated qubit $\gamma_B^* = |0\rangle$ in Step P3 is unnecessary to implement OBP. So is the Bell measurement in Step P4. In fact, in Step P3, Bob only needs to notify Alice that he has measured γ_B, and, in Step P4, Alice simply uses the Z-basis to get the value of γ_A. Here, we intendedly implement OBP with redundant operations so that the new protocol (EKSQPC, introduced in Section 3.2) can use a single quantum circuit to implement both the attack detection (MRAD) and data transmission (OBP) protocols. We discuss the design and its benefits in details in Section 3.2, and more performance analysis is conducted in Section 5.*

The actions specified in Steps P5 and P6 are used subsequently. We define them formally as follows. In Step P5, Alice learns r_B held by Bob with no contact. So the function is called Tele-Fetch.

Definition 3 (Tele-Fetch). *With the parameters e_1, e_2 and i (Step P1), function Tele-Fetch $TF(e_1, e_2, i)$ returns the value of r_B (zero or one) based on the rule contained in Step P5.*

Besides, in Step P6, Alice rectifies the measurement result r_B of Bob. As a result, we call the procedure Rectify.

Procedure 1 (Rectify). *Based on the single bit message m and value of u_B (acquired in Step P5), Alice informs Bob to apply the proper operation on u_B by sending either the signal KEEP or FLIP via the PBACC. If KEEP is received, Bob considers u_B as the message Alice sends; if not, he takes $1 - u_B$.*

The next theorem discusses the security of OBP.

Theorem 2. *As long as Bob gets the qubit γ_B sent by Alice without MRA, OBP is secure.*

Proof. By assuming the absence of MRA, we essentially assume that Steps P1 to P3 are secure (only a confirmation is sent by Bob in Step P3). No communication happens in Step P4 and P5. The last step involves a message sent by Alice which is irrelevant to the one-bit message m. So Step P6 is sheltered, too. Thence, to sum up, OBP is secure. □

Remark 3. *An authentic classical channel is the prerequisite for the security of OBP. In the communication of Alice and Bob, it is significant to verify their identities and to ensure that their unencrypted messages are not altered. Namely, they should be resistant to MITMs.*

Theorem 2 shows that only when there is no MRA, OBP is secure. However, OBP has no capability to detect MRAs. Notice that MRAD can detect MRAs and thus can secure the data transmission of OBP by Theorem 2. If we combine OBP and MRAD together, we get the protocol discussed in the following subsection.

3.2 Economic Keyless Semi-Quantum Point-to-Point Communication (EKSQPC)

We implement the protocol EKSQPC over the hardware of OBP. Specifically, there are a PBACC and a PBQC linking Alice and Bob. The following four procedures contain all the activities demanding quantum resources in EKSQPC.

Procedure 2 (Alice sends)**.** *In the kth transmission of Alice, she picks a random cbit i_k and stores it in a classical register. Then she produces an EPR pair $F(i_k)$, keeps the first qubit γ_{kA} and transmits the second qubit γ_{kB} to Bob.*

Procedure 3 (Bob measures)**.** *After receiving the kth qubit from Alice, Bob measures it in the Z-basis and gets the result u_k. At the same moment, a pre-prepared qubit $|0\rangle$ is sent back to Alice. Furthermore, Bob takes the record that he measured the kth qubit.*

Procedure 4 (Bob reflects)**.** *The kth qubit from Alice is reflected back without measurement by Bob. Also, he takes the record that he reflected the kth qubit he received.*

Procedure 5 (Alice measures)**.** *Alice receives the kth qubit γ_{kB}^* and performs Bell measurement on $|\gamma_{kA}\gamma_{kB}^*\rangle$ (γ_{kA} was retained by Alice in Step C1 while implementing Procedure 2) and records the measurement result as $e_{1k}e_{2k}$.*

Protocol 2 (EKSQPC)**.** *Assume that a message $\underset{\sim}{M} = m_1m_2\cdots m_s$ of length s is sent to Bob by Alice, and extra r bits are added to detect MRAs. Then the protocol functions as follows:*

C1 *Alice runs Procedure 2 for $s+r$ times and records the values of i_k in string $\underset{\sim}{I} = i_1i_2\cdots i_{s+r}$.*

C2 *Bob randomly selects s qubits (data bits) from the $s+r$ qubits that Alice sends to implement Procedure 3. Regarding the residual r qubits (probing bits), he executes Procedure 4. All the measurement results u_k from Procedure 3 are recorded in a new string $\underset{\sim}{U} = u_1u_2\cdots u_s$ (after reindexing but preserving the order).*

C3 *Alice performs Procedure 5 on the $s+r$ qubits that Bob sends back, and records the measurement results $e_{1k}e_{2k}$ in two strings $\underset{\sim}{E_1} = e_{11}e_{12}\cdots e_{1(s+r)}$ and $\underset{\sim}{E_2} = e_{21}e_{22}\cdots e_{2(s+r)}$, respectively.*

C4 *Bob sends a binary string $\underset{\sim}{P} = p_1p_2\cdots p_{s+r}$ to Alice through the PBACC to inform her about which qubits were reflected or measured in Step C2. For $k = 1,2,\cdots,s+r$, $p_k = 0$ indicates that Bob reflected the kth qubit, and $p_k = 0$ represents he measured it.*

C5 *Alice iterates through $\underset{\sim}{P}$ sent by Bob. For $k = 1,2,\cdots,s+r$, when $p_k = 0$, Alice applies function $AE(e_{1k}, e_{2k}, i_k)$ in Definition 2. If $AE(e_{1k}, e_{2k}, i_k) = 1$, then the kth qubit sent by Alice is attacked by Eve (MRA). Then the protocol is insecure and terminated. While if $p_k = 1$, Alice evaluates function $TF(e_{1k}, e_{2k}, i_k)$ in Definition 3 and records the value c_k. Remark that, before reindexing, c_k coincides with u_k in Step C2.*

C6 *Since s qubits are measured by Bob, Alice applies function TF s times in Step C5. She records the values of c_k in $\underset{\sim}{C} = c_1c_2\cdots c_s$ (after reindexing without altering the order). Note that $\underset{\sim}{C}$ coincides with $\underset{\sim}{U}$ that is owned by Bob.*

C7 *Alice and Bob execute Procedure 1 with patemeters m_k, c_k and u_k ($k = 1,2,\cdots,s$). Then Bob receives the message sent by Alice.*

Remarkably, Alice and Bob implement Steps C1 to C3 in parallel rather than sequentially. As a result, Alice is only required to be equipped with a small and fixed number of quregisters. Also, the time for Alice to keep the entanglement is a small constant irrelevant to the message length (we elaborate on this highlight in Section 5.1). The protocol essentially distributes a random string of length m between Alice and Bob, which implies that our protocol is also a SQKD protocol. After sharing a binary string, Bob can receive messages from Alice by implementing Procedure 1. To mitigate the cost of sharing keys, Step C7 can be repeated for several message transmissions before adopting a new shared string $\underset{\sim}{U}$ $(= \underset{\sim}{C})$ (by repeating Steps C1 to C6).

The EKSQPC protocol is an integration of OBP and MRAD. Figure 2 demonstrates that, in the first four steps, the operations belonging to Alice coincide. Notice that these four steps include all the operations of OBP and MRAD that require quantum

resources. As a result, without knowledge of the protocol she is in fact executing, Alice can use one quantum circuit to accomplish all the quantum operations required by either of the protocols.

In Figure 2, we juxtapose the first four steps of OBP and MRAD marked with the step numbers used to present them. On the right, the step numbers used in the EKSQPC protocol are also provided.

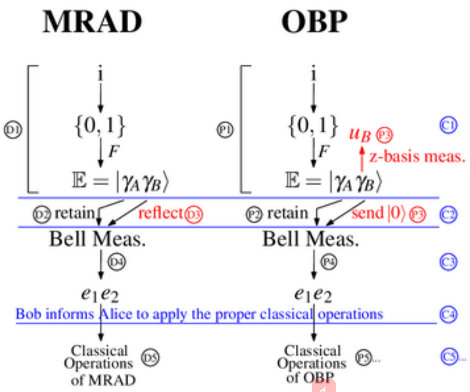

Figure 2. The relationships between EKSQPC, OBP and MRAD. The actions in red are made by Bob and those in Black are performed by Alice. The step numbers in blue are the corresponding actions in EKSQPC. In OBP and MRAD, quantum operations (first four steps) are quite similar except Bob's treatment on the qubits sent by Alice.

From Figure 2, only the operation made by Bob differentiates OBP from MRAD. Namely, Bob decides which protocol is being implemented. Specifically, to decide the protocol being applied, Bob either measures γ_{kB} (and send a pre-prepared $|0\rangle$ simultaneously) or reflects it. Note that the pre-preparation of the $|0\rangle$, instead of generating it on demand, secures EKSQPC against the delay and reflection attacks [11]. A reflected γ_{kB} functions as a probing bit to detect MRAs (then Alice and Bob implement MRAD), and a measured γ_{kB} works a data bit for data exchange (then Alice and Bob implement OBP). After completing the first three steps of EKSQPC, Bob informs Alice of the qubits reflected or measured by sending a notification through the PBACC. Based on the message, Alice applies the corresponding classical operations to complete MRADs or OBPs.

Remark 4. *The protocol being implemented is determined by Bob. If Bob chooses Measure, then it is OBP. If he chooses Reflect, then it is MRAD. In EKSQPC, Bob selects Measure s times and Reflect r times. So Alice and Bob execute OBP s times and MRAD r times.*

3.3 Security analysis of EKSQPC

The EKSQPC inherits the security of OBP and functions under the same assumption – Alice and Bob must be connected by an authentic classical channel (Remark 3). By Remark 4, EKSQPC with s data bits and r probing bits is equivalent to s OBPs and r MRADs. Recall that MRAD is for detecting MRAs and thus secures OBP (Theorem 2). When MRADs are performed n times, the possibility of detecting MRAs is $1-0.5^n$ (Theorem 1). In particular, since there are r times executions of MRADs in the EKSQPC protocol, we have $1-0.5^r$ success rate of detection given that all qubits sent by Alice are measured by Eve. If we generalize the problem by assuming that Eve perpetrates MRAs on the qubits with a fixed probability, we have the theorem as follows.

Theorem 3. *Suppose that Alice and Bob implement the EKSQPC protocol with s data bits and r probing bits. For each qubit sent by Alice, if Eve has probability p to perpetrate MRAs, then Alice has the probability $1-(1-p/2)^r$ to detect it.*

Proof. Let A be the number of probing bits attacked by Eve. As Eve has possibility p to perpetrate an MRA on each probing bit, A follows a binomial distribution having success rate p with r trials. Let D be a boolean Random Variable (r.v.) such that $D=1$ if Alice detects an attack and $D=0$ if not. Then the expectation $E(D)$ is the probability of detecting an attack, and it satisfies

$$E[D] = 0\cdot Pr[D=0] + 1\cdot Pr[D=1] = Pr[D=1] = \sum_{h=0}^{r} Pr[D=1|A=h]Pr[A=h] \quad \text{(Law of total probability)}$$

$$= \sum_{h=0}^{r}\left(1-0.5^h\right)\binom{r}{h}p^h(1-p)^{r-h} \quad \text{(by Theorem 1)}$$

$$= \sum_{h=0}^{r}\binom{r}{h}p^h(1-p)^{r-h} - \sum_{h=0}^{r}0.5^h\binom{r}{h}p^h(1-p)^{r-h} = 1-(0.5p+(1-p))^r \quad \text{(Binomial expansion)}$$

$$= 1-(1-0.5p)^r$$

□

Regarding Theorem 3, if $p = 1$, Eve attacks all the qubits that Alice sends. Then the probability of detecting an MRA is $E(D) = 1-(1-0.5)^r = 1-0.5^r$, which is consistent with the discussion at the beginning of Section 3.3.

Figure 3 plots the trend of the detection success rate, calculated according to the formula stated in Theorem 3. We also scatter the experimental results (the points) from simulation. According to what the legend shows, the points and curves colored the same share the same attack probability p. The results of the simulation agree with the theoretical analysis in Theorem 3. The figure shows that the detection success rate approaches to one more rapidly as p increases. This trend is due to the fact that a higher attack rate leads to a higher average number of affected probing bits and thus boosts the detection success rate. A similar trend can be observed if the probing bit number r increases.

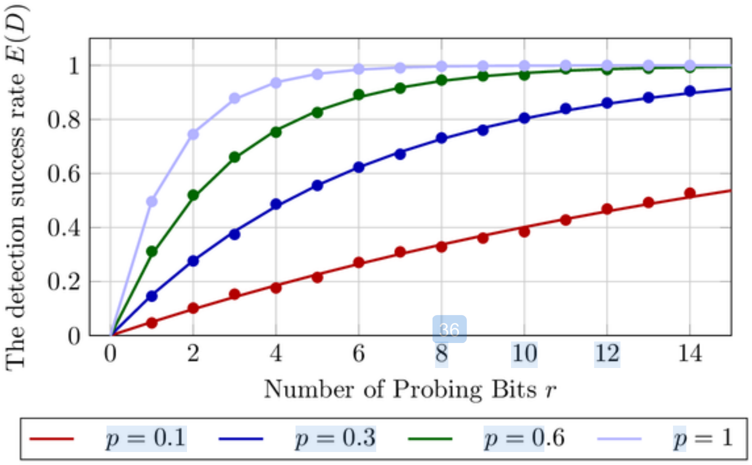

Figure 3. The detection success rate $E(D)$ with respect to the probing bit number r by selecting various attack probability p.

The next theorem shows the EKSQPC protocol is secure under the assumption that we can always detect MRAs.

Theorem 4. *The EKSQPC protocol is secure if MRAs can always be detected.*

Proof. Alice and Bob terminate the protocol if an MRA is detected. So the security of the message is guaranteed. Otherwise, there is no attack because of the assumption. According to Remark 4, the EKSQPC protocol with s data bits performs OBP for s times. Combining with Theorem 2, we conclude that the EKSQPC protocol is resistant to any network attack. □

Remark 5. *It is the prerequisite for Theorem 4 that only one qubit is involved when Alice and Bob send, measure or reflect qubits. This implies that the implementation of the protocol requires a generator of an individual photon stream which is, however, currently not available. In practice, if we use weak laser pulses out of expediency, more than one photon may be included. This enables Photon Number Splitting (PNS) attacks which cannot be handled by our protocol. To avoid the attacks related to PNS, readers may refer to [22–25]. The same comment also applies to Theorem 7.*

4 Rate Estimation EKSQPC (REKSQPC)

EKSQPC detects MRAs and is secure assuming no hardware fault nor environmental disturbance that destroy entanglement. So far, we ignored them for the sake of simplicity. They do exist in practice. Ignoring them produces false positives and incorrect protocol terminations. In this section, we enhance the detection part of the protocol to fix this issue. Destructions of entanglement involving probing bits may result in Positive MRADs (PMs) whose probability is denoted by ρ and estimated by its rate

$$\hat{\rho} := \frac{\text{Number of PMs}}{\text{Number of probing bits}}.$$

The destructions have two types. In particular, we say that a qubit is disturbed if the entanglement involving it is destroyed due to a hardware imperfection or an environmental disturbance. If the destruction of the entanglement is caused by an eavesdropper Eve, we say the qubit is attacked. We show that two times $\hat{\rho}$ is an estimator $\hat{\kappa}$ of the probability κ that a probing bit is disturbed or attacked. Let ω denote the probability that a qubit is disturbed. If ω is unknown, we can estimate it ahead of the protocol execution assuming that Eve does not perpetrate attacks. As no qubits are attacked during the estimation, κ is reduced to ω.

Correspondingly, $\hat{\kappa}$ is reduced to $\hat{\omega}$, an estimator of ω. During the execution of the protocol, the attacks perpetrated by Eve increase κ and cause its deviation from ω. By monitoring the difference between κ and ω, we gauge the existence of attacks and thus the security of the protocol. We use the following symbols and facts for the statistical analysis in the sequel. Let $B(n,p)$ be a binomial distribution with $n \in \mathbb{N}$ trials and success rate $p \in [0,1]$, $N(\mu,\sigma^2)$ a normal distribution with mean $\mu \in \mathbb{R}$ and variance σ^2 and $\bar{X}$ the arithmetic mean of X.

Remark 6. *We call* $B(1,p)$ *a Bernoulli distribution with the success rate* p.

Remark 7. *r.v. of binomial distributions can be added if they have the same success rate. In particular, if* $X \sim B(n,p)$ *and* $Y \sim B(m,p)$, *then* $X+Y \sim B(n+m,p)$ *[26].*

Fact 1. *Suppose* $X \sim N(\mu_X, \sigma_X^2)$. *Then* $\frac{X-\mu_X}{\sigma_X}$ *follows a standard normal distribution. Namely,* $\frac{X-\mu_X}{\sigma_X} \sim N(0,1)$.

Fact 2. *Suppose* $X \sim N(\mu_X, \sigma_X^2)$ *and* $n \in \mathbb{R}^+$. *Then,* $\frac{X}{n} \sim N(\frac{\mu_X}{n}, \frac{\sigma_X^2}{n^2})$.

Fact 3. *Suppose* $X \sim N(\mu_X, \sigma_X^2)$ *and* $Y \sim N(\mu_Y, \sigma_Y^2)$ *are independent. Then,* $X - Y \sim N(\mu_X - \mu_Y, \sigma_X^2 + \sigma_Y^2)$.

Theorem 5 discusses the random processes in the detection of disturbed and attacked qubits.

Theorem 5. *Suppose that in the EKSQPC protocol, Bob reflects r qubits. Let* $D_i \in \{0,1\}$ *denote a r.v. of the detection result* d_i *of the* i^{th} *MRAD such that:*

$$d_i = \begin{cases} 1 & \text{if the } i^{th} \text{ MRAD has a positive detections} \\ 0 & \text{otherwise} \end{cases}$$

Then D_i*'s are independent and identically distributed (iid)* $B(1,\rho)$. *Or in short,* $D_i \overset{iid}{\sim} B(1,\rho)$. *The number of PMs (denoted by* C_ρ*) is* $\sum_{i=1}^{r} D_i$, *which is a binomial distribution* $B(r,\rho)$. *Moreover,* $\rho = \frac{\kappa}{2}$.

Proof. Since ρ is the probability of positive detection and all MRADs are mutually independent, $D_i \overset{iid}{\sim} B(1,\rho)$ for $i = 1 \cdots r$. Then the number of PMs $C_\rho = \sum_{i=1}^{n} D_i$. By Remark 7, we have $C_\rho \sim B(r,\rho)$. Let A_i be a r.v. such that, if the probing bit of ith MRAD is disturbed or attacked, then $A_i = 1$; otherwise, $A_i = 0$. So we have, $Pr[A_i = 0] = 1 - \kappa$ and $Pr[A_i = 1] = \kappa$. According to Lemma 1, if the probing bit is disturbed or attacked, the probability of a positive detection is $Pr[D_i = 1 | A_i = 1] = \frac{1}{2}$. Otherwise, the probing bit is intact which implies that the result must be negative. Namely, $Pr[D_i = 1 | A_i = 0] = 0$. By Law of total probability,

$$\rho = Pr[D_i = 1] = Pr[D_i = 1 | A_i = 1] \cdot Pr[A_i = 1] + Pr[D_i = 1 | A_i = 0] \cdot Pr[A_i = 0] = \frac{1}{2} \cdot \kappa + 0 \cdot (1-\kappa) = \frac{\kappa}{2}.$$

□

Remark 8. *A binomial distribution* $N(n,p)$ *has mean* np *and variance* $np(1-p)$. *So the binomial distribution* $C_\rho \sim B(r,\rho)$ *in Theorem 5 has mean* $r\rho = \frac{\kappa}{2}r$ *and variance* $r\rho(1-\rho) = \frac{1}{2}r\kappa(1-\frac{\kappa}{2})$ *[26].*

Remark 9. *A binomial distribution* $B(n,p)$ *can be approximated by a normal distribution with the same mean and variance if* $n \geq max\left\{\frac{45(1-2p)^2}{p(1-p)}, \frac{14|1-6p(1-p)|}{p(1-p)}\right\}$ *[27]. Therefore, the binomial distribution* $C_\rho \sim B(r,\frac{\kappa}{2})$ *in Theorem 5 has a normal approximation* $N(\frac{\kappa}{2}r, \frac{1}{2}r\kappa(1-\frac{\kappa}{2}))$ *if* $r \geq max\left\{\frac{180(1-\kappa)^2}{p(2-\kappa)}, \frac{56|1-3\kappa(1-0.5\kappa)|}{\kappa(2-\kappa)}\right\}$.

Theorem 6 provides a method to estimate the parameter p of a Bernoulli distribution [26].

Theorem 6. *Suppose that* $X_i \overset{iid}{\sim} B(1,p)$ *for* $i \in \{1,2,\cdots,n\}$. *Then* $\hat{p} = \bar{X} = \frac{\sum_{i=1}^{n} x_i}{n}$, *an unbiased estimator of* p.

By Theorem 6, $\rho = \frac{\kappa}{2}$ has an unbiased estimator $\hat{\rho} = \widehat{\kappa/2} = \frac{\sum_{i=1}^{r} D_i}{r} = \frac{C_\rho}{r}$. Therefore, κ can be estimated by

$$\hat{\kappa} = \frac{2C_\rho}{r} \ . \tag{1}$$

Remark 9 states that $C_\rho = \sum_{i=1}^{r} D_i \sim B(r,\frac{\kappa}{2})$ approximately follows a normal distribution $N\left(\frac{\kappa r}{2}, \frac{1}{2}r\kappa(1-\frac{\kappa}{2})\right)$. Combining with Fact 2, we conclude that $\widehat{\kappa/2} = \frac{C_\rho}{r} \sim N\left(\frac{\kappa}{2}, \frac{\kappa(1-\frac{\kappa}{2})}{2r}\right)$. Applying Fact 2 again, we have $\hat{\kappa} \sim N\left(\kappa, \frac{2\kappa(1-\frac{\kappa}{2})}{r}\right)$.

4.1 Rate difference monitoring

When ω is unknown, we need to estimate it before starting the execution of the protocol. We have to assume that during this estimation, there is no attack. Under this assumption, κ is reduced to ω, the probability that a probing bit is disturbed. Correspondingly, $\hat{\kappa}$ is reduced to an estimator of ω. Namely, $\kappa = \omega$ and $\hat{\kappa} = \hat{\omega}$. As we have shown $\widehat{\kappa} \sim N\left(\kappa, \frac{2\kappa(1-\frac{\kappa}{2})}{r}\right)$, we also have $\hat{\omega} \sim N\left(\omega, \frac{2\omega(1-\frac{\omega}{2})}{s}\right)$, where s is the number of probing bits for estimating ω. Let C'_ρ denote the number of PMs under the assumption that the probing bits are not attacked. By replacing C_ρ by C'_ρ and s by r in equation (1), we get,

$$\hat{\omega} = \frac{2C'_\rho}{s} \ . \tag{2}$$

In REKSQPC, the attack detection method is implemented by checking that $\kappa = \omega$. After getting the estimations of κ and ω, let e denote their difference, which is an outcome of r.v. $E = \hat{\kappa} - \hat{\omega}$. Fact 3 states that E still follows a normal distribution. In particular, $E \sim N\left(\kappa - \omega, \frac{2\kappa(1-\frac{\kappa}{2})}{r} + \frac{2\omega(1-\frac{\omega}{2})}{s}\right)$. Under the null hypothesis H_0 that there is no attack, $\kappa = \omega$. Then $E \sim N\left(0, 2\nu\left(1 - \frac{1}{2}\nu\right)\left(\frac{1}{r} + \frac{1}{s}\right)\right)$, where $\nu = \kappa = \omega$ and can be estimated by $\hat{\nu} = \frac{2(C'_\rho + C_\rho)}{r+s}$. So, if H_0 is true, the distribution of r.v. E is condensed near zero. Although the set of the possible outcomes of E is $\mathbb{R}$, the test can rule out outcomes that are much greater than zero without introducing much error (note that we do not consider a negative difference because κ is, theoretically, not less than ω. In other words, the alternative hypothesis H_1 is $\kappa > \omega$). Let α denote the probability that an outcome of E is much greater than zero and ruled out by the test. We can test H_0 against H_1 by rejecting H_0 if we observe an outcome of E greater than e_α, where $e_\alpha \in \mathbb{R}$ such that $Pr[E > e_\alpha] = \alpha$. In other words, the protocol is considered insecure if e, the difference between the estimations of κ and ω, is greater than e_α.

The arduous calculation of e_α can be avoided if we scale E to

$$Z = \frac{E - 0}{\sqrt{2\nu\left(1 - \frac{1}{2}\nu\right)\left(\frac{1}{r} + \frac{1}{s}\right)}} = \frac{\hat{\kappa} - \hat{\omega}}{\sqrt{2\hat{\nu}\left(1 - \frac{1}{2}\hat{\nu}\right)\left(\frac{1}{r} + \frac{1}{s}\right)}}, \tag{3}$$

a standard normal distribution according to Fact 1. So correspondingly, the difference e after scaling (denoted by z) is an outcome of Z. Then an equivalent test can be made by rejecting H_0 if $z > z_\alpha$ where $z_\alpha \in \mathbb{R}$ such that $Pr[Z > z_\alpha] = \alpha$. The table listing the value of z_α as a function of α can be found in Reference [26]. Therefore, we amend the original EKSQPC protocol as follows:

Protocol 3 (REKSQPC)**.**

RC1 *(Estimation of ω) Alice and Bob execute MRAD s times. Alice sends s qubits to Bob. He reflects all of them. In other words, there are s probing bits and zero data bits. In Step C5, Alice counts the number of PMs (denoted by C'_ρ). Finally, she uses equation (2) to estimate ω. Note that during the estimation process, we need to guarantee that Eve does not perpetrate attacks.*

RC2 *Alice and Bob start the execution of the protocol. They do Steps C1-C4.*

RC3 *In C5, instead of terminating the protocol when $p_k = 0$ and function $AE(e_{1k}, e_{2k}, i_k) = 1$, Alice increments a counter C_ρ (initial value is zero) and continues to check the remaining bits of $\underset{\sim}{P}$. After finishing checking, she uses equation (1) to estimate κ. We test the null hypothesis $H_0 : \kappa = \omega$ against the alternative hypothesis $H_1 : \kappa > \omega$, equation (3). If H_0 is rejected, Alice considers the protocol is insecure and terminates it; otherwise, Alice and Bob execute Steps C6 and C7 to complete the data transmission.*

When ω is given, we can simply compare it with the estimation of κ. Similarly, we need to test $H_0 : \kappa = \omega$ against $H_1 : \kappa > \omega$. Since ω is not estimated but a given constant, we can say $\hat{\omega} \sim N(\omega, 0)$. We estimate the real attack rate κ by equation (1). Applying Fact 3, we have that $E = \hat{\kappa} - \omega = \hat{\kappa} - \hat{\omega} \sim N\left(\kappa - \omega, \frac{2\kappa(1-\frac{\kappa}{2})}{r}\right)$. Under the assumption that H_0 is true, $E \sim N\left(0, \frac{2\kappa(1-\frac{\kappa}{2})}{r}\right)$. Applying Fact 1, we scale E to $Z' = \frac{(\hat{\kappa}-\omega)-0}{\sqrt{\frac{2\kappa(1-\frac{\kappa}{2})}{r}}} = \frac{\hat{\kappa}-\omega}{\sqrt{\frac{2\kappa(1-\frac{\kappa}{2})}{r}}} \sim N(0,1)$. Let z' denote the scaled difference of the estimated κ and the pre-known ω, which is an outcome of Z'. We reject H_0 if $z' > z_\alpha$, where the definition of z_α is unchanged.

Since ω is given, its estimation is unnecessary. To complete the data transmission, Alice and Bob only need to implement Steps RC2 and RC3, where Z is replaced by Z'.

4.2 Security analysis of REKSQPC

As we have mentioned at the beginning of this section, the original MRAD fails if the qubits transmitted are disturbed and the entanglement is destroyed. The false positives mislead the protocol about the transmission security and cause wrong termination. To fix the problem, in Sections 4.1, we propose a new detection method for MRAs based on a statistical test. The method detects the discrepancy between ω and κ, which does not exist if there is no attack. If any significant discrepancy is identified, the protocol is considered insecure and terminated.

The test rules out the possible outcomes of E that are largely greater than zero, and thus, introduces detection errors. In more details, suppose that Eve does not perpetrate attacks, which implies $\kappa = \omega$ and the null hypothesis H_0 is true. Due to the fluctuation of the estimator D, the difference between $\hat{\kappa}$ and $\hat{\omega}$, there is a proabability α that the sampling of D is greater than the threshold d_α and gets the H_0 rejected, which is a false positive. Correspondingly, if Eve perpetrates attacks and causes $\kappa > \omega$, it is also possible that H_0 is not rejected since their difference is still less than d_α, which is a false negative. We formally define these two types of errors as follows,

Definition 4 (Type A Error - False Negative)**.** *Eve perpetrates an attack, but the protocol is wrongly considered secure.*

Definition 5 (Type B Error - False Positive)**.** *Eve does not perpetrate an attack, but the protocol is wrongly considered insecure.*

The Type A Error has more adverse consequences than the Type B Error because Eve can eavesdrop the message without the awareness of Alice and Bob. We show that the probability of undetected eavesdropping is very low, even when a small number of probing bits is used. The Type B error does not undermine the security of the protocol. Instead, it lowers the transmission efficiency. While Eve does not perpetrate an attack, the Type B error causes a wrong belief of its presence and a termination of the protocol. The protocol needs to restart and resend all qubits. The transmission efficiency is affected.

The choice of a specific value for α, the occurrence probability of the Type B Error, affects the one of the Type A Error. In particular, an increase of α pushes the value of d_α to zero. Although Eve only attacks a few portion of the probing bits, the difference between κ and ω she introduces may still exceed the lowered d_α and get H_0 rejected; therefore, the test becomes stricter and the occurrence probability of Type A Error decreases. Similarly, we can show that a decrease of α leads to an increase of Type A Error occurrence probability. Since the Type A and B Error occurrence probabilities have a negative relationship, if we increase α to enhance the security level, we get more Type B Errors and lower transmission efficiency. Conversely, to decrease the overhead, security is undermined.

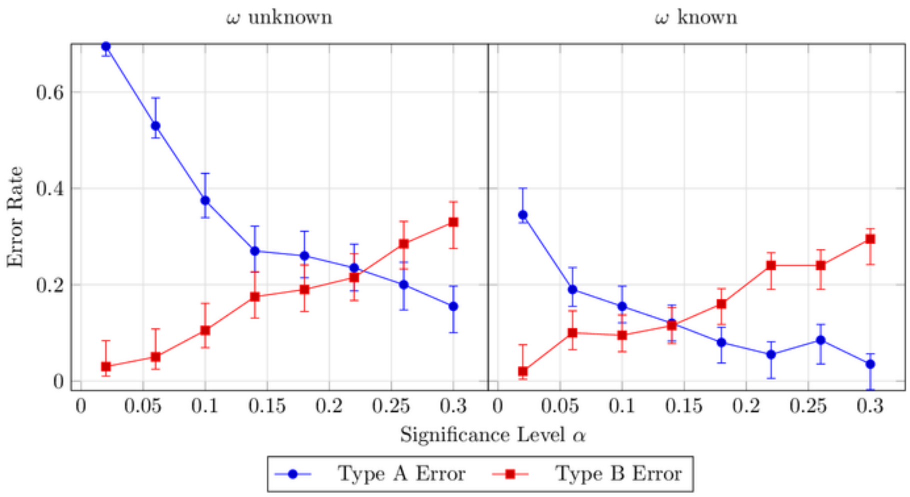

Figure 4. Error rates and their 95% confidence intervals as a function of significance level when the probability ω that a qubit is disturbed is unknown. ($\omega = 0.3$) is unknown (left) and known (right). (Simulation configuration: $r = 600$, $s = 600$ (if ω is unknown), $\omega = 0.3$, $p = 0.1$)

With the results of simulations, Figure 4 plots the rates of the two types of errors as a function of α. For estimating the Type A Error occurrence probability, we set the probability p for Eve to attack a qubit to 10% for both cases, and the number of probing bits to estimate κ and ω (if unknown) to 600. Note that the configuration here is intended to make the Type A Error occurrence probability more sensitive to the choice of alpha, which is not typical in practical problems. We will talk

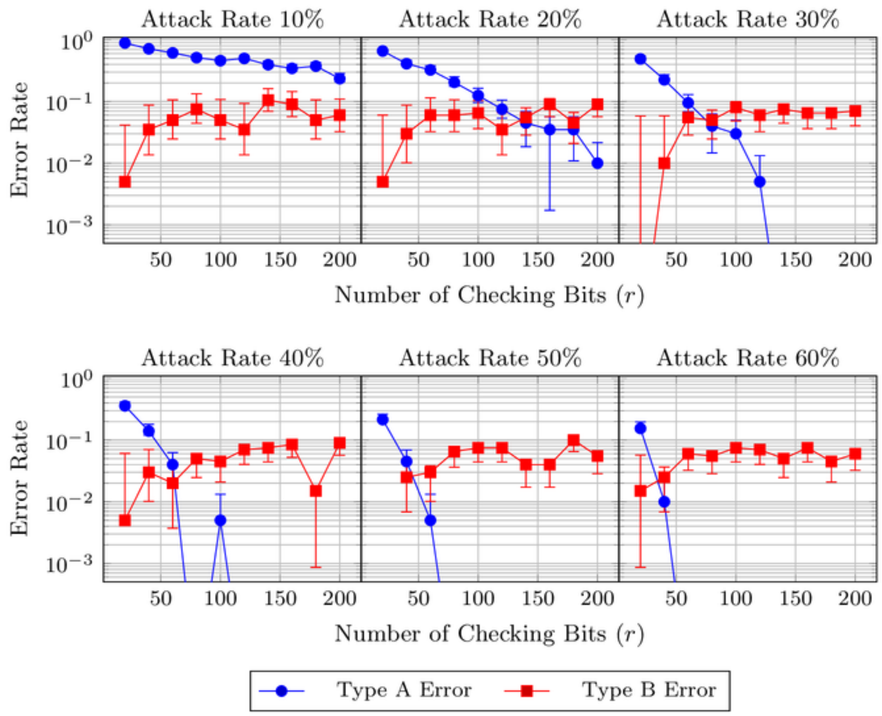

Figure 5. Error rates and their 95% confidence intervals with respect to the number of probing bits when Eve has probability $p = 0.1, 0.2, ..., 0.6$ to attack a qubit. (The probability ω that a qubit is disturbed is unknown. Simulation configuration: $\omega = 0.05$, $\alpha = 0.05$)

about how the error occurrence probabilities behave with more common configurations in the sequel. Whether the value ω is known or not, the trends for both types are consistent with our analysis. When ω is given, the probability of the Type A Error is lower because the estimation of ω introduces more variance, which further amplifies the fluctuation of the estimation of the difference $D = \kappa - \omega$. Regarding the Type B Error, we can observe that the rate roughly equals α which makes sense since it is an estimation of it.

Besides the occurrence probability α of the Type B Error, the numbers of probing bits required to estimate ω and κ are also related to the transmission efficiency. A larger number of probing bits contributes to a better estimation, but also has higher overhead. With the results of simulations, Figures 5 and 6 plot the rates of the Type A and B Errors as a function of the number of probing bits and the attack rate. The probability ($\omega = 0.05$) that a qubit is disturbed is unknown in Figure 5 but pre-known in Figure 6. α is set to 0.05. In Figure 5, the numbers of probing bits coincide for the estimation of ω and κ.

The two figures show that when Eve is more likely to attack a qubit, the detection success rate increases. If Eve only attacks a small proportion of qubits, her attacks do not significantly increase κ and thus are concealed by ω. However, in order to successfully eavesdrop messages, Eve should perpetrates attacks at a rate higher than 50%. When the probability of attacks is 60%, 60 probing bits are sufficient to avoid the Type A Error (when ω is unknown). If ω is given, then 40 probing bits can achieve the same security level.

Note that the Type B Error rate should be constant. In particular, its mean is theoretically equal to 5% as it is an estimation of α. However, while the estimated rate roughly stays around 5% in Figure 6, a relatively considerable increase is observed in Figure 5. The increase is due to a low number of probing bits. According to Remark 9, a good normal approximation requires a large sample size and to estimate both ω and κ, a even larger one is needed. Although, the approximation is not quite accurate when the probing bit number is small, a low level of Type A Error rate shows that it is good enough to secure the protocol.

Since the REKSQPC and EKSQPC protocols are the same except for the part that detects MRAs, Theorem 4 is also applicable to REKSQPC. In particular, we have Theorem 7.

Theorem 7. *With a sufficient number of probing bits, the Type A Error can be avoided. So the REKSQPC protocol is secure.*

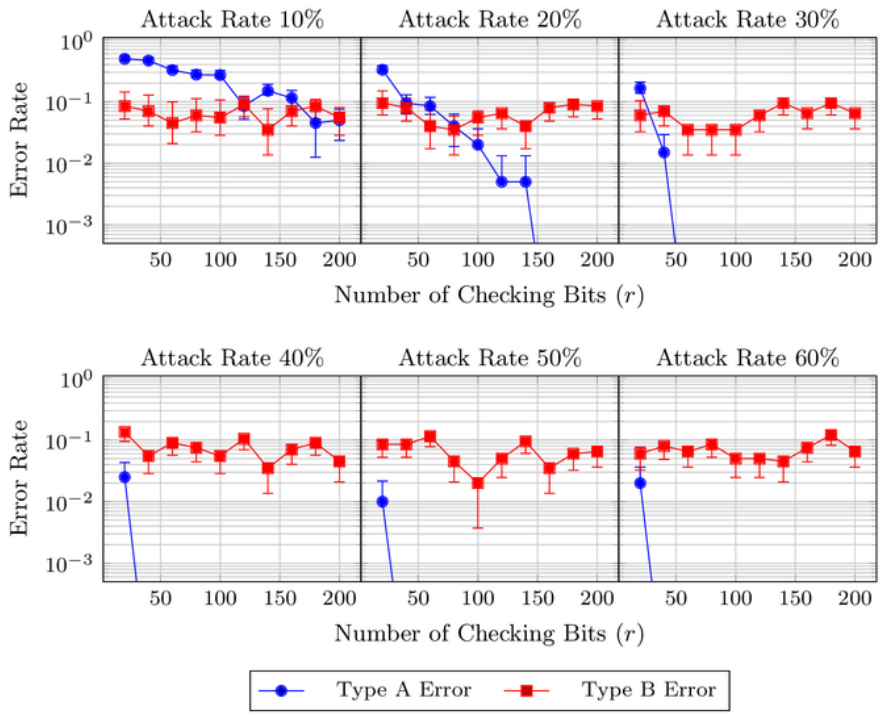

Figure 6. Error rates and their 95% confidence intervals with respect to the number of probing bits when Eve has probability $p = 0.1, 0.2, ..., 0.6$ to attack a qubit. (The probability ω that a qubit is disturbed is known. Simulation configuration: $\omega = 0.05$, $\alpha = 0.05$)

5 Quantum Resource Requirements and Transmission Efficiency

In this section, we analyze the requirements of quantum resources, qubit efficiency and quantum circuit complexity of the EKSQPC protocol. Among all SQKD and SQDC protocols, we show that the EKSQPC protocol has the highest qubit efficiency (almost 100%) with the simplest quantum circuits (without qubits permutation and measurement basis switch). Comparing to the protocols utilizing the quantum entanglements, we show that the EKSQPC protocol reaches the theoretical minimum of the quregister size and the EPT among the SQKD and SQDC protocols.

5.1 Quantum resources requirements

We briefly discuss the quantum resources requirements at the end of Section 3.2. In this section, we elaborate them. Since the revised version introduced in Section 4 does not change the hardware requirements, we discuss them together.

Alice and Bob loop over i_k in I to accomplish all the operations requiring quantum resources. Hence, the lowest quantum resources requirements of the protocol implementation agrees with the one to execute a single quantum procedure group QPG_k (plotted in Figure 7).

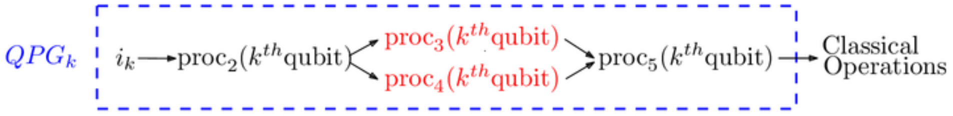

Figure 7. The EKSQPC protocol flow diagram of the procedures requiring quantum resources. *The procedures marked in red belong to Bob. For each QPG_k, Bob chooses either $proc_3(k^{th}$ qubit$)$ to measure or $proc_4(k^{th}$ qubit$)$ to reflect.*

In order to generate EPR pairs in Procedure 2, Alice is required to have an EPR pair generation circuit. Besides, a one-bit quregister is needed to retain the first qubit of the pair. Alice needs a Bell measurement circuit to perform Procedure 5. The entanglement of the EPR pair must be preserved until Alice applies a Bell measurement on it (this case happens when the

second qubit γ_B is reflected by Bob. Otherwise, Bob measures it, and the preservation time is shorter). Let C denote the time that Alice generates, sends and receives the qubits, and T the one-way time for the qubits to move between Alice and Bob. Then the EPT is $C+2T$ if we do not count the qubits reflection time of Bob. Compared to Alice, the quantum capability of Bob is fundamental. In particular, he should be able to either measure a qubit in the Z-basis, followed by sending a pre-generated $|0\rangle$, or reflect it. Overall, Alice and Bob need the following minimum quantum capabilities for the protocol implementation.
Alice: a one-bit quregister, the circuits for Bell measurement and EPR pair generation.
Bob: a device that either reflects a qubit or uses the Z-basis to measure it followed by sending a $|0\rangle$.

Among SQDC and SQKD protocols that utilize entanglements, Alice must create at least a pair of entangled qubits and send at least one of the qubits to Bob. Therefore, for containing a qubit, a one-bit quregister is necessary. For checking the potential attacks, Alice must do some quantum operations on the qubit pair consisting of the qubit she retained and the one reflected by Bob. So, the EPT is at least $C+2T$. As the EKSQPC reaches the theoretical lower bound, we conclude that,

Theorem 8. *Among SQDC and SQKD protocols that utilize entanglements, the EKSQPC protocol only requires theoretically minimal quregister size and EPT.*

5.2 Transmission efficiency

Suppose the string $\underset{\sim}{C}$ $(=\underset{\sim}{U})$ shared by Alice and Bob is updated for each dialogue. In other words, prior to applying Step C6 and Step C7 to transmit data, Alice and Bob always execute Steps C1 to C5 to share a random binary string.

Consider the original EKSQPC protocol. Suppose that an s-bit message is sent to Bob from Alice, and for each qubit Eve has probability p to perpetrate an MRA. Then, for a high eavesdropping efficiency, Eve has to choose a p close to one. Assume $p=0.6$ and the number of probing bits is 15. Theorem 3 shows that the success rate of detection is higher than 0.995. Moreover, adding a few more probing bits can enhance the security level significantly. In practice, the message length s should considerably exceed 15. Then the probing bits can only introduce a negligible overhead. So for sending a message of length s, Alice sends roughly s qubits to Bob. Thus, the qubit efficiency approaches 100%.

If we consider the possibility that a qubit is disturbed during the qubit transmission, we need to apply the rate estimation version of the protocol. If ω is unknown, we need around 60 probing bits to reach 99% detection success rate (assuming that $\alpha=0.01$, $\omega=0.05$), which is acceptable considering a much larger total number of qubits transmitted. If the rate is given, then the probing bit number can decrease to 30 (extra 20 probing bits can improve the success rate to almost 100%). Then the overhead from the probing bits is negligible. The major part of the overhead is from α, the probability to get Type B Error, which causes a full restart of the protocol. In average, $\alpha \cdot 100\%$ qubits transmitted are discarded due to the wrong conclusion that the protocol is insecure. If we choose $\alpha=0.01$ (which is big enough to secure the protocol), the overhead is only 1%.

Notice that Alice is only required to perform Bell measurements. So she only needs a fixed circuit without measurement basis switch capability. Additionally, the operations related to one qubit is irrelevant to those concerned with the others (since the message security does not depend on the bit permutation by Alice and Bob). Therefore, if the transmission or measurement of a single qubit fails, Alice and Bob only need to re-implement the operations associated with that qubit. This enhances the success rate and efficiency of the data transmission potentially.

In Table 3, we make a detailed comparison with other typical SQKD and SQDC protocols. Note that the qubit efficiency (η) is calculated by

$$\eta = \frac{\text{Length of the message}}{\text{Number of qubits sent by Alice}}.$$

For the protocols in References [7–9, 28], η depends on some parameters other than the length of the message. For these protocols, we give an upper bound for η. Regarding REKSQPC, η is calculated by choosing $\alpha=0.01$. Besides, in the protocol proposed by Li et al. [9], the measurement basis switch is not required of Alice or Bob but delegated to a third full quantum capability computer Charlie.

6 Conclusion

In this paper, we proposed a new SQDC protocol (named Economic Keyless Semi-Quantum Point-to-Point Communication). Compared to other SQDC and SQKD protocols, our new protocol has much higher qubit efficiency (almost 100%) and simpler quantum circuits (not requiring switching measurement basis or permuting qubits). While other SQKD and SQDC protocols encrypting messages through entanglements require at least linear EPT and linear size quregister, in our protocol, only Alice is required to have a fixed size (as low as one) quregister and preserve an EPR pair entanglement for time $C+2T$, where C is the time that Alice prepares, receives and measures the qubits, and T is the one for the qubits to move between Alice and Bob.

Among the protocols using quantum entanglements to encrypt messages, we show that both quregister size and EPT achieve the theoretical minimums. A pre-shared key is not required by our new protocol. Instead, Alice and Bob use the qubits

entanglement to share a random string and further use it as a key to secure the data transmission. We used the probing bits to implement MRAD so that the protocol is resistant to MRAs.

In our original protocol, Theorem 3 shows that 15 probing bits can lead to a 0.995 success rate of attack detection (given that the adversary Eve has the probability 0.6 of perpetrating an MRA on a single qubit). A few more bits can boost the security level of the protocol significantly (for example, 0.9992 detection success rate can be achieved by using 20 probing bits). If the message size is sufficiently long, then the qubit efficiency can reach almost 100%.

The rate estimation version, the protocol REKSQPC, can function properly and correctly detect attacks perpetrated by Eve while the qubits may be disturbed during the transmission. We designed a test to monitor the difference of κ, the probability that a qubit is disturbed or attacked, and ω (estimated or pre-known), the probability that a qubit is disturbed. If the difference is significantly large, the protocol terminates. The simulation results show that 60 probing bits can push detection success rate to almost 100% (assuming that $\alpha = 0.05$ and $\omega = 0.05$) if ω is unknown. The number of probing bits can decrease to 40 and achieve the same success rate if ω is pre-known. Assuming that we can always detect MRAs, our protocol is secure against network attacks (Theorems 4 and 7).

References

1. Shor, P. W. & Preskill, J. Simple proof of security of the bb84 quantum key distribution protocol. *Phys. Rev. Lett.* **85**, 441–444 (2000).
2. Ekert, A. K. Quantum cryptography based on bell's theorem. *Phys. Rev. Lett.* **67**, 661–663 (1991).
3. Long, G. L. & Liu, X. S. Theoretically efficient high-capacity quantum-key-distribution scheme. *Phys. Rev. A* **65**, 032302 (2002).
4. Lo, H.-K. & Chau, H. F. Unconditional security of quantum key distribution over arbitrarily long distances. *Sci.* **283**, 2050–2056 (1999).
5. Shor, P. W. Polynomial-time algorithms for prime factorization and discrete logarithms on a quantum computer. *SIAM J. on Comput.* **26**, 1484–1509 (1997).
6. Boyer, M., Kenigsberg, D. & Mor, T. Quantum key distribution with classical bob. *Phys. Rev. Lett.* **99**, 140501 (2007).
7. Boyer, M., Gelles, R., Kenigsberg, D. & Mor, T. Semiquantum key distribution. *Phys. Rev. A* **79**, 032341 (2009).
8. Jian, W., Sheng, Z., Quan, Z. & Chao-Jing, T. Semiquantum key distribution using entangled states. *Chin. Phys. Lett.* **28**, 100301 (2011).
9. Li, Q., Chan, W. H. & Zhang, S. Semiquantum key distribution with secure delegated quantum computation. *Sci. Reports* **6** (2016).
10. Luo, Y.-P. & Hwang, T. Authenticated semi-quantum direct communication protocols using bell states. *Quantum Inf. Process.* **15**, 947–958 (2016).
11. Almousa, S. & Barbeau, M. Delay and reflection attacks in authenticated semi-quantum direct communications. *2016 IEEE Globecom Work. (GC Wkshps)* 1–7 (2016).
12. Shukla, C., Thapliyal, K. & Pathak, A. Semi-quantum communication: protocols for key agreement, controlled secure direct communication and dialogue. *Quantum Inf. Process.* **16**, 295 (2017).
13. Zhang, W. *et al.* Quantum secure direct communication with quantum memory. *Phys. Rev. Lett.* **118**, 220501 (2017).
14. Wu, F. *et al.* High-capacity quantum secure direct communication with two-photon six-qubit hyperentangled states. *Sci. China Physics, Mech. & Astron.* **60**, 120313 (2017).
15. Gu, J., Lin, P.-h. & Hwang, T. Double c-not attack and counterattack on 'three-step semi-quantum secure direct communication protocol'. *Quantum Inf. Process.* **17**, 182 (2018).
16. Zhong, M. *et al.* Optically addressable nuclear spins in a solid with a six-hour coherence time. *Nat.* **517**, 177–180 (2015).
17. Inagaki, T., Matsuda, N., Tadanaga, O., Asobe, M. & Takesue, H. Entanglement distribution over 300 km of fiber. *Opt. Express* 23241–23249.

18. Neumann, P. *et al.* Quantum register based on coupled electron spins in a room-temperature solid. *Nat Phys* **6**, 249–253 (2010).

19. Dai, H.-N. *et al.* Generation and detection of atomic spin entanglement in optical lattices. *Nat Phys* **12**, 783–787 (2016).

20. Lu, H., Barbeau, M. & Nayak, A. Economic no-key semi-quantum direct communication protocol. In *2017 IEEE Globecom Workshops (GC Wkshps)*, 1–7 (2017).

21. Malladi, S., Alves-Foss, J. & Heckendorn, R. B. On preventing replay attacks on security protocols. *In* Proc. Int. Conf. on Secur. Manag. 77–83 (2002).

22. Scarani, V., Acín, A., Ribordy, G. & Gisin, N. Quantum cryptography protocols robust against photon number splitting attacks for weak laser pulse implementations. *Phys. Rev. Lett.* **92**, 057901 (2004).

23. Lo, H.-K., Ma, X. & Chen, K. Decoy state quantum key distribution. *Phys. Rev. Lett.* **94**, 230504 (2005).

24. Kalashnikov, D. A., Tan, S. H., Chekhova, M. V. & Krivitsky, L. A. Accessing photon bunching with a photon number resolving multi-pixel detector. *Opt. Express* **19**, 9352–9363 (2011).

25. Zhou, Z. *et al.* Superconducting series nanowire detector counting up to twelve photons. *Opt. Express* **22**, 3475–3489 (2014).

26. Hogg, R., Tanis, E. & Zimmerman, D. *Probability and Statistical Inference*, 192 (Pearson Education, 2014).

27. DasGupta, A. *Normal Approximations and the Central Limit Theorem*, 213–242 (Springer New York, New York, NY, 2010).

28. Zou, X., Qiu, D., Li, L., Wu, L. & Li, L. Semiquantum-key distribution using less than four quantum states. *Phys. Rev. A* **79**, 052312 (2009).

Author Contributions

H.L devised the protocol. H.L and M.B wrote the main manuscript, and A.N gave solid suggestions on the manuscript. All authors reviewed the manuscript.

Additional Information

Competing interests: The authors declare no competing interests.

Table 1. Bell measurements on Bell states.

$\gamma_A\gamma_B$	Output (e_1e_2)	$\gamma_A\gamma_B$	Output (e_1e_2)
$\|\Phi^+\rangle$	00	$\|\Phi^-\rangle$	10
$\|\Psi^+\rangle$	01	$\|\Psi^-\rangle$	11

Table 2. Bell measurement results of $|00\rangle$, $|01\rangle$, $|10\rangle$ and $|11\rangle$; there is 0.5 possibility for each output.

$\gamma_A\gamma_B$	Output (e_1e_2)	$\gamma_A\gamma_B$	Output (e_1e_2)
$\|00\rangle$	00	$\|01\rangle$	01
	10		11
$\|10\rangle$	01	$\|11\rangle$	00
	11		10

Table 3. Comparisons among typical SQKD and SQDC protocols.

Protocols (The protocols using entanglements are marked by *)	Qubit permutation	Basis Switch	Qubit Efficiency η	Minimum # quregisters	EPT
Boyer(2009) Randomization-Based SQKD [7]	Yes	Yes	$< 12.5\%$	$4n$	0
Boyer(2009) Measure-Resend SQKD [7]	No	Yes	$< 12.5\%$	0	0
Zou (2009) Protocol 5 [28]	No	Yes	$< 12.5\%$	0	0
Wang (2011) [8] *	Yes	Yes	$< 50\%$	$6n$	Worse than linear
Li (2016) [9]	No	Yes	$< 6.25\%$	0	0
Luo (2016) [10] *	Yes	Yes	12.5%	$20n$	Worse than linear
EKSQPC *	No	No	$\approx 100\%$	1	$C+2T$
REKSQPC *	No	No	$\approx 99\%$	1	$C+2T$

Revised Version

ORIGINALITY REPORT

10%	7%	9%	3%
SIMILARITY INDEX	INTERNET SOURCES	PUBLICATIONS	STUDENT PAPERS

PRIMARY SOURCES

1	Haoye Lu, Michel Barbeau, Amiya Nayak. "Economic No-Key Semi-Quantum Direct Communication Protocol", 2017 IEEE Globecom Workshops (GC Wkshps), 2017<br>Publication	1%
2	Krawec, Walter O.. "Security of a semi-quantum protocol where reflections contribute to the secret key", Quantum Information Processing, 2016.<br>Publication	<1%
3	Lecture Notes of the Institute for Computer Sciences Social Informatics and Telecommunications Engineering, 2010.<br>Publication	<1%
4	Xu-Dong Wu, Lan Zhou, Wei Zhong, Yu-Bo Sheng. "Purification of the concatenated Greenberger–Horne–Zeilinger state with linear optics", Quantum Information Processing, 2018<br>Publication	<1%
5	hal.upmc.fr<br>Internet Source	<1%

6	Jeong, Youn-Chang, Yong-Su Kim, Yoon-Ho Kim, Yanhua Shih, and Keith S. Deacon. "", Quantum Communications and Quantum Imaging VIII, 2010.<br>Publication	<1%
7	Anindita Banerjee, Kishore Thapliyal, Chitra Shukla, Anirban Pathak. "Quantum conference", Quantum Information Processing, 2018<br>Publication	<1%
8	Wei Zhang, Daowen Qiu, Paulo Mateus. "Security of a single-state semi-quantum key distribution protocol", Quantum Information Processing, 2018<br>Publication	<1%
9	Steinert, S., F. Ziem, L. T. Hall, A. Zappe, M. Schweikert, N. Götz, A. Aird, G. Balasubramanian, L. Hollenberg, and J. Wrachtrup. "Magnetic spin imaging under ambient conditions with sub-cellular resolution", Nature Communications, 2013.<br>Publication	<1%
10	e3.physik.uni-dortmund.de<br>Internet Source	<1%
11	Zhong, Tian, Jonathan M. Kindem, Evan Miyazono, and Andrei Faraon. "Nanophotonic	<1%

coherent light–matter interfaces based on rare-earth-doped crystals", Nature Communications, 2015.
Publication

12	Huawang Qin, Wallace K. S. Tang, Raylin Tso. "Establishing rational networking using the DL04 quantum secure direct communication protocol", Quantum Information Processing, 2018<br>Publication	<1%
13	Chitra Shukla, Kishore Thapliyal, Anirban Pathak. "Semi-quantum communication: protocols for key agreement, controlled secure direct communication and dialogue", Quantum Information Processing, 2017<br>Publication	<1%
14	Reichardt, Ben W., Falk Unger, and Umesh Vazirani. "Classical command of quantum systems", Nature, 2013.<br>Publication	<1%
15	pdfs.semanticscholar.org<br>Internet Source	<1%
16	www.scribd.com<br>Internet Source	<1%
17	eprints.soton.ac.uk<br>Internet Source	<1%

18	Submitted to Sim University<br>Student Paper	<1 %
19	Craig A. Depken. "Another look at anti-scalping laws: Theory and evidence", Public Choice, 01/03/2007<br>Publication	<1 %
20	dblp2.uni-trier.de<br>Internet Source	<1 %
21	eprints.qut.edu.au<br>Internet Source	<1 %
22	academic.uprm.edu<br>Internet Source	<1 %
23	Zeng. "Quantum Key Distribution", Quantum Private Communication, 2010<br>Publication	<1 %
24	www.mdpi.com<br>Internet Source	<1 %
25	Shaoying Yin, Jie Song, Xuexin Xu, Yujun Zhang, Shutian Liu. "Quantum coherence dynamics of three-qubit states in XY spin-chain environment", Quantum Information Processing, 2018<br>Publication	<1 %
26	Kishore Thapliyal, Anirban Pathak. "Kak's three-stage protocol of secure quantum	<1 %

communication revisited: hitherto unknown strengths and weaknesses of the protocol", Quantum Information Processing, 2018
Publication

27	walterkrawec.org<br>Internet Source	<1%
28	Lin Liu, Min Xiao, Xiuli Song. "Authenticated semiquantum dialogue with secure delegated quantum computation over a collective noise channel", Quantum Information Processing, 2018<br>Publication	<1%
29	www.ma.huji.ac.il<br>Internet Source	<1%
30	d-nb.info<br>Internet Source	<1%
31	rd.springer.com<br>Internet Source	<1%
32	Qin Li, Wai Hong Chan, Shengyu Zhang. "Semiquantum key distribution with secure delegated quantum computation", Scientific Reports, 2016<br>Publication	<1%
33	Michele Boreale. "Attacking Right-to-Left Modular Exponentiation with Timely Random Faults", Lecture Notes in Computer Science,	<1%

2006
Publication

34	athens.src.uchicago.edu<br>Internet Source	<1%
35	Submitted to University of Warwick<br>Student Paper	<1%
36	jsdajournal.springeropen.com<br>Internet Source	<1%
37	osaptesting.osa.org<br>Internet Source	<1%
38	Kun-Fei Yu, Jun Gu, Tzonelih Hwang, Prosanta Gope. "Multi-party semi-quantum key distribution-convertible multi-party semi-quantum secret sharing", Quantum Information Processing, 2017<br>Publication	<1%
39	Springer Series on Fluorescence, 2015.<br>Publication	<1%
40	Submitted to Queen Mary and Westfield College<br>Student Paper	<1%
41	RENATO RENNER. "SECURITY OF QUANTUM KEY DISTRIBUTION", International Journal of Quantum Information, 2008<br>Publication	<1%

42	www.lbd.dcc.ufmg.br<br>Internet Source	<1%
43	www.bing.com<br>Internet Source	<1%
44	Butler, Richard, Timothy J. Dodwell, Raphael T. Haftka, Nam Ho Kim, Tatiana Kim, Stephanie Kynaston, and Rob Scheichl. "Uncertainty Quantification of Composite Structures with Defects using Multilevel Monte Carlo Simulations", 17th AIAA Non-Deterministic Approaches Conference, 2015.<br>Publication	<1%
45	Lecture Notes in Computer Science, 2013.<br>Publication	<1%
46	N. Lütkenhaus. "Secret keys from quantum correlations", Informatik - Forschung und Entwicklung, 10/05/2006<br>Publication	<1%
47	www.thinkmind.org<br>Internet Source	<1%
48	www.amast.org<br>Internet Source	<1%
49	link.springer.com<br>Internet Source	<1%
50	Zhulin Li, Qin Li, Chengdong Liu, Yu Peng, Wai	

Hong Chan, Lvzhou Li. "Limited resource semiquantum secret sharing", Quantum Information Processing, 2018
Publication | <1%

---

51 rotemliss.cswp.cs.technion.ac.il
Internet Source | <1%

---

52 Curty, Marcos, Feihu Xu, Wei Cui, Charles Ci Wen Lim, Kiyoshi Tamaki, and Hoi-Kwong Lo. "Finite-key analysis for measurement-device-independent quantum key distribution", Nature Communications, 2014.
Publication | <1%

---

53 de2de.synechism.org
Internet Source | <1%

---

54 tuprints.ulb.tu-darmstadt.de
Internet Source | <1%

---

55 greenteapress.com
Internet Source | <1%

---

56 Arpita Maitra. "Measurement device-independent quantum dialogue", Quantum Information Processing, 2017
Publication | <1%

---

57 Chen Xie, Lvzhou Li, Haozhen Situ, Jianhao He. "Semi-quantum Secure Direct Communication Scheme Based on Bell States", International Journal of Theoretical Physics, | <1%

2018
Publication

58	www.walterkrawec.org<br>Internet Source	<1%
59	Li, Chuan-Ming, Kun-Fei Yu, Shih-Hung Kao, and Tzonelih Hwang. "Authenticated semi-quantum key distributions without classical channel", Quantum Information Processing, 2016.<br>Publication	<1%

Exclude quotes	Off	Exclude matches	Off
Exclude bibliography	Off		

Revised Version

PAGE 1

PAGE 2

PAGE 3

PAGE 4

PAGE 5

PAGE 6

PAGE 7

PAGE 8

PAGE 9

PAGE 10

PAGE 11

PAGE 12

PAGE 13

PAGE 14

PAGE 15

PAGE 16

Original Version

by Haoye Lu

Submission date: 28-Nov-2018 09:53AM (UTC-0800)
Submission ID: 1044157789
File name: main.pdf (407.93K)
Word count: 10298
Character count: 51345

No-Key Semi-Quantum Direct Communication Protocol with Low Quantum Resource Requirements

Haoye Lu[1,*], **Michel Barbeau**[2], **and Amiya Nayak**[1]

[1]University of Ottawa, School of Electrical Engineering and Computer Science (EECS), Ottawa, K1N 6N5, Canada
[2]Carleton University, School of Computer Science, Ottawa, K1S 5B6, Canada
[*]hlu044@uottawa.ca

ABSTRACT

Full quantum capability devices can provide secure communications, but they are challenging to make portable given the current technology. Besides, classical portable devices are unable to construct communication channels resistant to quantum computers. Hence, communication security on portable devices cannot be guaranteed. Semi-Quantum Key Distribution (SQKD) and Semi-Quantum Direct Communication (SQDC) attempt to break the quandary by lowering the receiver's required quantum capability so that secure communications can be implemented on a portable device. However, all SQKD and SQDC protocols have low qubit efficiency and complex hardware implementations. The protocols involving quantum entanglement require linear Entanglement Preservation Time (EPT) and linear quregister size. In this paper, we propose two new no-key SQDC protocols that address the aforementioned weaknesses. They are named Economic No-key SQDC (ENKSQDC) and Rate Estimation ENKSQDC (RENKSQDC). They achieve theoretically constant minimal EPT and quregister size, regardless of message length. We show that the new protocols, with low overhead, can detect Measure and Replay Attacks (MRAs). RENKSQDC is tolerant to transmission impairments and environmental perturbations. The protocols are based on a new quantum message transmission operation termed Tele-Conjure. Like QKD, their strength depends on physical principles rather than mathematical complexity.

1 Introduction

Two full quantum capability devices can communicate securely by Quantum Key Distribution (QKD) [1–4]. In these protocols, two communicants have to be armed with advanced quantum components including quantum registers, programmable quantum circuits, quantum generators, etc.. Most of them can only function under stable and well-configured environments and occupy large space. So, it is challenging to implement secure communications on portable devices. However, quantum computers can efficiently break RSA cryptosystem [5], the security foundation of almost all classical communication protocols. Thence, the communication security of portable devices is in imminent danger of collapse.

Semi-Quantum Key Distribution (SQKD) and Semi-Quantum Direct Communication (SQDC) try to break the predicament by limiting the quantum capability of the receiver without dampening the transmission security. The quantum components for realizing limited quantum capability can be designed compact, simple and robust so that they could be integrated into a portable device. The discussions start from two new SQKD protocols proposed by Boyer et al. [6, 7]. Compared with QKD, the receiver Bob needs only to perform four quantum operations: (1) prepare quantum bits (qubits) in the Z-basis, (2) measure qubits in the Z-basis, (3) reorder qubits and (4) access quantum channel. These two new protocols secure the communications by randomizing measurement basis and Bob's treatment on the qubits he receives. For concealing Bob's behaviour, reordering of the qubits is also required. In 2011, Jian et al. [8] proposed a new SQKD protocol that improves qubit efficiency (the ratio of the length of the message to the one of the qubits sent by Alice) from the original 12.5% to roughly 50% by using entangled qubits . But the Entanglement Preservation Time (EPT) for implementing the protocol is at least linear to the length of the message. So is the quantum bit register (quregister) size. Li et al. [9] showed that Bob's quantum computation task can be delegated to a third quantum server in semi-quantum communications at the cost of a low qubit efficiency (6.25%). In 2015, Luo and Hwang [10] proposed a new protocol showing that the authentic classical channel is unnecessary if the two communicants have a pre-shared key. However, besides a even longer EPT and a low qubit efficiency (12.5%), a larger quregister size is required for each data bit. A similar pre-shared key based protocol proposed by Almousa and Barbeau [11] shows Bob does not need to store any qubits, but the linear EPT persists.

All the aforementioned protocols [6–11] suffer from low qubit efficiency. Most of them have significant large linear quregister size overhead and require permutation of qubits [6–8, 10, 11]. Regarding the protocol involving entangled qubits [8, 10, 11], the quantum EPT is at least linear. Although a six-hour record has been achieved by Zhong et al. using europium ion embedded in a crystal [12], entanglement time does decrease dramatically should we transmit entangled photons through an optical fiber (required by almost all quantum communication protocols) [13]. Besides, involving permutations on qubits

(not practical shortly) dooms to a low transmission efficiency and reliability. Considering that the unusual materials (such as ultracold atoms by Dai et al. [14] and coupled electron by Neumann et al. [15]) are necessary for the implementation of quregisters, a commercial quantum network based on them is not feasible in a near future.

This paper introduces a new SQDC protocol and rate estimation version, called Economic No-Key Semi-Quantum Direct Communication (ENKSQDC) and Rate Estimation ENSQDC (RENKSQDC), which address all the aforementioned issues. An innovative operation, called Tele-Conjure (TC), utilizes entangled qubit pairs to transmit messages. It is at the core of Single-Bit Protocol (SBP). The results of measurements on the pairs fall in a predesigned set because of the entanglements, but do not carry any useful information. The design makes SBP functioning without a pre-shared key and fully resistant to information leakage even if the qubits are intercepted. Besides, the protocol uses the same quantum circuit as the one to detect the Measure and Replay Attack (MRA) (called MRA Detection (MRAD)) and thus, not only saves the quantum resources but also becomes the cornerstone of the ENKSQDC and RENKSQDC protocols. Because Alice performs the same quantum operations in both protocols (SBP or MRAD), Bob does not need to communicate with Alice until all quantum operations (Alice's and Bob's) are completed. Alice and Bob execute MRADs using a small portion of the measurement results before using the classical channel to translate the rest into valid messages. Assuming that MRAs are always detectable, we prove that the protocol is fully secure. As the pivot to secure the messages is a successful detection of MRAs, we show that the attack detection success rate can reach 99.5% with only 15 checking bits (if the adversary Eve has 60% probability of attacking a qubit). A few extra checking bits can enhance the protocol security to a large extent. The qubit efficiency can reach almost 100% if the message is sufficiently long. Regarding quantum resource requirements, in the ENKSQDC protocol, the size of the quregister required by Alice is as low as one, while the required EPT is $2T+C$ (T is the one-way traveling time between Alice and Bob, and C is the processing time for Alice to prepare, send and receive the qubits). We prove that both the quregister size and EPT reach the theoretical minimums.

Considering that the entanglement of qubits may not always persist during the qubits transmission, we assume that there is a probability ω that the entanglement involving a qubit is destroyed as it is disturbed by a hardware imperfection or an environmental disturbance. Under this assumption, we design a statistical test to compare ω with the probability that a qubit is attacked or disturbed. When a significant difference is observed, Alice concludes that Eve perpetrated attacks and aborts the execution of the protocol. Therefore, the communication is not eavesdropped successfully. Compared with the original ENKSQDC, more checking bits are required to achieve the same detection success rate; however, the overhead is still low. In particular, our simulation results reveal that 60 checking bits are enough to detect almost all attacks when ω is unknown. If the rate is given, then 40 checking bits are enough to achieve the same detection success rate.

The rest of the paper is organized as follows. In Section 2, we introduce the notations. Then, we review Bell measurement and MRAD, which are used repetitively in the new protocol. In Sections 3 and 4, we introduce our new protocols including a rate estimation version taking into account the probability that a qubit is disturbed. We also present security analysis and discuss simulation results. In Section 5, we talk about their quantum resource requirements and transmission overhead. Finally, we draw the conclusions in Section 6. This paper is a revised and extended version of a preliminary workshop paper [16].

2 Background

We start this section by introducing the notations. Since our new protocol heavily relies on the Bell states and Bell measurement, we briefly review them first. We also introduce MRAs and the corresponding detection algorithm (MRAD), which is used in our new protocol to guarantee the data transmission security.

In this paper, we use lowercase English letters to represent classical bits (cbits) and Greek letters to represent qubits. Besides, bold English capital letters denote Bell states. A sequence of cbits is denoted by an uppercase English letter with a tilde underneath. For instance, $\underset{\sim}{M} = m_1 m_2 \cdots m_\iota$ is a cbit string of length ι.

2.1 Bell states and Bell measurement

The Bell states are considered the simplest example of entanglement. There are four Bell states: $|\Phi^+\rangle = \frac{|00\rangle+|11\rangle}{\sqrt{2}}$, $|\Phi^-\rangle = \frac{|00\rangle-|11\rangle}{\sqrt{2}}$, $|\Psi^+\rangle = \frac{|01\rangle+|10\rangle}{\sqrt{2}}$, $|\Psi^-\rangle = \frac{|01\rangle-|10\rangle}{\sqrt{2}}$, which can be distinguished by Bell measurement (B.M.) (Figure 1). The circuit receives two qubits λ_A and λ_B and outputs two cbits b_1 and b_2. In particular, if the input $\lambda_A\lambda_B$ is one of four Bell states, we have

$$\lambda_A\lambda_B = |\Phi^+\rangle \xrightarrow{\text{B.M.}} b_1b_2 = 00, \qquad \lambda_A\lambda_B = |\Phi^-\rangle \xrightarrow{\text{B.M.}} b_1b_2 = 10,$$
$$\lambda_A\lambda_B = |\Psi^+\rangle \xrightarrow{\text{B.M.}} b_1b_2 = 01, \qquad \lambda_A\lambda_B = |\Psi^-\rangle \xrightarrow{\text{B.M.}} b_1b_2 = 11.$$

If $\lambda_A\lambda_B$ is not a Bell state, it is projected into a Bell state (according to the quantum measurement projection rule). In particular, we list the cases when input $\lambda_A\lambda_B$ equals $|00\rangle$, $|01\rangle$, $|10\rangle$ or $|11\rangle$ in Table 1.

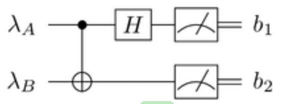

Figure 1. The Bell measurement Circuit. *If the input* $\lambda_A \lambda_B$ *is one of four Bell states* $|\Phi^+\rangle$, $|\Phi^-\rangle$, $|\Psi^+\rangle$, $|\Psi^-\rangle$, *the outputs* $b_1 b_2$ *are* 00, 10, 01, 11 *respectively.*

2.2 Measure and Replay Attack and the detection method

The replay attack is a classical network threat in which the adversary Eve replays a message in a context different from the original and expected context, therefore tricking the honest listener(s) into believing it has completed the protocol execution successfully [17].

In a quantum network, a similar attack can be perpetrated. Suppose Eve can intercept the quantum channel connecting Alice and Bob and measure any qubit in it. While Alice sends qubits to Bob through the channel, Eve measures the qubits using the Z-basis ($|0\rangle$, $|1\rangle$) and sends new qubits to Bob according to the measurement results (if the result is zero, Eve sends $|0\rangle$; else, she sends $|1\rangle$). Since Eve needs to measure the qubits before replaying them, the attack is called the Measure and Replay Attack (MRA) [11].

The following procedure shows how we detect MRAs using the Bell state entanglement property and Bell measurement. The idea is also used in the Luo and Hwang's protocol [10] and inherited by the Almousa and Barbeau's protocol [11].

MRA Detection (MRAD):

- M1 Alice generates a cbit i (zero or one) arbitrarily. According to the value of i, Alice creates a corresponding Bell state $\mathbb{B}$ by a mapping: $0 \to |\Phi^+\rangle$ and $1 \to |\Psi^-\rangle$.
- M2 Let λ_A and λ_B denote the first and second qubits of the Bell state $\mathbb{B}$. Alice sends λ_B (called the checking bit) to Bob and retains λ_A.
- M3 Bob reflects the qubit λ_B back to Alice.
- M4 Alice receives the qubit λ_B' and pairs it with λ_A. She performs Bell measurement (Fig. 1) on the pair to get b_1 and b_2.
- M5 The values of b_1 and b_2 determine the Bell state the circuit measured. If the Bell state coincides with the one Alice generated in Step M1, then the protocol is considered secure (represented by zero). Otherwise, there exists an MRA (represented by 1).

Example 1. *Consider Alice and Bob using MRAD to detect MRAs. Assume Alice gets* $i = 0$ *and then creates the corresponding Bell state (denoted by* $\lambda_A \lambda_B = |\Phi^+\rangle = \frac{|00\rangle + |11\rangle}{\sqrt{2}}$*). Eve perpetrates an MRA, by measuring* λ_B *and getting the result* $r = 0$*. At the same moment,* λ_A *collapses to* $|0\rangle$ *because of the entanglement. Eve sends the corresponding qubit (*$|0\rangle$*) to Bob, which is reflected back to Alice.*

Alice performs a Bell measurement on the qubit pair consisting of λ_A *and the received* $|0\rangle$*. Since* λ_A *has collapsed to* $|0\rangle$*, the input to the circuit is* $\lambda_A \lambda_B = |00\rangle$*. According to Table 1, there is* 50% *probability of getting* $b_1 b_2 = 00$ *(conclude that the input is* $|\Phi^+\rangle$ *according to the criterion in Section 2.1, a false negative) as well as to get* $b_1 b_2 = 10$ *(conclude that the input is* $|\Phi^-\rangle$*, a true positive).*

From the example, if Alice gets $i = 0$ and Eve obtains the measurement result $r = 0$, then Alice has 50% probability of concluding that there is no MRA, though it was performed. The same conclusions are drawn for all the combinations of i and r according to Table 1. Therefore, we get the following lemma and theorem.

Lemma 1. *Given that the checking bit is attacked, the probability that MRAD detects an MRA is* 50% .

Theorem 1. *If Alice and Bob repeat MRAD n times, then there is* $1 - \left(\frac{1}{2}\right)^n$ *probability of detecting MRAs given that n checking bits are attacked.*

Proof. $P[\text{detect MRAs}] = 1 - P[\text{MRAD fails}]^n = 1 - \left(\frac{1}{2}\right)^n$ □

Remark 1. *MRAD essentially checks whether the checking bits sent by Alice are measured by anybody else, but it cannot tell who measures it. It can detect MRAs only when Alice knows Bob does not measure any checking bits. Therefore, if there is any measurement detected, it must be an attack.*

Since we apply the mapping rule in Step M1 and the operation in Step M5 again in the later discussion, we define them as follows.

Definition 1 (Mapping to Bell states (F), Step 1)**.** *Given a cbit i, function* $F:\{0,1\}\rightarrow\{|\Phi^+\rangle,|\Psi^-\rangle\}$ *is defined by:* $F(0)=|\Phi^+\rangle$*, and* $F(1)=|\Psi^-\rangle$*.*

Remark 2. *Notice that F is bijective. Therefore, given the output sequence consisting of instances* $|\Phi^+\rangle$ *and* $|\Psi^-\rangle$*, we can use* F^{-1} *to get the original qubit sequence I.*

Definition 2 (Alice Verifies (AV), Step 5)**.** *Given* $b_1,b_2,i\in\{0,1\}$*, we define function AV*$(b_1,b_2,i):\{0,1\}^3\rightarrow\{0,1\}$ *by:*

$$AV(b_1,b_2,i)=\begin{cases}0: \text{if } b_1=b_2=i\\ 1: \text{otherwise}\end{cases}$$

Note that $0\rightarrow$ *no attack detected, and* $1\rightarrow$ *attack detected.*

3 New Protocol

In this section, we propose a new SQDC protocol called Economic No-Key Semi-Quantum Direct Communication (ENKSQDC). We start the discussion with an introduction to a data transmission protocol called SBP, which is a building block of ENKSQDC (not self-contained). Assuming that there are no MRAs, we show that SBP is secure (Theorem 2). In the design of SBP, Bell measurement seems redundant. It is intended for sharing the quantum circuit with MRAD (Remark 3). The considerable benefits of this design are discussed in Section 5. To meet the assumption of Theorem 2, we integrate MRAD and SBP to get ENKSQDC. If we assume that ENKSQDC detects all MRAs, then it is provably secure (Theorem 4).

3.1 Single-Bit Protocol (SBP)

Protocol 1 (SBP)**.** *Alice sends a single cbit* $d\in\{0,1\}$ *to Bob. The protocol needs a bidirectional quantum channel and a bidirectional public classical channel. It runs in the following way:*

S1 Alice generates a random cbit i and then set a Bell state $\mathbb{B}=F(i)$ *(Definition 1). Let* λ_A *denote the first qubit of* $\mathbb{B}$ *and* λ_B *the second.*

S2 Alice retains λ_A *and sends* λ_B *to Bob.*

S3 Bob measures the qubit λ_B *in the Z-basis to obtain a cbit* r_B *(at the same moment,* λ_A *collapses due to the entanglement with* λ_B*). Simultaneously, he sends a pre-generated qubit* $\lambda_B^*=|0\rangle$ *back to Alice and notifies Alice that* λ_B *has been measured through the classical channel.*

S4 Alice performs a Bell measurement on the qubit pair $|\lambda_A\lambda_B^*\rangle=|\lambda_A 0\rangle$ *(*λ_A *is retained in Step S1) and obtains* b_1 *and* b_2*.*

S5 According to Table 1, if $b_1b_2=00$ *or* 10*, then* λ_A *was* $|0\rangle$*. If* $b_1b_2=01$ *or* 11*, then* λ_A *was* $|1\rangle$*. Pairing* λ_A *with the Bell state* $\mathbb{B}$ *she chose (recorded by i) in Step S1, she determines the value of* r_B *measured by Bob in Step S3. In particular, she uses the following rule:*

$$i=0\Rightarrow|\Phi^+\rangle=\frac{|00\rangle+|11\rangle}{\sqrt{2}}\Rightarrow\begin{cases}\text{if}\lambda_A=|0\rangle, r_B=0\\ \text{if}\lambda_A=|1\rangle, r_B=1\end{cases}$$

$$i=1\Rightarrow|\Psi^-\rangle=\frac{|01\rangle-|10\rangle}{\sqrt{2}}\Rightarrow\begin{cases}\text{if}\lambda_A=|0\rangle, r_B=1\\ \text{if}\lambda_A=|1\rangle, r_B=0\end{cases}$$

S6 If $r_B=d$*, Alice notifies Bob, through the classical channel, that* r_B *is the right value. Otherwise, Alice notifies Bob to take the complement of* r_B*.*

Remark 3. *The pre-generated qubit* $\lambda_B^*=|0\rangle$ *in Step S3 is unnecessary to implement SBP. So is the Bell measurement in Step S4. In fact, in Step S3, Bob only needs to notify Alice that he has measured* λ_B*, and, in Step S4, Alice simply uses the Z-basis to get the value of* λ_A*. Here, we intendedly implement SBP with redundant operations so that the new protocol (ENKSQDC, introduced in Section 3.2) can use one fixed quantum circuit to implement both the attack detection (MRAD) and data transmission (SBP) protocols. We discuss the design and its benefits in details in Section 3.2, and more performance analysis is made in Section 5.*

In the sequel, we use the operations introduced in Steps S5 and S6. We refer to them as a function and an operation. In Step S5, Alice determines r_B owned by Bob without communication. We call this function Tele-Conjure.

Definition 3 (Tele-Conjure). *Given b_1, b_2 and i (Step S1), function Tele-Conjure $TC(b_1, b_2, i) \to \{0, 1\}$ returns the value of r_B according to Step S5.*

Besides, for the operation in Step S6, Alice corrects Bob's measurement results. Thence, we call this operation Correct.

Operation 1 (Correct). *According to the value of r_B (obtained in Step S5) and a single bit message d, Alice sends Bob a confirmation, including either right or wrong, through the classical channel. If Bob receives right, then r_B is the message Alice sends. Otherwise, it is the complement of r_B.*

Theorem 2 is about the security of SBP.

Theorem 2. *SBP is secure if Bob can receive the qubit λ_B from Alice without MRA.*

Proof. We assume that there is no MRA. So, we essentially assume that Steps S1 to S3 are always secure (Bob only sends a confirmation in Step S3). There is no communication in Step S4 and S5. In the last step, Alice sends a confirmation irrelevant to the value of datum d, which implies Step S6 is also secure. Therefore, we conclude that SBP is secure. □

Remark 4. *Theorem 2 depends on the assumption that the classical channel is authentic. While Alice and Bob exchange information, it is necessary to check that they are truly communicating with each other and their public messages are not modified. In other words, they need resistance to man-in-the-middle attacks.*

According to Theorem 2, we can say SBP is secure only if there is no MRA, however, which cannot be detected by SBP itself. Thence, a corresponding detection mechanism should be added. In particular, if we use MRAD to guarantee there is no MRA, then we can use SBP to transmit data securely according to Theorem 2. With this preliminary idea in mind, we derive the following protocol:

3.2 Economic No-Key Semi-Quantum Direct Communication (ENKSQDC)

We use the hardware of SBP. Specifically, Alice and Bob are connected by a quantum bidirectional channel and a public classical bidirectional channel. We define four operations requiring quantum resources in the new protocol.

Operation 2 (Alice sends). *Suppose that this is the kth transmission by Alice. Alice randomly generates a cbit and stores it in a register i_k. Alice generates a Bell state $F(i)$, retains its first qubit λ_{kA} and sends the second λ_{kB} to Bob.*

Operation 3 (Bob measures). *When Bob receives the kth qubit sent by Alice, he measures it to get s_k. Simultaneously, he sends a pre-generated qubit $|0\rangle$ to Alice. Moreover, he records the fact that the kth qubit he received is measured.*

Operation 4 (Bob reflects). *Bob reflects the kth qubit sent by Alice back without measurement. Besides, he also records the fact that the kth qubit he received is reflected.*

Operation 5 (Alice measures). *For the kth qubit λ^*_{kB} received by Alice, Alice applies Bell measurement on $|\lambda_{kA}\lambda^*_{kB}\rangle$ (Alice retains λ_{kA} in Step E1 while applying Operation 2) and stores the result as $b_{1k}b_{2k}$.*

Protocol 2 (ENKSQDC). *Suppose Alice sends a message $\underset{\sim}{G} = g_1 g_2 \cdots g_m$ of length m to Bob. Besides, extra r bits are used for detecting MRAs. The protocol consists of following the steps:*

E1 *Alice performs Operation 2 for $m+r$ times and stores i_k for $k = 1, 2, \ldots, m+r$, in string $\underset{\sim}{I} = i_1 i_2 \cdots i_{m+r}$.*

E2 *For the $m+r$ qubits sent by Alice, Bob randomly chooses m qubits to perform Operation 3 and remaining r qubits to perform Operation 4. Bob puts all the measurement results s_k for $k = 1, 2, \ldots, m$ obtained from Operation 3 into a new string $\underset{\sim}{S} = s_1 s_2 \cdots s_m$ (after reindexing but not changing the order)*

E3 *For the $m+r$ qubits sent back from Bob, Alice performs Operation 5 and stores $b_{1k}b_{2k}$ in two strings $\underset{\sim}{B_1} = b_{11}b_{12} \cdots b_{1(m+n)}$ and $\underset{\sim}{B_2} = b_{21}b_{22} \cdots b_{2(m+n)}$, respectively.*

E4 *Through the classical channel, Bob notifies Alice of the qubits reflected and measured in Step 2 by sending a binary string $\underset{\sim}{P} = p_1 p_2 \cdots p_{m+r}$. For $k = 1, 2, \cdots, m+r$, $p_k = 0$ indicates that the kth qubit is reflected while $p_k = 1$ means it is measured.*

E5 *After receiving $\underset{\sim}{P}$ from Bob, for $k = 1, 2 \cdots, m+r$, if $p_k = 0$, Alice applies function $AV(b_{1k}, b_{2k}, i_k)$ in Definition 2. If the result is 1, then it means that the kth qubit Alice sent to Bob is measured by Eve (MRA). Thence, the security of the protocol cannot be guaranteed. Alice and Bob terminate the protocol. If $p_k = 1$, Alice applies function $TC(b_{1k}, b_{2k}, i_k)$ in Definition 3 and stores the function's value c_k. Note that c_k equals the s_k in Step E2 before reindexing.*

E6 *Since Bob measures m qubits, then if Alice puts all c_k's in one string, she has $\underset{\sim}{C} = c_1c_2\cdots c_m$ (after reindexing in the original order). Note that $\underset{\sim}{C}$ equals $\underset{\sim}{S}$, which is owned by Bob.*

E7 *For $k = 1, 2, \cdots, m$, Alice and Bob perform Operation 1 with inputs g_k, c_k and s_k. Then Bob gets the message from Alice.*

It is worth noting that, Steps E1 to E3 are not performed sequentially but in parallel. Because of this design, Alice only needs a small constant number of quregisters and preserves entanglement for a short and fixed length of time (we discuss this highlight in details in Section 5.1).

The protocol essentially lets Alice and Bob share a random m-bit binary string (Steps E1 to E6) $\underset{\sim}{S}$ $(= \underset{\sim}{C})$, which can be considered as a shared key too. In other words, the protocol can be considered as a key distribution protocol between a full quantum capability sender and a classical receiver. With the shared binary string, Alice then can send a message to Bob by performing Operation 1. To amortize the shared key establishment cost, Alice and Bob can repeat Step E7 to transfer several messages before updating the shared string $\underset{\sim}{S}$ $(= \underset{\sim}{C})$ (by repeating Steps E1 to E6).

The ENKSQDC protocol is a hybrid of SBP and MRAD. The operations of Alice in the first four steps are exactly the same (see Figure 2). Since all the quantum operations of SBP and MRAD are performed in the first four steps, Alice can use a single quantum circuit to complete the first four steps of the two protocols without knowing which one she is actually following. In Figure 2, we put the first four steps of SBP and MRAD together and label the operations with the step numbers used to introduce them. Besides, we also provide the corresponding step numbers of the ENKSQDC protocol.

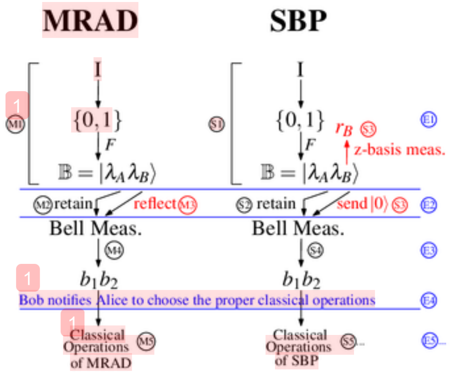

Figure 2. The relationships between ENKSQDC, SBP and MRAD. The actions in red are made by Bob, while the Black ones are made by Alice. The step numbers in blue are the corresponding actions in ENKSQDC. In SBP and MRAD, quantum operations (first four steps) are quite similar except Bob's treatment on the qubits sent by Alice.

We can observe that the only difference between SBP and MRAD is the operation made by Bob. In other words, Bob decides which protocol is being implemented. Specifically, Bob decides the protocol being applied by either measuring λ_{kB} (sending a pre-generated $|0\rangle$ simultaneously) or reflecting it. Note that the pre-generation of the $|0\rangle$, instead of generating it on demand, secures ENKSQDC against the delay and reflection attacks [11]. If λ_{kB} is measured, then it is a data bit for delivering messages (implementing SBP); otherwise, it is a checking bit for detecting MRAs (implementing MRAD).

After Alice and Bob complete the first three steps in ENKSQDC, Bob sends a message through the classical channel to notify Alice of the qubits either he measured or reflected. According to the message, Alice chooses the proper classical operations either to detect MRAs (MRAD) or to determine the measurement results of Bob (SBP).

Remark 5. *Since Bob determines the protocol being implemented by choosing Measure or Reflect, then in ENKSQDC, Bob chooses Measure m times and Reflect r times; therefore, SBP and MRAD are run m and r times respectively.*

3.3 Security analysis of ENKSQDC

The ENKSQDC and SBP protocols share the same assumption – the public channel must be authentic for the same reason (see Remark 4). According to Remark 5, ENKSQDC can be thought as a combination of m SBPs and r MRADs. MRAD is used to detect MRAs. Thence, it secures SBP (see Theorem 2). According to Theorem 1, the probability of detecting MRAs is $1 - \left(\frac{1}{2}\right)^n$, where n is the number of MRAD applied. Therefore, in the ENKSQDC protocol, since MRAD is performed r times, the detection success rate is $1 - \left(\frac{1}{2}\right)^r$ when Eve perpetrates MRAs on all qubits sent by Alice. Assuming that there is a specific probability that the adversary Eve attacks the qubits sent by Alice, we have the following theorem.

Theorem 3. *Let us assume that in the ENKSQDC protocol, Alice sends $m+r$ qubits to Bob and for each qubit, the adversary Eve perpetrates MRAs with probability p. Bob measures m qubits and reflects r qubits (checking bits). Then the probability that Alice detects an MRA is $1-\left(1-\frac{p}{2}\right)^r$.*

Proof. Let N denote the number of checking bits (that is, the bits reflected by Bob) attacked by Eve. Since for each checking bit, Eve has probability p of attacking it; therefore, N follows a binomial distribution with the number of experiments r and success rate p. Let A be an indicator of the detection result. $A=1$ means there is an attack detected while $A=0$ means no attack detected. Then the probability that Alice detects an MRA is the expectation of A. So we have,

$$
\begin{aligned}
E[A] &= 0\cdot P[A=0]+1\cdot P[A=1]=P[A=1] \\
&= \sum_{k=0}^{r} P[A=1|N=k]P[N=k] && \text{(Law of total probability)} \\
&= \sum_{k=0}^{r}\left(1-\left(\frac{1}{2}\right)^k\right)\binom{r}{k}p^k(1-p)^{r-k} && \text{(by Theorem 1)} \\
&= \sum_{k=0}^{r}\binom{r}{k}p^k(1-p)^{r-k}-\sum_{k=0}^{r}\left(\frac{1}{2}\right)^k\binom{r}{k}p^k(1-p)^{r-k} \\
&= 1-\left(\frac{p}{2}+(1-p)\right)^r && \text{(Binomial expansion)} \\
&= 1-\left(1-\frac{p}{2}\right)^r
\end{aligned}
$$

□

It is worth noting that if we let $p=1$, then Eve measures all the qubits sent by Alice. The probability that Alice detects an attack is $E(A)=1-\left(1-\frac{p}{2}\right)^r=1-\left(1-\frac{1}{2}\right)^r=1-\left(\frac{1}{2}\right)^r$, which is consistent with the observation at the beginning of Section 3.3.

Figure 3 plots the detection success rate according to the function given in Theorem 3 where r ranges from zero to 15 with different probabilities p. We also add our simulation results (the points) in the figure for comparison. The points on a curve of a given color have the same probability p, indicated in the legend. The simulation results and Theorem 3 are consistent. We can observe that the detection success rate converges to one faster with a larger p. The observation makes sense since a higher attack rate implies that more checking bits are affected on average, which results in an increase of the detection success rate. A similar conclusion can be drawn if we increase the number of checking bits, that is, variable r.

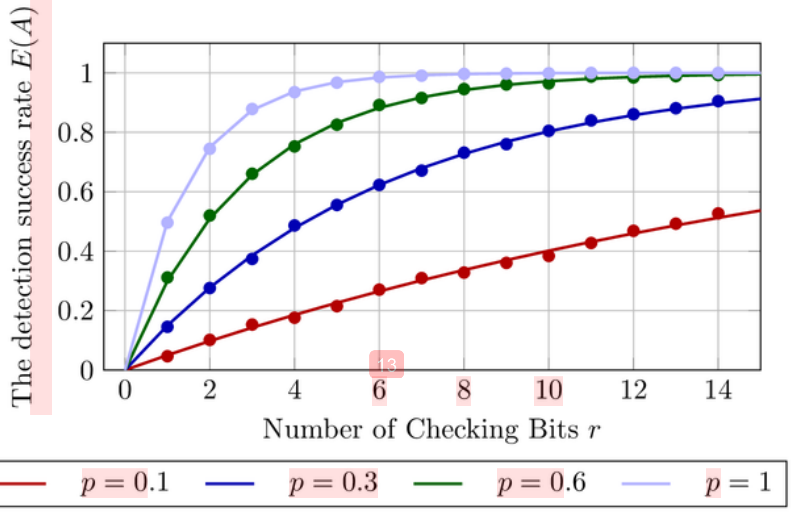

Figure 3. The detection success rate $E(A)$ in terms of the number of checking bits r by choosing different attack probability p.

Assuming that MRAs are always detectable, then we can conclude that the ENKSQDC protocol is secure.

Theorem 4. *Given that MRAs are always detectable, the ENKSQDC protocol is secure.*

Proof. When an attack is detected, Alice and Bob abort the transmission. The message is secure. Otherwise, according to the

assumption, there is no MRA. Remark 5 states that the protocol essentially implements SBP m times for transmitting an m-bit message from Alice to Bob. Combined with Theorem 2, the ENKSQDC protocol is secure against any network attack. □

4 Rate Estimation ENSQDC (RENKSQDC)

ENKSQDC detects MRAs and is secure assuming no hardware fault nor environmental disturbance that destroy entanglement. So far, we ignored them for the sake of simplicity. They do exist in practice. Ignoring them produces false positives and incorrect protocol terminations. In this section, we enhance the detection part of the protocol to fix this issue. Destructions of entanglement involving checking bits may result in Positive MRADs (PMs) whose probability is denoted by ρ and estimated by its rate

$$\hat{\rho} := \frac{\text{Number of PMs}}{\text{Number of checking bits}}.$$

The destructions have two types. In particular, we say that a qubit is disturbed if the entanglement involving it is destroyed due to a hardware imperfection or an environmental disturbance. If the destruction of the entanglement is caused by an eavesdropper Eve, we say the qubit is attacked. We show that two times $\hat{\rho}$ is an estimator $\hat{\kappa}$ of the probability κ that a checking bit is disturbed or attacked. Let ω denote the probability that a qubit is disturbed. If ω is unknown, we can estimate it ahead of the protocol execution assuming that Eve does not perpetrate attacks. As no qubits are attacked during the estimation, κ is reduced to ω. Correspondingly, $\hat{\kappa}$ is reduced to $\hat{\omega}$, an estimator of ω. During the execution of the protocol, the attacks perpetrated by Eve increase κ and cause its deviation from ω. By monitoring the difference between κ and ω, we gauge the existence of attacks and thus the security of the protocol. We use the following symbols and facts for the statistical analysis in the sequel. Let $B(n,p)$ be a binomial distribution with $n \in \mathbb{N}$ trials and success rate $p \in [0,1]$, $N(\mu,\sigma^2)$ a normal distribution with mean $\mu \in \mathbb{R}$ and variance σ^2 and $\bar{X}$ the arithmetic mean of X.

Remark 6. *We call* $B(1,p)$ *a Bernoulli distribution with the success rate* p.

Remark 7. *Random Variables of binomial distributions can be added if they have the same success rate. In particular, if* $X \sim B(n,p)$ *and* $Y \sim B(m,p)$*, then* $X+Y \sim B(n+m,p)$ *[18].*

Fact 1. *Suppose* $X \sim N(\mu_X,\sigma_X^2)$*. Then* $\frac{X-\mu_X}{\sigma_X}$ *follows a standard normal distribution. Namely,* $\frac{X-\mu_X}{\sigma_X} \sim N(0,1)$.

Fact 2. *Suppose* $X \sim N(\mu_X,\sigma_X^2)$ *and* $n \in \mathbb{R}^+$*. Then,* $\frac{X}{n} \sim N(\frac{\mu_X}{n},\frac{\sigma_X^2}{n^2})$.

Fact 3. *Suppose* $X \sim N(\mu_X,\sigma_X^2)$ *and* $Y \sim N(\mu_Y,\sigma_Y^2)$ *are independent. Then,* $X-Y \sim N(\mu_X-\mu_Y,\sigma_X^2+\sigma_Y^2)$.

Theorem 5 discusses the random processes in the detection of disturbed and attacked qubits.

Theorem 5. *Suppose that in the ENKSQDC protocol, Bob reflects* r *qubits. Let* $D_i \in \{0,1\}$ *denote a r.v. of the detection result* d_i *of the* i^{th} *MRAD such that:*

$$d_i = \begin{cases} 1 & \text{if the } i^{th} \text{ MRAD has a positive detections} \\ 0 & \text{otherwise} \end{cases}$$

Then D_i*'s are independent and identically distributed (iid)* $B(1,\rho)$*. Or in short,* $D_i \overset{iid}{\sim} B(1,\rho)$ *. The number of PMs (denoted by* C_ρ*) is* $\sum_{i=1}^{r} D_i$*, which is a binomial distribution* $B(r,\rho)$*. Moreover,* $\rho = \frac{\kappa}{2}$.

Proof. Since ρ is the probability of positive detection and all MRADs are mutually independent, $D_i \overset{\text{iid}}{\sim} B(1,\rho)$ for $i = 1\cdots r$. Then the number of PMs $C_\rho = \sum_{i=1}^{n} D_i$. By Remark 7, we have $C_\rho \sim B(r,\rho)$. Let A_i be a r.v. such that, if the checking bit of ith MRAD is disturbed or attacked, then $A_i = 1$; otherwise, $A_i = 0$. So we have, $P(A_i = 0) = 1-\kappa$ and $P(A_i = 1) = \kappa$. According to Lemma 1, if the checking bit is disturbed or attacked, the probability of a positive detection is $P[D_i = 1|A_i = 1] = \frac{1}{2}$. Otherwise, the checking bit is intact which implies that the result must be negative. Namely, $P[D_i = 1|A_i = 0] = 0$. By Law of total probability,

$$\rho = P[D_i = 1] = P[D_i = 1|A_i = 1] \cdot P[A_i = 1] + P[D_i = 1|A_i = 0] \cdot P[A_i = 0] = \frac{1}{2} \cdot \kappa + 0 \cdot (1-\kappa) = \frac{\kappa}{2}.$$

□

Remark 8. *A binomial distribution* $N(n,p)$ *has mean* np *and variance* $np(1-p)$*. So the binomial distribution* $C_\rho \sim B(r,\rho)$ *in Theorem 5 has mean* $r\rho = \frac{\kappa}{2}r$ *and variance* $r\rho(1-\rho) = \frac{1}{2}r\kappa(1-\frac{\kappa}{2})$ *[18].*

Remark 9. *A binomial distribution $B(n,p)$ can be approximated by a normal distribution with the same mean and variance if $n \geq max\left\{\frac{45(1-2p)^2}{p(1-p)}, \frac{14|1-6p(1-p)|}{p(1-p)}\right\}$ [19]. Therefore, the binomial distribution $C_\rho \sim B(r, \frac{\kappa}{2})$ in Theorem 5 has a normal approximation $N(\frac{\kappa}{2}r, \frac{1}{2}r\kappa(1-\frac{\kappa}{2}))$ if $r \geq max\left\{\frac{180(1-\kappa)^2}{p(2-\kappa)}, \frac{56|1-3\kappa(1-0.5\kappa)|}{\kappa(2-\kappa)}\right\}$.*

Theorem 6 provides a method to estimate the parameter p of a Bernoulli distribution [18].

Theorem 6. *Suppose that $X_i \overset{iid}{\sim} B(1,p)$ for $i \in \{1,2,\cdots,n\}$. Then $\hat{p} = \bar{X} = \frac{\sum_{i=1}^n x_i}{n}$, an unbiased estimator of p.*

By Theorem 6, $\rho = \frac{\kappa}{2}$ has an unbiased estimator $\hat{\rho} = \widehat{\kappa/2} = \frac{\sum_{i=1}^r D_i}{r} = \frac{C_\rho}{r}$. Therefore, κ can be estimated by

$$\hat{\kappa} = \frac{2C_\rho}{r}\,. \tag{1}$$

Remark 9 states that $C_\rho = \sum_{i=1}^r D_i \sim B(r, \frac{\kappa}{2})$ approximately follows a normal distribution $N\left(\frac{\kappa r}{2}, \frac{1}{2}r\kappa(1-\frac{\kappa}{2})\right)$. Combining with Fact 2, we conclude that $\widehat{\kappa/2} = \frac{C_\rho}{r} \sim N\left(\frac{\kappa}{2}, \frac{\kappa(1-\frac{\kappa}{2})}{2r}\right)$. Applying Fact 2 again, we have $\widehat{\kappa} \sim N\left(\kappa, \frac{2\kappa(1-\frac{\kappa}{2})}{r}\right)$.

4.1 Rate difference monitoring

When ω is unknown, we need to estimate it before starting the execution of the protocol. We have to assume that during this estimation, there is no attack. Under this assumption, κ is reduced to ω, the probability that a checking bit is disturbed. Correspondingly, $\hat{\kappa}$ is reduced to an estimator of ω. Namely, $\kappa = \omega$ and $\hat{\kappa} = \hat{\omega}$. As we have shown $\widehat{\kappa} \sim N\left(\kappa, \frac{2\kappa(1-\frac{\kappa}{2})}{r}\right)$, we also have $\hat{\omega} \sim N\left(\omega, \frac{2\omega(1-\frac{\omega}{2})}{s}\right)$, where s is the number of checking bits for estimating ω. Let C'_ρ denote the number of PMs under the assumption that the checking bits are not attacked. By replacing C_ρ by C'_ρ and s by r in equation (1), we get,

$$\hat{\omega} = \frac{2C'_\rho}{s}\,. \tag{2}$$

In RENKSQDC, the attack detection method is implemented by checking that $\kappa = \omega$. After getting the estimations of κ and ω, let e denote their difference, which is an outcome of r.v. $E = \hat{\kappa} - \hat{\omega}$. Fact 3 states that E still follows a normal distribution. In particular, $E \sim N\left(\kappa - \omega, \frac{2\kappa(1-\frac{\kappa}{2})}{r} + \frac{2\omega(1-\frac{\omega}{2})}{s}\right)$. Under the null hypothesis H_0 that there is no attack, $\kappa = \omega$. Then $E \sim N\left(0, 2\nu\left(1-\frac{1}{2}\nu\right)\left(\frac{1}{r}+\frac{1}{s}\right)\right)$, where $\nu = \kappa = \omega$ and can be estimated by $\hat{\nu} = \frac{2(C'_\rho + C_\rho)}{r+s}$. So, if H_0 is true, the distribution of r.v. E is condensed near zero. Although the set of the possible outcomes of E is $\mathbb{R}$, the test can rule out outcomes that are much greater than zero without introducing much error (note that we do not consider a negative difference because κ is, theoretically, not less than ω. In other words, the alternative hypothesis H_1 is $\kappa > \omega$). Let α denote the probability that an outcome of E is much greater than zero and ruled out by the test. We can test H_0 against H_1 by rejecting H_0 if we observe an outcome of E greater than e_α, where $e_\alpha \in \mathbb{R}$ such that $P(E > e_\alpha) = \alpha$. In other words, the protocol is considered insecure if e, the difference between the estimations of κ and ω, is greater than e_α.

The arduous calculation of e_α can be avoided if we scale E to

$$Z = \frac{E - 0}{\sqrt{2\nu\left(1-\frac{1}{2}\nu\right)\left(\frac{1}{r}+\frac{1}{s}\right)}} = \frac{\hat{\kappa} - \hat{\omega}}{\sqrt{2\hat{\nu}\left(1-\frac{1}{2}\hat{\nu}\right)\left(\frac{1}{r}+\frac{1}{s}\right)}}, \tag{3}$$

a standard normal distribution according to Fact 1. So correspondingly, the difference e after scaling (denoted by z) is an outcome of Z. Then an equivalent test can be made by rejecting H_0 if $z > z_\alpha$ where $z_\alpha \in \mathbb{R}$ such that $P(Z > z_\alpha) = \alpha$. The table listing the value of z_α as a function of α can be found in Reference [18]. Therefore, we amend the original ENKSQDC protocol as follows:

Protocol 3 (RENKSQDC)**.**

RE1 *(Estimation of ω) Alice and Bob execute MRAD s times. Alice sends s qubits to Bob. He reflects all of them. In other words, there are s checking bits and zero data bits. In Step E5, Alice counts the number of PMs (denoted by C'_ρ). Finally, she uses equation (2) to estimate ω. Note that during the estimation process, we need to guarantee that Eve does not perpetrate attacks.*

RE2 *Alice and Bob start the execution of the protocol. They do Steps E1-E4.*

RE3 *In E5, instead of terminating the protocol when $p_k = 0$ and function $AV(b_{1k}, b_{2k}, i_k) = 1$, Alice increments a counter C_ρ (initial value is zero) and continues to check the remaining bits of $\underset{\sim}{P}$. After finishing checking, she uses equation (1) to estimate κ. We test the null hypothesis $H_0 : \kappa = \omega$ against the alternative hypothesis $H_1 : \kappa > \omega$, equation (3). If H_0 is rejected, Alice considers the protocol is insecure and terminates it; otherwise, Alice and Bob execute Steps E6 and E7 to complete the data transmission.*

When ω is given, we can simply compare it with the estimation of κ. Similarly, we need to test $H_0 : \kappa = \omega$ against $H_1 : \kappa > \omega$. Since ω is not estimated but a given constant, we can say $\hat{\omega} \sim N(\omega, 0)$. We estimate the real attack rate κ by equation (1). Applying Fact 3, we have that $E = \hat{\kappa} - \omega = \hat{\kappa} - \hat{\omega} \sim N\left(\kappa - \omega, \frac{2\kappa(1-\frac{\kappa}{2})}{r}\right)$. Under the assumption that H_0 is true, $E \sim N\left(0, \frac{2\kappa(1-\frac{\kappa}{2})}{r}\right)$. Applying Fact 1, we scale E to $Z' = \frac{(\hat{\kappa}-\omega)-0}{\sqrt{\frac{2\kappa(1-\frac{\kappa}{2})}{r}}} = \frac{\hat{\kappa}-\omega}{\sqrt{\frac{2\kappa(1-\frac{\kappa}{2})}{r}}} \sim N(0,1)$. Let z' denote the scaled difference of the estimated κ and the pre-known ω, which is an outcome of Z'. We reject H_0 if $z' > z_\alpha$, where the definition of z_α is unchanged.

Since ω is given, its estimation is unnecessary. To complete the data transmission, Alice and Bob only need to implement Steps RE2 and RE3, where Z is replaced by Z'.

4.2 Security analysis of RENKSQDC

As we have mentioned at the beginning of this section, the original MRAD fails if the qubits transmitted are disturbed and the entanglement is destroyed. The false positives mislead the protocol about the transmission security and cause wrong termination. To fix the problem, in Sections 4.1, we propose a new detection method for MRAs based on a statistical test. The method detects the discrepancy between ω and κ, which does not exist if there is no attack. If any significant discrepancy is identified, the protocol is considered insecure and terminated.

The test rules out the possible outcomes of E that are largely greater than zero, and thus, introduces detection errors. In more details, suppose that Eve does not perpetrate attacks, which implies $\kappa = \omega$ and the null hypothesis H_0 is true. Due to the fluctuation of the estimator D, the difference between $\hat{\kappa}$ and $\hat{\omega}$, there is a proabability α that the sampling of D is greater than the threshold d_α and gets the H_0 rejected, which is a false positive. Correspondingly, if Eve perpetrates attacks and causes $\kappa > \omega$, it is also possible that H_0 is not rejected since their difference is still less than d_α, which is a false negative. We formally define these two types of errors as follows,

Definition 4 (Type A Error - False Negative)**.** *Eve perpetrates an attack, but the protocol is wrongly considered secure.*

Definition 5 (Type B Error - False Positive)**.** *Eve does not perpetrate an attack, but the protocol is wrongly considered insecure.*

The Type A Error has more adverse consequences than the Type B Error because Eve can eavesdrop the message without the awareness of Alice and Bob. We show that the probability of undetected eavesdropping is very low, even when a small number of checking bits is used. The Type B error does not undermine the security of the protocol. Instead, it lowers the transmission efficiency. While Eve does not perpetrate an attack, the Type B error causes a wrong belief of its presence and a termination of the protocol. The protocol needs to restart and resend all qubits. The transmission efficiency is affected.

The choice of a specific value for α, the occurrence probability of the Type B Error, affects the one of the Type A Error. In particular, an increase of α pushes the value of d_α to zero. Although Eve only attacks a few portion of the checking bits, the difference between κ and ω she introduces may still exceed the lowered d_α and get H_0 rejected; therefore, the test becomes stricter and the occurrence probability of Type A Error decreases. Similarly, we can show that a decrease of α leads to an increase of Type A Error occurrence probability. Since the Type A and B Error occurrence probabilities have a negative relationship, if we increase α to enhance the security level, we get more Type B Errors and lower transmission efficiency. Conversely, to decrease the overhead, security is undermined.

With the results of simulations, Figure 4 plots the rates of the two types of errors as a function of α. For estimating the Type A Error occurrence probability, we set the probability p for Eve to attack a qubit to 10% for both cases, and the number of checking bits to estimate κ and ω (if unknown) to 600. Note that the configuration here is intended to make the Type A Error occurrence probability more sensitive to the choice of alpha, which is not typical in practical problems. We will talk about how the error occurrence probabilities behave with more common configurations in the sequel. Whether the value ω is known or not, the trends for both types are consistent with our analysis. When ω is given, the probability of the Type A Error is lower because the estimation of ω introduces more variance, which further amplifies the fluctuation of the estimation of the difference $D = \kappa - \omega$. Regarding the Type B Error, we can observe that the rate roughly equals α which makes sense since it is an estimation of it.

Besides the occurrence probability α of the Type B Error, the numbers of checking bits required to estimate ω and κ are also related to the transmission efficiency. A larger number of checking bits contributes to a better estimation, but also has higher overhead. With the results of simulations, Figures 5 and 6 plot the rates of the Type A and B Errors as a function of the

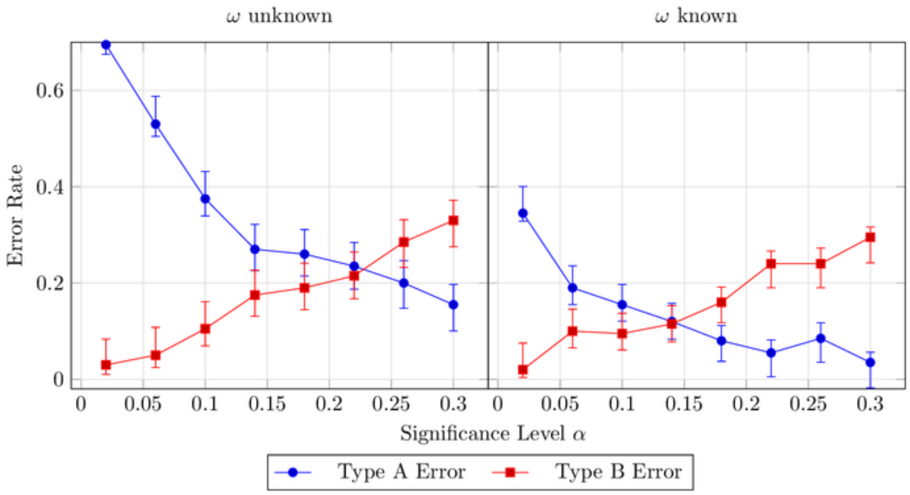

Figure 4. Error rates and their 95% confidence intervals as a function of significance level when the probability ω that a qubit is disturbed is unknown. ($\omega = 0.3$) is unknown (left) and known (right). (Simulation configuration: $r = 600$, $s = 600$ (if ω is unknown), $\omega = 0.3$, $p = 0.1$)

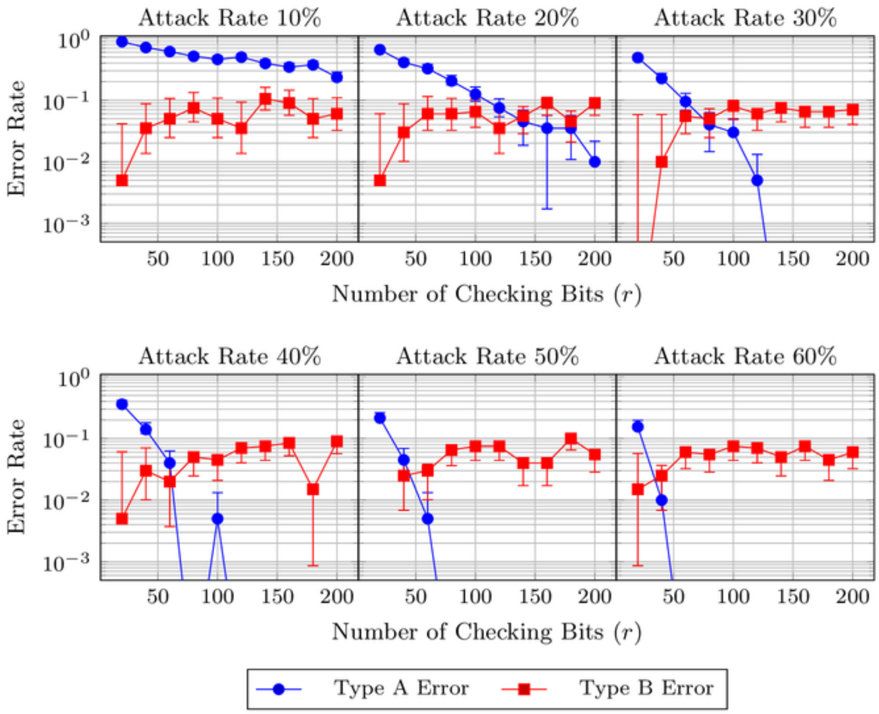

Figure 5. Error rates and their 95% confidence intervals with respect to the number of checking bits when Eve has probability $p = 0.1, 0.2, ..., 0.6$ to attack a qubit. (The probability ω that a qubit is disturbed is unknown. Simulation configuration: $\omega = 0.05$, $\alpha = 0.05$)

number of checking bits and the attack rate. The probability ($\omega = 0.05$) that a qubit is disturbed is unknown in Figure 5 but pre-known in Figure 6. α is set to 0.05. In Figure 5, the numbers of checking bits coincide for the estimation of ω and κ.

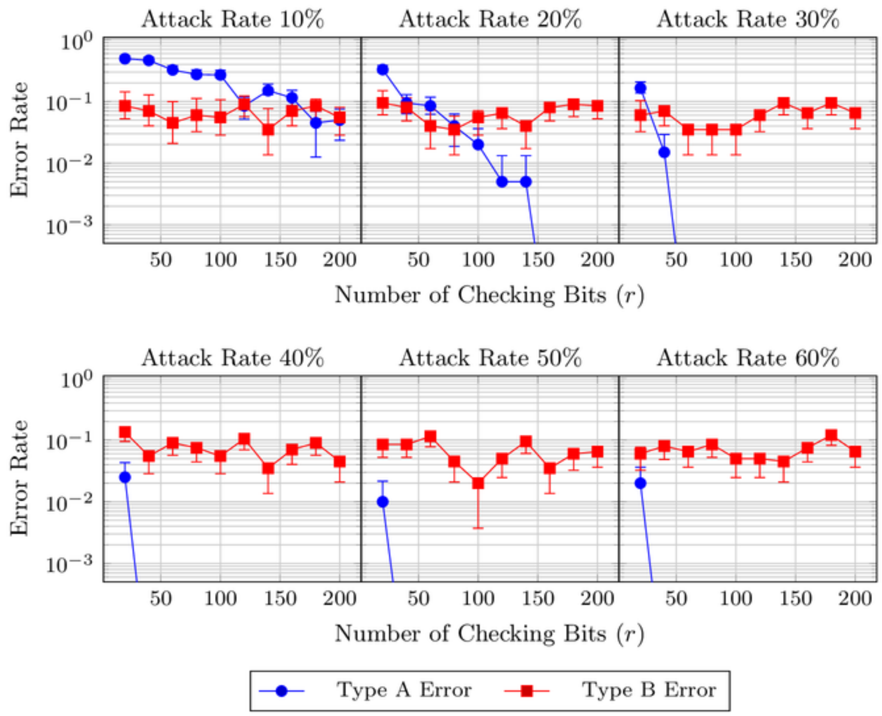

Figure 6. Error rates and their 95% confidence intervals with respect to the number of checking bits when Eve has probability $p = 0.1, 0.2, ..., 0.6$ to attack a qubit. (The probability ω that a qubit is disturbed is known. Simulation configuration: $\omega = 0.05$, $\alpha = 0.05$)

The two figures show that when Eve is more likely to attack a qubit, the detection success rate increases. If Eve only attacks a small proportion of qubits, her attacks do not significantly increase κ and thus are concealed by ω. However, in order to successfully eavesdrop messages, Eve should perpetrates attacks at a rate higher than 50%. When the probability of attacks is 60%, 60 checking bits are sufficient to avoid the Type A Error (when ω is unknown). If ω is given, then 40 checking bits can achieve the same security level.

Note that the Type B Error rate should be constant. In particular, its mean is theoretically equal to 5% as it is an estimation of α. However, while the estimated rate roughly stays around 5% in Figure 6, a relatively considerable increase is observed in Figure 5. The increase is due to a low number of checking bits. According to Remark 9, a good normal approximation requires a large sample size and to estimate both ω and κ, a even larger one is needed. Although, the approximation is not quite accurate when the checking bit number is small, a low level of Type A Error rate shows that it is good enough to secure the protocol.

Since the RENKSQDC and ENKSQDC protocols are the same except for the part that detects MRAs, Theorem 4 is also applicable to RENKSQDC. In particular, we have Theorem 7

Theorem 7. *With a sufficient number of checking bits, the Type A Error can be avoided. So the RENKSQDC protocol is secure.*

5 Quantum Resource Requirements and Transmission Efficiency

We discuss the performance of the ENKSQDC protocol with respect to the quantum resource requirements, qubit efficiency and quantum circuit complexity. Among all SQKD and SQDC protocols, we show that the ENKSQDC protocol has the highest qubit efficiency (almost 100%) with the simplest quantum circuits (without qubits permutation and measurement basis switch). Comparing to the protocols utilizing the quantum entanglements, we show that the ENKSQDC protocol reaches the theoretical minimum of the quregister size and the EPT among the SQKD and SQDC protocols.

5.1 Quantum resources requirements

In Section 3.2, we mentioned that Alice only needs constant quantum resources and fixed EPT to implement the protocol regardless of the length of the message m. In this section, we discuss the quantum resource requirements to implement the

protocol in more details. Since the revised version introduced in Section 4 does not change the hardware requirements, we discuss them together.

Alice and Bob iterate over all i_k's $\in I$ to complete all the quantum operations. Therefore, the minimum quantum resource requirements to implement the protocol is the same as the one to implement a single quantum process QP_k (see Figure 7). To perform Operation 2, Alice uses a Bell state preparation circuit to generate a Bell state. She also needs a single-bit qubit

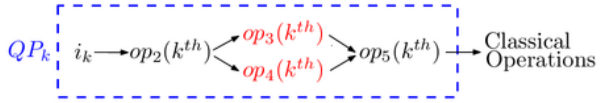

Figure 7. Flow diagram of the first three steps of the ENKSQDC protocol for a single bit i_k. *The operations of Bob are colored in red. For each* QP_k, Bob chooses to perform either $op_3(k^{th})$ or $op_4(k^{th})$.

register to retain its first qubit. Besides, Operation 5 requires Alice to have a Bell measurement circuit. The protocol needs Alice to preserve the entanglement of the Bell states generated in Step E1 until she performs a Bell measurement on it (this is the case when Bob reflects the second qubit λ_B. If Bob measures λ_B, then EPT is shorter). Let us suppose that the time for qubits traveling from Alice to Bob, or the other way, is T. If we ignore the time for Bob to reflect qubits, then the EPT is $2T+P$, where P is the processing time of Alice to send and receive the qubit λ_B. Compared with Alice, Bob only needs the fundamental quantum capability. Specifically, he needs a mechanism to either reflect a qubit or measure it in the Z-basis. To sum up, in order to implement the protocol, the following minimum quantum capabilities are required:

Alice: a single-bit quregister, a Bell state preparation circuit and a Bell measurement circuit.

Bob: a mechanism to either reflect or measure (in the Z-basis) a qubit and a device to generate qubit $|0\rangle$.

Among SQDC and SQKD protocols that utilizes entanglements, Alice must create at least a pair of entangled qubits and send at least one of the qubits to Bob. Therefore, for containing a qubit, a single-bit quregister is necessary. For checking the potential attacks, Alice must do some quantum operations on the qubit pair consisting of the qubit she retained and the one reflected by Bob. So, the EPT is at least $2T+P$. As the ENKSQDC reaches the theoretical lower bound, we conclude that,

Theorem 8. *Among SQDC and SQKD protocols that utilizes entanglements, the ENKSQDC protocol only requires theoretically minimal quregister size and EPT.*

5.2 Transmission efficiency analysis

We assume that Alice and Bob update the shared string $\underset{\sim}{C}$ $(=\underset{\sim}{S})$ for each dialogue. That is, they always go through Step E1 to Step E5 to update the shared binary string before they apply Step E6 and Step E7 to transfer data.

Regarding the original protocol, suppose Alice sends a message of length m to Bob. If Eve attacks qubits with certain probability p, then in order to eavesdrop efficiently, p has to be close enough to 1. We assume $p = 60.0\%$, then if $r = 15$, according to Theorem 3, the detection success rate is higher than 99.5%. Besides, we can reach a much higher security level by adding a few extra checking bits. In usual cases, the length of the message m is supposed to be much greater than 15, which implies that the overhead due to the checking bits becomes negligible, so we will not consider the number of checking bits in the sequel. To transfer m-bit data, Alice needs to send m qubits to Bob, while Bob sends the same number of qubits back. So in total $2m$ qubits are transmitted. If the message is long enough, then the proportion of checking bits is negligible. So the qubit efficiency approaches 100%.

If we consider the possibility that a qubit is disturbed during the qubit transmission, we need to apply the rate estimation version of the protocol. If ω is unknown, we need around 60 checking bits to reach 99% detection success rate (assuming that $\alpha = 0.01$, $\omega = 0.05$), which is acceptable considering a much larger total number of qubits transmitted. If the rate is given, then the checking bits number can decrease to 30 (extra 20 checking bits can improve the success rate to almost 100%). Then the overhead from the checking bits is negligible. The major part of the overhead is from α, the probability to get Type B Error, which causes a full restart of the protocol. In average, $\alpha \cdot 100\%$ qubits transmitted are discarded due to the wrong conclusion that the protocol is insecure. If we choose $\alpha = 0.01$ (which is big enough to secure the protocol), the overhead is only 1%.

Since Alice always performs Bell measurements, there is no need for her to have a capability to switch measurement bases. Moreover, the operations associated with one qubit transmitted by Alice are independent of the ones associated with the others (because Alice does not need to encrypt the message by a permutation, and Bob does not need to apply a reverse permutation to get the original data). Hence, if there is a single qubit transmission or measurement failure, only the operations related to the qubit are required to be re-performed, which potentially increases the transmission success rate and transmission efficiency.

In Table 2, we make a detailed comparison with other typical SQKD and SQDC protocols. Note that the qubit efficiency

(η) is calculated by

$$\eta = \frac{\text{Length of the message}}{\text{Number of qubits sent by Alice}}.$$

For the protocols in References [7–9, 20], η depends on some parameters other than the length of the message n. For these protocols, we give an upper bound for η. Regarding RENKSQDC, η is calculated by choosing $\alpha = 0.01$. Besides, in the protocol proposed by Li et al. [9], the measurement basis switch is not required of Alice or Bob but delegated to a third full quantum capability computer Charlie.

6 Conclusion

A new SQDC protocol (called Economic No-Key Semi-Quantum Direct Communication) has been introduced in this paper. Compared to other SQDC and SQKD protocols, our new protocol has much higher qubit efficiency (almost 100%) and simpler quantum circuits (not requiring switching measurement basis or permuting qubits). While other SQKD and SQDC protocols encrypting messages through entanglements requires at least linear EPT and linear size quregister, in our protocol, only Alice needs to have a constant size quregister (as low as one qubit) and maintain the entanglement of a pair of qubits for time $2T + C$, where T is the time required by Alice and Bob to transmit a qubit to each other, and C is the one consumed by Alice to prepare, receive and measure the qubits.

Among the protocols relying on quantum entanglements to encrypt messages, we show that both quregister size and EPT achieve the theoretical minimums. The new protocol does not need a pre-shared key. Instead, it uses the entanglement of qubits to share a random string between Alice and Bob, which is further used as a key to encrypt messages. Since the protocol can be attacked by MRAs, checking bits are used for implementing MRAD.

In our original protocol, according to Theorem 3, we showed that 15 checking bits are enough to have 99.5% attack detection success rate if we assume the adversary Eve has 60.0% probability of attacking a single qubit sent by Alice. A much better protocol security level can be reached by adding only a few extra bits (for example, 20 checking bits can achieve a detection success rate of 99.92%). If the message size is sufficiently long, then the qubit efficiency can reach almost 100%.

The rate estimation version, the protocol RENKSQDC, can function properly and correctly detect attacks perpetrated by Eve while the qubits may be disturbed during the transmission. We designed a test to monitor the difference of κ, the probability that a qubit is disturbed or attacked, and ω (estimated or pre-known), the probability that a qubit is disturbed. If the difference is significantly large, the protocol terminates. The simulation results show that 60 checking bits can push detection success rate to almost 100% (assuming that $\alpha = 0.05$ and $\omega = 0.05$) if ω is unknown. The number of checking bits can decrease to 40 and achieve the same success rate if ω is pre-known. If we assume that MRAs are always detectable, then our protocol is secure against network attacks (Theorems 4 and 7).

References

1. Shor, P. W. & Preskill, J. Simple proof of security of the bb84 quantum key distribution protocol. *Phys. Rev. Lett.* **85**, 441–444 (2000).

2. Ekert, A. K. Quantum cryptography based on bell's theorem. *Phys. Rev. Lett.* **67**, 661–663 (1991).

3. Long, G. L. & Liu, X. S. Theoretically efficient high-capacity quantum-key-distribution scheme. *Phys. Rev. A* **65**, 032302 (2002).

4. Lo, H.-K. & Chau, H. F. Unconditional security of quantum key distribution over arbitrarily long distances. *Sci.* **283**, 2050–2056 (1999).

5. Shor, P. W. Polynomial-time algorithms for prime factorization and discrete logarithms on a quantum computer. *SIAM J. on Comput.* **26**, 1484–1509 (1997).

6. Boyer, M., Kenigsberg, D. & Mor, T. Quantum key distribution with classical bob. *Phys. Rev. Lett.* **99**, 140501 (2007).

7. Boyer, M., Gelles, R., Kenigsberg, D. & Mor, T. Semiquantum key distribution. *Phys. Rev. A* **79**, 032341 (2009).

8. Jian, W., Sheng, Z., Quan, Z. & Chao-Jing, T. Semiquantum key distribution using entangled states. *Chin. Phys. Lett.* **28**, 100301 (2011).

9. Li, Q., Chan, W. H. & Zhang, S. Semiquantum key distribution with secure delegated quantum computation. *Sci. Reports* **6** (2016).

10. Luo, Y.-P. & Hwang, T. Authenticated semi-quantum direct communication protocols using bell states. *Quantum Inf. Process.* **15**, 947–958 (2016).

11. Almousa, S. & Barbeau, M. Delay and reflection attacks in authenticated semi-quantum direct communications. *2016 IEEE Globecom Work. (GC Wkshps)* 1–7 (2016).

12. Zhong, M. *et al.* Optically addressable nuclear spins in a solid with a six-hour coherence time. *Nat.* **517**, 177–180 (2015).

13. Inagaki, T., Matsuda, N., Tadanaga, O., Asobe, M. & Takesue, H. Entanglement distribution over 300 km of fiber. *Opt. Express* 23241–23249.

14. Dai, H.-N. *et al.* Generation and detection of atomic spin entanglement in optical lattices. *Nat Phys* **12**, 783–787 (2016).

15. Neumann, P. *et al.* Quantum register based on coupled electron spins in a room-temperature solid. *Nat Phys* **6**, 249–253 (2010).

16. Lu, H., Barbeau, M. & Nayak, A. Economic no-key semi-quantum direct communication protocol. In *2017 IEEE Globecom Workshops (GC Wkshps)*, 1–7 (2017).

17. Malladi, S., Alves-Foss, J. & Heckendorn, R. B. On preventing replay attacks on security protocols. *In Proc. Int. Conf. on Secur. Manag.* 77–83 (2002).

18. Hogg, R., Tanis, E. & Zimmerman, D. *Probability and Statistical Inference*, 192 (Pearson Education, 2014).

19. DasGupta, A. *Normal Approximations and the Central Limit Theorem*, 213–242 (Springer New York, New York, NY, 2010).

20. Zou, X., Qiu, D., Li, L., Wu, L. & Li, L. Semiquantum-key distribution using less than four quantum states. *Phys. Rev. A* **79**, 052312 (2009).

Author Contributions

H.L devised the protocol. H.L and M.B wrote the main manuscript, and A.N gave solid suggestions on the manuscript. All authors reviewed the manuscript.

Additional Information

Competing interests: The authors declare no competing interests.

Table 1. Bell measurements on non-Bell states ($|00\rangle, |01\rangle, |10\rangle, |11\rangle$), each output has 50% probability.

$\lambda_A\lambda_B$	Output (b_1b_2)	$\lambda_A\lambda_B$	Output (b_1b_2)
$\|00\rangle$	00	$\|01\rangle$	01
	10		11
$\|10\rangle$	01	$\|11\rangle$	00
	11		10

Table 2. Comparisons among typical SQKD and SQDC protocols.

Protocols (The protocols using entanglements are marked by *)	Qubit permutation	Basis Switch	Qubit Efficiency η	Minimum # quregisters	EPT
Boyer(2009) Randomization-Based SQKD [7]	Yes	Yes	$< 12.5\%$	$4n$	0
Boyer(2009) Measure-Resend SQKD [7]	No	Yes	$< 12.5\%$	0	0
Zou (2009) Protocol 5 [20]	No	Yes	$< 12.5\%$	0	0
Wang (2011) [8] *	Yes	Yes	$< 50\%$	$6n$	Worse than linear
Li (2016) [9]	No	Yes	$< 6.25\%$	0	0
Luo (2016) [10] *	Yes	Yes	12.5%	$20n$	Worse than linear
ENKSQDC *	No	No	$\approx 100\%$	1	$2T+P$
RENKSQDC *	No	No	$\approx 99\%$	1	$2T+P$

Original Version

ORIGINALITY REPORT

39%	5%	38%	3%
SIMILARITY INDEX	INTERNET SOURCES	PUBLICATIONS	STUDENT PAPERS

PRIMARY SOURCES

1 Haoye Lu, Michel Barbeau, Amiya Nayak. "Economic No-Key Semi-Quantum Direct Communication Protocol", 2017 IEEE Globecom Workshops (GC Wkshps), 2017 — 33%
Publication

2 Krawec, Walter O.. "Security of a semi-quantum protocol where reflections contribute to the secret key", Quantum Information Processing, 2016. — <1%
Publication

3 pdfs.semanticscholar.org — <1%
Internet Source

4 www.osapublishing.org — <1%
Internet Source

5 Qin Li, Wai Hong Chan, Shengyu Zhang. "Semiquantum key distribution with secure delegated quantum computation", Scientific Reports, 2016 — <1%
Publication

Wei Zhang, Daowen Qiu, Paulo Mateus.

6	"Security of a single-state semi-quantum key distribution protocol", Quantum Information Processing, 2018<br>Publication	<1%
7	people.eecs.berkeley.edu<br>Internet Source	<1%
8	Steinert, S., F. Ziem, L. T. Hall, A. Zappe, M. Schweikert, N. Götz, A. Aird, G. Balasubramanian, L. Hollenberg, and J. Wrachtrup. "Magnetic spin imaging under ambient conditions with sub-cellular resolution", Nature Communications, 2013.<br>Publication	<1%
9	e3.physik.uni-dortmund.de<br>Internet Source	<1%
10	Han-Ning Dai, Bing Yang, Andreas Reingruber, Hui Sun, Xiao-Fan Xu, Yu-Ao Chen, Zhen-Sheng Yuan, Jian-Wei Pan. "Four-body ring-exchange interactions and anyonic statistics within a minimal toric-code Hamiltonian", Nature Physics, 2017<br>Publication	<1%
11	nparc.cisti-icist.nrc-cnrc.gc.ca<br>Internet Source	<1%
12	Chitra Shukla, Kishore Thapliyal, Anirban Pathak. "Semi-quantum communication:	<1%

protocols for key agreement, controlled secure direct communication and dialogue", Quantum Information Processing, 2017
Publication

13	nn%c3%a1inyoutube.com<br>Internet Source	<1%
14	LiLi Yan, YuHua Sun, Yan Chang, ShiBin Zhang, GuoGen Wan, ZhiWei Sheng. "Semi-quantum protocol for deterministic secure quantum communication using Bell states", Quantum Information Processing, 2018<br>Publication	<1%
15	eprints.soton.ac.uk<br>Internet Source	<1%
16	Submitted to Sim University<br>Student Paper	<1%
17	eprints.qut.edu.au<br>Internet Source	<1%
18	academic.uprm.edu<br>Internet Source	<1%
19	www.mdpi.com<br>Internet Source	<1%
20	Lecture Notes of the Institute for Computer Sciences Social Informatics and Telecommunications Engineering, 2010.<br>Publication	<1%

21	www.ma.huji.ac.il<br>Internet Source	<1%
22	d-nb.info<br>Internet Source	<1%
23	pt.scribd.com<br>Internet Source	<1%
24	Kun-Fei Yu, Jun Gu, Tzonelih Hwang, Prosanta Gope. "Multi-party semi-quantum key distribution-convertible multi-party semi-quantum secret sharing", Quantum Information Processing, 2017<br>Publication	<1%
25	"Statistical Trading Strategies", Springer Texts in Statistics, 2008<br>Publication	<1%
26	Submitted to Queen Mary and Westfield College<br>Student Paper	<1%
27	www.nature.com<br>Internet Source	<1%
28	www.scribd.com<br>Internet Source	<1%
29	Turkanović, Muhamed, and Marko Hölbl. "The (in)adequacy of applicative use of quantum cryptography in wireless sensor networks",	<1%

	Quantum Information Processing, 2014.<br>Publication	
30	www.bing.com<br>Internet Source	<1%
31	itex.coastal.cheswick.com<br>Internet Source	<1%
32	Butler, Richard, Timothy J. Dodwell, Raphael T. Haftka, Nam Ho Kim, Tatiana Kim, Stephanie Kynaston, and Rob Scheichl. "Uncertainty Quantification of Composite Structures with Defects using Multilevel Monte Carlo Simulations", 17th AIAA Non-Deterministic Approaches Conference, 2015.<br>Publication	<1%
33	www.amast.org<br>Internet Source	<1%
34	Zhulin Li, Qin Li, Chengdong Liu, Yu Peng, Wai Hong Chan, Lvzhou Li. "Limited resource semiquantum secret sharing", Quantum Information Processing, 2018<br>Publication	<1%
35	greenteapress.com<br>Internet Source	<1%
36	Lecture Notes in Computer Science, 2013.<br>Publication	<1%

37	www.dcc.ufla.br<br>Internet Source	<1%
38	Yu, Kun-Fei, Chun-Wei Yang, Ci-Hong Liao, and Tzonelih Hwang. "Authenticated semi-quantum key distribution protocol using Bell states", Quantum Information Processing, 2014.<br>Publication	<1%

Exclude quotes	Off	Exclude matches	Off
Exclude bibliography	Off		
